# Cleidocranial dysplasia caused by a novel de novo *RUNX2* splice-site variant

**DOI:** 10.1038/s41439-026-00353-3

**Published:** 2026-06-04

**Authors:** Hitoshi Kashiki, Jun Kido, Yohei Misumi, Keishin Sugawara, Naomi Tsuchida, Naomichi Matsumoto, Mitsuharu Ueda, Kimitoshi Nakamura

**Affiliations:** 1 Department of Pediatrics, Hitoyoshi Medical Center, Hitoyoshi, Japan; 2 Department of Pediatrics, Kumamoto University Hospital, Kumamoto, Japan; 3 Department of Pediatrics, Faculty of Life Sciences, Kumamoto University, Kumamoto, Japan; 4 Department of Neurology, Graduate School of Medical Sciences, Kumamoto University, Kumamoto, Japan; 5 Department of Human Genetics, Graduate School of Medicine, Yokohama City University, Yokohama, Japan; 6 Department of Rare Disease Genomics, Yokohama City University Hospital, Yokohama, Japan; 7 Department of Clinical Genetics, Yokohama City University Hospital, Yokohama, Japan; 8 Medical Genome Center, National Center of Neurology and Psychiatry, Kodaira, Tokyo Japan

**Keywords:** Diseases, Genetic counselling

## Abstract

Cleidocranial dysplasia is an autosomal dominant disorder caused by *RUNX2* variants. We report a Japanese boy with enlarged fontanelles in infancy, initially suspected of hypophosphatasia. Exome sequencing identified a novel de novo *RUNX2* splice-site variant (c.685+2T>G), affecting the canonical donor site, and predicted to disrupt splicing. This novel variant expands the mutational spectrum of *RUNX2*-associated cleidocranial dysplasia and highlights the value of early genetic diagnosis.

Cleidocranial dysplasia (CCD; OMIM no. 119600) is a rare autosomal dominant skeletal dysplasia caused by pathogenic variants in *RUNX2* (OMIM no. *600211)^1,2^, a master transcription factor essential for osteoblast differentiation and skeletal development. The estimated prevalence of CCD is approximately 1 in 1,000,000, although the true frequency may be underestimated owing to variable expressivity and generally preserved intellectual function.

Clinically, CCD is characterized by clavicular and cranial dysplasia together with dental anomalies, including retained deciduous teeth and supernumerary and/or unerupted permanent teeth, which may occasionally be associated with odontogenic cysts^3,4^. Craniofacial features typically include frontal bossing, midface hypoplasia, and hypertelorism. Additional skeletal findings may include pelvic widening, delayed pubic ossification, scoliosis, and brachydactyly^5,6^. The phenotypic spectrum is broad, and early clinical diagnosis in infancy can be challenging, particularly in cases presenting with isolated cranial suture abnormalities.

Early recognition of CCD is clinically important, as it allows appropriate surveillance for skeletal, dental, and potential otolaryngological complications, as well as genetic counseling. In particular, differentiation from other conditions associated with delayed fontanelle closure, such as hypophosphatasia, is essential in early infancy.

Here, we report a Japanese boy presenting with persistent cranial suture patency in early infancy, in whom a novel de novo splice-site variant in *RUNX2* (c.685+2T>G) was identified through the Initiative on Rare and Undiagnosed Diseases (IRUD)^7^. This case highlights the diagnostic challenges of CCD in early infancy and expands the mutational and clinical spectrum of *RUNX2*-associated disorders.

The patient was born at 39 weeks and 4 days of gestation by spontaneous vaginal delivery, with a birth weight of 3,348 g and Apgar scores of 9 and 10 at 1 min and 5 min, respectively. The antenatal course and neonatal period were unremarkable. Because of a markedly enlarged anterior fontanelle, he was referred to our hospital at ~2 weeks of age. Physical examination revealed an anterior fontanelle measuring 6 cm in diameter, widening of the sagittal suture (2.5 cm), and an enlarged posterior fontanelle. Chest and abdominal radiographs showed no skeletal abnormalities, and no rib anomalies or other structural malformations were identified. Laboratory testing revealed no thyroid dysfunction or hypocalcemia, but a decreased serum alkaline phosphatase level (126 U/l). At 2 months of age, serum calcium was 10.5 mg/dl, inorganic phosphate 5.6 mg/dl, and alkaline phosphatase (International Federation of Clinical Chemistry and Laboratory Medicine) remained low (126 U/l), whereas tissue-nonspecific alkaline phosphatase activity in dried blood spots was within the normal range (590.7 pmol/h/disk)^8^. Hypophosphatasia was therefore excluded. At 6 months of age, cranial CT showed persistent widening of the cranial sutures, and echocardiography revealed no structural cardiac abnormalities (Fig. 1A). Dental radiography demonstrated no evidence of tooth agenesis. Follow-up evaluation at 2 years and 4 months showed persistent delayed closure of the cranial sutures. Radiographic examination also revealed mild shortening of the distal phalanges, a narrow thoracic cage, and clavicular hypoplasia (Fig. 1B–F). Intact parathyroid hormone was 14 pg/ml, and 25-hydroxyvitamin D was 34.0 ng/ml.

**Fig. 1:**
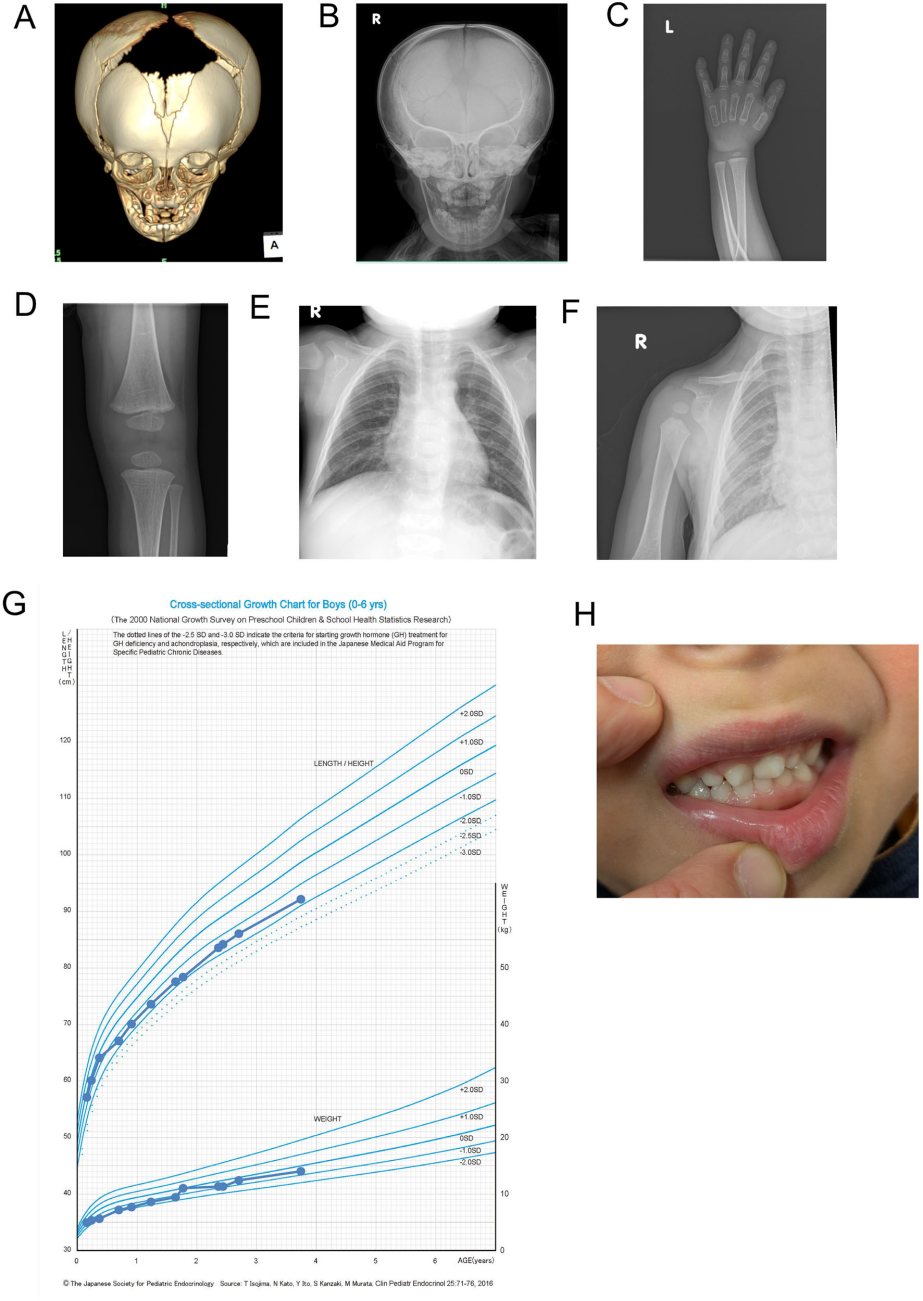
Clinical characteristics of the patient. **A**, Head CT imaging demonstrating persistent widening of the cranial sutures at 6 months of age. **B**, Skull radiograph showing open cranial sutures with a widened sagittal suture and delayed ossification of the calvarium at 2 years and 4 months of age. **C**, Radiograph of the left hand showing mild shortening of the distal phalanges without obvious bone deformities at 2 years and 4 months of age. Cone-shaped epiphyses are observed in the first distal phalanx, second metacarpal, second distal phalanx, and third distal phalanx. **D**, Radiograph of the left knee demonstrating no apparent abnormalities in the long bones or epiphyseal structures at 2 years and 4 months of age. **E**, Chest imaging revealing a narrow thoracic cage without rib anomalies at 2 years and 4 months of age. **F**, Radiograph of the right clavicle showing a hypoplastic clavicle at 2 years and 4 months of age. **G**, Growth curve of the patient. The patient’s height was approximately −1.5 SD relative to the standard for Japanese boys. **H**, Mild malalignment of the primary dentition with divergent alignment of the anterior teeth.

Developmental milestones, including head control, sitting, and independent walking, were achieved at appropriate ages, and language development was normal. The Enjoji Developmental Scale at 2 years and 4 months showed a developmental quotient of 102, with motor development exceeding chronological age.

At 2 years and 6 months, his height was 83.5 cm (−1.51 SD), weight was 11.2 kg (−0.82 SD), and head circumference was 52 cm (+2.0 SD). He showed mild short stature (Fig. 1G). Progressive enlargement of the head circumference had been noted over time. Brain MRI showed no hydrocephalus or intracranial space-occupying lesion.

He was enrolled in the IRUD program in Japan. Exome sequencing identified a heterozygous de novo splice-site variant in *RUNX2* (NM_001024630.4:c.685+2T>G) (Fig. 2). This variant affects the canonical splice donor site at the +2 position and is predicted to disrupt normal pre-mRNA splicing. *RUNX2* encodes a key transcription factor essential for skeletal and craniofacial development, and pathogenic variants in *RUNX2* are a well-established cause of CCD.

**Fig. 2:**
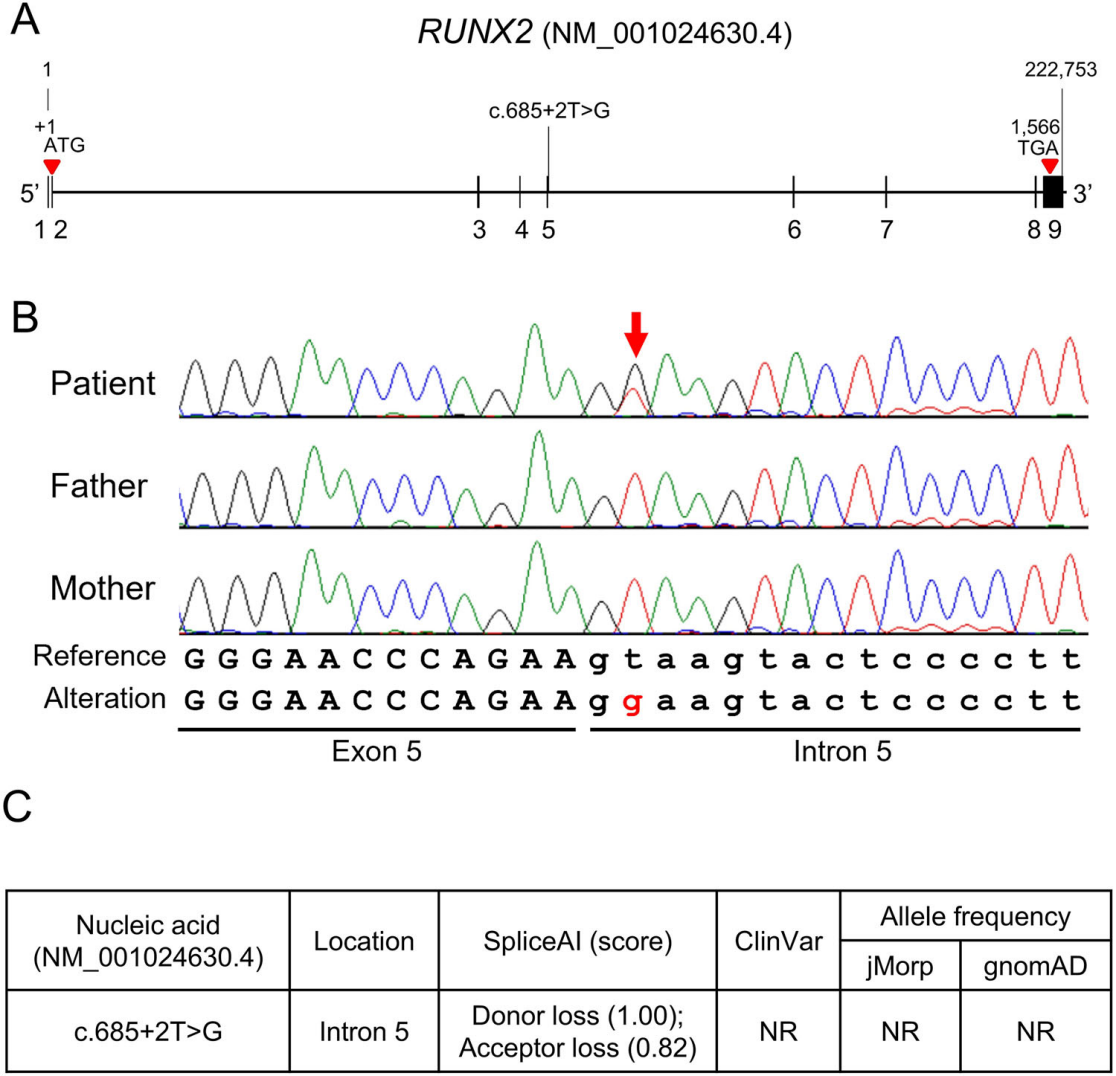
Identification of the *RUNX2* variant in the patient. **A**, Schematic representation of the *RUNX2* (NM_001024630.4) variant showing the location of the c.685+2T>G variant, at the canonical splice donor site of intron 5. **B**, Sanger sequencing confirmed the de novo *RUNX2* variant. The red arrow indicates the variant site. The patient harbored a heterozygous splice-site variant, c.685+2T>G, in *RUNX2*, whereas neither parent carried the variant, consistent with a de novo occurrence. Exonic and intronic sequences are shown in uppercase and lowercase letters, respectively. **C**, Summary of the identified variant. SpliceAI^18^ predicted loss of the canonical donor site and acceptor site (https://spliceailookup.broadinstitute.org/), which supports exon skipping. NR, not reported.

At the age of 3 years and 9 months, no intellectual disability or hearing impairment has been observed. Hypertelorism and short stature are present (Fig. 1G). All 20 primary teeth have erupted; however, mild malalignment of the primary dentition, including divergent alignment of the anterior teeth, was observed (Fig. 1H).

In this report, we describe a patient with CCD harboring a novel de novo splice-site variant in *RUNX2* (c.685+2T>G). Although functional validation such as transcript analysis was not performed, the canonical splice-site location (+2 position) strongly supports a loss-of-function effect consistent with *RUNX2* haploinsufficiency. The variant was absent from population databases and has not been previously reported, further supporting its classification as likely pathogenic according to the American College of Medical Genetics and Genomics and the Association for Molecular Pathology (ACMG/AMP) guidelines.

The patient exhibited characteristic features of CCD, including persistent cranial suture patency, hypertelorism, and clavicular hypoplasia. In addition, cone-shaped epiphyses in the hand were observed, consistent with skeletal abnormalities reported in CCD. These findings collectively support the clinical diagnosis and expand the phenotypic spectrum associated with *RUNX2* variants.

Notably, the patient presented in early infancy with markedly enlarged fontanelles and widening of the cranial sutures, raising a differential diagnosis that included hypophosphatasia. Although serum alkaline phosphatase levels were low, normal tissue-nonspecific alkaline phosphatase activity in dried blood spots allowed exclusion of hypophosphatasia. This case, therefore, underscores the importance of careful biochemical and genetic evaluation in infants presenting with delayed fontanelle closure.

The identified variant has not been registered in the ClinVar database^9^ or population databases, including gnomAD v4.1.0 (ref. ^10^) and the Tohoku Medical Megabank^11^, nor has it been reported in the literature. A different substitution at the same position (c.685+2T>C) has been predicted to markedly reduce exon inclusion^12^, and an adjacent donor variant (c.685+1G>T) has been experimentally shown to cause in-frame exon 5 skipping^13^, supporting a similar splicing defect for the present variant. Exon 5 encodes part of the Runt homology domain and nuclear localization signal, and loss of this region would be expected to impair RUNX2 function, consistent with the haploinsufficiency mechanism underlying CCD. According to the ACMG/AMP guidelines^14^ with ClinGen SVI refinements^15^, the variant was classified as likely pathogenic based on PVS1_Strong, PM2, PM6_Supporting, and PP4. However, this classification was primarily supported by predicted loss-of-function effects without functional validation, and the application of PP4 was considered with caution because the patient was still young and had not yet developed the full clinical spectrum of CCD.

On the basis of our literature review (Supplementary Data 1 and 2), which included 605 reported individuals with CCD, the frequencies of major clinical manifestations were as follows: short stature in 31.7% (192/605), open or delayed closure of the anterior fontanelle in 53.1% (321/605), facial dysmorphism in 29.9% (181/605), hearing loss in 1.8% (11/605), hypertelorism in 22.8% (138/605), low nasal bridge in 9.1% (55/605), oral abnormalities in 13.2% (80/605), dental hypoplasia or tooth formation defects in 55.2% (334/605), thoracic cage abnormalities in 61.0% (369/605), and skeletal abnormalities of the limbs and/or spine in 21.2% (128/605). Delayed closure of the anterior fontanelle was observed in approximately half of patients, whereas other characteristic features may become more evident with age. These findings highlight the difficulty of establishing a clinical diagnosis in early infancy and support the role of genetic testing in such cases^16,17^.

The predicted disruption of splicing and the de novo occurrence strongly support its pathogenic role. Future transcript-level analyses will be valuable for further elucidating the molecular consequences of this variant.

In conclusion, we describe a patient with CCD harboring a novel heterozygous *RUNX2* variant (c.685+2T>G). The patient exhibits typical clinical features of CCD, and additional skeletal and dental manifestations may become more apparent with age. Therefore, long-term multidisciplinary management, including dental and orthopedic follow-up, is warranted.

## HGV Database

The relevant data from this Data Report are hosted at the Human Genome Variation Database at 10.6084/m9.figshare.hgv.3660.

## Supplementary information

Supplementary Data 1. 　Reported RUNX2 variants associated with CCD

Supplementary Data 2. RUNX2 variants and associated CCD features

## Data Availability

Data will be made available on request.
