# Supplementary material for: Cleidocranial dysplasia caused by a novel de novo RUNX2 splice-site variant
Source: Hum Genome Var. 2026 Jun 4;13:19. doi: 10.1038/s41439-026-00353-3 (PMC13478401; doi:10.1038/s41439-026-00353-3)
Supplement: Supplementary file 1 [file 41439_2026_353_MOESM1_ESM.docx]

Supplementary Data 1. Reported *RUNX2* variants associated with CCD

| No. | Nucleic acid (NM_001024630.4) | Amino acid  (NP_001019801.3) | ClinVar  (accessed Feb. 2026) | Number of patients | Allele frequency | | References |
| --- | --- | --- | --- | --- | --- | --- | --- |
|  |  |  |  |  | jMorp  61K JPN | gnomAD  v4.1.0 |  |
| 1 | c.2T>C | p.Met1? | NR | 1 | NR | NR | 1 |
| 2 | c.29dupT | p.Thr11Aspfs*150 | Pathogenic | 2 | NR | NR | 2 |
| 3 | c.58+1G>A | - | NR | 1 | NR | NR | 3 |
| 4 | c.58+2T>A | - | NR | 1 | NR | NR | 4 |
| 5 | c.59-1G>T | - | NR | 1 | NR | NR | 5 |
| 6 | c.90dupC | p.Ser31Leufs*130 | Pathogenic | 4 | NR | NR | 2, 6, 7 |
| 7 | c.132delG | p.Val45Trpfs*99 | NR | 1 | NR | NR | 8 |
| 8 | c.133_134insGTCC | p.Val45Glyfs*117 | NR | 5 | NR | NR | 9 |
| 9 | c.148C>T | p.Gln50* | Pathogenic | 1 | NR | NR | 3 |
| 10 | c.158A>T | p.Gln53Leu | NR | 1 | NR | NR | 10 |
| 11 | c.166C>T | p.Gln56* | NR | 1 | NR | NR | 11 |
| 12 | c.169C>T | p.Gln57* | NR | 2 | NR | NR | 12 |
| 13 | c.178delC | p.Gln60Serfs*84 | NR | 1 | NR | NR | 9 |
| 14 | c.186_201dup | p.Gln68Alafs*98 | NR | 2 | NR | NR | 13 |
| 15 | c.193C>T | p.Gln65* | NR | 1 | NR | NR | 14 |
| 16 | c.196C>T | p.Gln66* | Pathogenic | 3 | NR | NR | 9 |
| 17 | c.199C>T | p.Gln67* | Pathogenic | 2 | NR | NR | 15 |
| 18 | c.203delAinsCG | p.Gln68Profs*93 | Likely pathogenic | 2 | NR | NR | 2 |
| 19 | c.207_208ins13 | p.Glu72Glnfs*93 | NR | 1 | NR | NR | 14 |
| 20 | c.213_214insCAGCAGCAGCAG | p.Gln71_Glu72insGlnGlnGlnGln | NR | 2 | NR | NR | 16 |
| 21 | c.220_342del | p.Ala74_Val114del | NR | 1 | NR | NR | 7 |
| 22 | c.227_306del | p.Ala76Glyfs*58 | NR | 1 | NR | NR | 17 |
| 23 | c.230_276del | p.Ala77Valfs*68 | NR | 1 | NR | NR | 14 |
| 24 | c.231_232delTG | p.Ala78Glyfs*82 | NR | 1 | NR | NR | 18 |
| 25 | c.231_239dup | p.Ala87_Ala89dup | Uncertain significance | 1 | NR | NR | 19 |
| 26 | c.240_260del21ins3 | p.Ala82_87del | NR | 1 | NR | NR | 2 |
| 27 | c.243_260del | p.Ala84_Ala89del | NR | 4 | 0.002734 | 0.045517 | 2, 20, 21, 22 |
| 28 | c.278T>A | p.Leu93* | Pathogenic | 3 | NR | NR | 23 |
| 29 | c.274delC | p.Arg92Glyfs*52 | Pathogenic | 1 | NR | NR | 14 |
| 30 | c.276_277ins17 | p.Leu93* | NR | 1 | NR | NR | 14 |
| 31 | c.278T>A | p.Leu93* | Pathogenic | 1 | NR | NR | 14 |
| 32 | c.278insT | p.Leu93Phefs*68 | NR | 1 | NR | NR | 24 |
| 33 | c.282delG | p.Pro95Argfs*49 | NR | 1 | NR | NR | 14 |
| 34 | c.296insA | p.Asn99Lysfs*62 | NR | 4 | NR | NR | 25 |
| 35 | c.329delC | p.Pro110Argfs*34 | Pathogenic | 1 | NR | NR | 14 |
| 36 | c.334G>T | p.Glu112* | NR | 1 | NR | NR | 3 |
| 37 | c.338T>G | p.Leu113Arg | NR | 1 | NR | NR | 15 |
| 38 | c.338T>G/366_367GT>TC | p.Leu113Arg and p.Cys123Arg | NR | 1 | NR | NR | 13 |
| 39 | c.346T>A | p.Trp116Arg | NR | 1 | NR | NR | 26 |
| 40 | c.353G>A | p.Ser118Asn | NR | 1 | NR | NR | 14 |
| 41 | c.354C>A | p.Ser118Arg | Likely pathogenic | 5 | NR | NR | 2, 25 |
| 42 | c.354C>G | p.Ser118Arg | NR | 1 | NR | NR | 13 |
| 43 | c.371C>G | p.Ser124Trp | Uncertain significance | 3 | NR | NR | 27 |
| 44 | c.379C>T | p.Pro127Ser | Likely pathogenic | 2 | NR | NR | 28 |
| 45 | c.382insT | p.Ser128Phefs*33 | NR | 1 | NR | NR | 29 |
| 46 | c.383C>A | p.Ser128* | NR | 1 | NR | NR | 14 |
| 47 | c.386A>G | p.His129Arg | Uncertain significance | 1 | NR | NR | 24 |
| 48 | c.389G>A | p.Trp130* | Pathogenic | 2 | NR | NR | 4 |
| 49 | c.391C>A | p.Arg131Ser | Uncertain significance | 1 | NR | NR | 14 |
| 50 | c.391C>T | p.Arg131Cys | NR | 3 | NR | NR | 14, 30, 31 |
| 51 | c.391C>G | p.Arg131Gly | Likely pathogenic | 1 | NR | NR | 3 |
| 52 | c.396C>A | p.Cys132* | NR | 1 | NR | NR | 14 |
| 53 | c.397_399delAAC | p.Asn133del | NR | 1 | NR | NR | 4 |
| 54 | c.398_399insACAGCAGCAGCAGCA and c.411_412insG | p.Asn133delinsLysGlnGlnGlnGlnHis/p.Val138Glyfs*23 | NR | 1 | NR | NR | 32 |
| 55 | c.399_401delCAA | p.Asn134del | NR | 1 | NR | NR | 7 |
| 56 | c.407T>A | p.Leu136Gln | NR | 1 | NR | NR | 30 |
| 57 | c.407T>C | p.Leu136Pro | Pathogenic | 12 | NR | NR | 14, 33 |
| 58 | c.423+2delT | - | Likely pathogenic | 3 | NR | NR | 2 |
| 59 | c.424-1G>T | - | NR | 1 | NR | NR | 5 |
| 60 | c.425insT | p.Val143Glyfs*18 | NR | 1 | NR | NR | 34 |
| 61 | c.436G>A | p.Gly146Arg | Conflicting classifications of pathogenicity | 2 | NR | NR | 12 |
| 62 | c.443_454delTACCAGATGGGAinsG | p.Val148Glyfs*9 | Pathogenic | 1 | NR | NR | 2 |
| 63 | c.463A>C | p.Thr155Pro | NR | 1 | NR | NR | 35 |
| 64 | c.467T>A | p.Val156Asp | Likely pathogenic | 1 | NR | NR | 14 |
| 65 | c.469insA | p.Met157Asnfs*4 | NR | 1 | NR | NR | 14 |
| 66 | c.470insT | p.Met157Ilefs*4 | NR | 1 | NR | NR | 36 |
| 67 | c.471_472dupGG | p.Ala158Glyfs*19 | NR | 1 | NR | NR | 17 |
| 68 | c.473C>A | p.Ala158Glu | NR | 3 | NR | NR | 37, 128 |
| 69 | c.475G>C | p.Gly159Arg | NR | 2 | NR | NR | 38 |
| 70 | c.476G>A | p.Gly159Asp | Pathogenic/Likely pathogenic | 1 | NR | NR | 39 |
| 71 | c.476delG | p.Gly159fs*175 | NR | 1 | NR | NR | 40 |
| 72 | c.480C>G | p.Asn160Lys | Likely pathogenic | 1 | NR | NR | 30 |
| 73 | c.481_482delGA | p.Asp161* | NR | 1 | NR | NR | 13 |
| 74 | c.484G>T | p.Glu162* | NR | 1 | NR | NR | 14 |
| 75 | c.495delT | A166Lfs*10 | Likely pathogenic | 2 | NR | NR | 9 |
| 76 | c.497_506del | p.Ala166Glyfs*7 | NR | 1 | NR | NR | 41 |
| 77 | c.505C>T | p.Arg169Trp | Pathogenic/Likely pathogenic | 3 | NR | NR | 2, 42 |
| 78 | c.506G>A | p.Arg169Gln | Conflicting classifications of pathogenicity | 1 | NR | NR | 7 |
| 79 | c.506G>C | p.Arg169Pro | Pathogenic | 2 | NR | NR | 43 |
| 80 | c.514delT | p.Ser172Leufs*4 | NR | 2 | NR | NR | 44 |
| 81 | c.522insA | p.Met175fs*218 | NR | 1 | NR | NR | 4 |
| 82 | c.523A>G | p.Met175Val | Conflicting classifications of pathogenicity | 1 | NR | 0.000066 | 14 |
| 83 | c.524T>A | p.Met175Lys | NR | 1 | NR | NR | 14 |
| 84 | c.524T>G | p.Met175Arg | Likely pathogenic | 5 | NR | NR | 7, 13, 45, 46 |
| 85 | c.532C>T | p.Gln178* | NR | 3 | NR | NR | 47 |
| 86 | c.532delC | p.Gln178Lysfs*2 | NR | 1 | NR | NR | 5 |
| 87 | c.534dupA | p.Val179Serfs*40 | NR | 1 | NR | NR | 129 |
| 88 | c.538_539delincCA | p.Ala180Gln | NR | 1 | NR | NR | 77 |
| 89 | c.540_549del10 | p.Arg181Ilefs*2 | NR | 1 | NR | NR | 13 |
| 90 | c.549delC | p.Asn183fs*185 | NR | 2 | NR | NR | 48 |
| 91 | c.553_554delCT | p.Leu185Glufs*33 | NR | 1 | NR | NR | 7 |
| 92 | c.557G>C | p.Arg186Thr | NR | 1 | NR | NR | 15 |
| 93 | c.560T>C | p.Phe187Ser | NR | 1 | NR | NR | 14 |
| 94 | c.568C>T | p.Arg190Trp | Pathogenic | 30 | NR | NR | 4, 9, 12, 25, 30, 39, 49, 50, 51, 52, 53, 54,55, 56 |
| 95 | c.569G>A | p.Arg190Gln | Pathogenic | 15 | NR | NR | 1, 2, 12, 24, 25, 44, 46, 57, 58, 59 |
| 96 | c.569G>C | p.Arg190Pro | NR | 1 | NR | NR | 25 |
| 97 | c.572G>A | p.Ser191Asn | Likely pathogenic | 3 | NR | NR | 7, 23, 45 |
| 98 | c.574G>A | p.Gly192Arg | NR | 4 | NR | NR | 18, 60 |
| 99 | c.577C>T | p.Arg193* | Pathogenic | 19 | NR | NR | 2, 5, 9, 12, 13, 18, 25, 52, 61, 62, 63 |
| 100 | c.577C>G | p.Arg193Gly | NR | 2 | NR | NR | 2, 64 |
| 101 | c.577_579delCGAinsTGT | p.Arg193Cys | NR | 1 | NR | NR | 7 |
| 102 | c.578G>A | p.Arg193Gln | Pathogenic/Likely pathogenic | 3 | NR | NR | 14, 65, 66 |
| 103 | c.578G>C | p.Arg193Pro | NR | 1 | NR | NR | 67 |
| 104 | c.580+1G>A | - | NR | 3 | NR | NR | 68 |
| 105 | c.580+1G>T | - | NR | 2 | NR | NR | 14, 30 |
| 106 | c.580+2T>C | - | NR | 2 | NR | NR | 9 |
| 107 | c.580+3delGTAA | - | NR | 2 | NR | NR | 9 |
| 108 | c.581-9T>G | - | NR | 5 | NR | NR | 69 |
| 109 | c.590T>C | p.Phe197Ser | Pathogenic | 4 | NR | NR | 5, 9 |
| 110 | c.592delA | p.Thr198Profs*2 | NR | 2 | NR | NR | 6 |
| 111 | c.593_601delCCTTGACCA | p.Thr198_Thr200del | NR | 1 | NR | NR | 70 |
| 112 | c.594_595delCTinsG | p.Leu199* | Pathogenic | 1 | NR | NR | 2 |
| 113 | c.596G>A | p.Arg190Gln | NR | 2 | NR | NR | 71 |
| 114 | c.597G>T | p.Leu199Phe | Likely pathogenic | 1 | NR | NR | 7 |
| 115 | c.598A>G | p.Thr200Ala | Pathogenic | 3 | NR | NR | 7 |
| 116 | c.599C>T | p.Thr200Ile | NR | 3 | NR | NR | 14, 44, 72 |
| 117 | c.604delA | p.Thr202Profs*9 | Pathogenic | 2 | NR | NR | 24 |
| 118 | c.610A>T | p.Lys 204* | NR | 1 | NR | NR | 26 |
| 119 | c.614C>G | p.Thr205Arg | NR | 5 | NR | NR | 2, 13 |
| 120 | c.624_625dup | p.Gln209Profs*3 | NR | 1 | NR | NR | 14 |
| 121 | c.625C>T | p.Gln209* | Pathogenic | 1 | NR | NR | 73 |
| 122 | c.626A>G | p.Gln209Arg | NR | 1 | NR | NR | 7 |
| 123 | c.627A>T | p.Gln209His | NR | 1 | NR | NR | 14 |
| 124 | c.628C>T | p.Pro224Ser | NR | 4 | NR | NR | 74 |
| 125 | c.631G>C | p.Ala211Pro | Uncertain significance | 1 | NR | NR | 14 |
| 126 | c.631_633dup | p.Ala211dup | NR | 1 | NR | NR | 14 |
| 127 | c.634T>G | p.Thr212Pro | NR | 2 | NR | NR | 75 |
| 128 | c.635_638delCCTA | p.Thr212Ilefs*9 | NR | 4 | NR | NR | 76 |
| 129 | c.636delC | p.Tyr213Ilefs*9 | NR | 1 | NR | NR | 7 |
| 130 | c.636dupC | p.Tyr213Leufs*6 | Pathogenic | 4 | NR | NR | 2 |
| 131 | c.644delG | p.Arg215Lysfs*7 | NR | 2 | NR | NR | 44, 72 |
| 132 | c.651_652delTA | p.Lys218Serfs*17 | NR | 1 | NR | NR | 77 |
| 133 | c.652A>C | p.Lys218Gln | Likely pathogenic | 1 | NR | NR | 14 |
| 134 | c.652A>G | p.Lys218Glu | Pathogenic | 3 | NR | NR | 14, 30 |
| 135 | c.654A>T | p.Lys218Asn | NR | 4 | NR | NR | 9 |
| 136 | c.659C>T | p.Thr220Ile | Pathogenic | 3 | NR | NR | 9 |
| 137 | c.659C>G | p.Thr220Arg | NR | 1 | NR | NR | 30 |
| 138 | c.662T>A | p.Val221Glu | NR | 1 | NR | NR | 77 |
| 139 | c.662T>G | p.Val221Gly | NR | 3 | NR | NR | 4 |
| 140 | c.667G>A | p.Gly223Arg | NR | 1 | NR | NR | 14 |
| 141 | c.668G>T | p.Gly223Val | NR | 4 | NR | NR | 18 |
| 142 | c.673C>T | p.Arg225Trp | Pathogenic | 13 | NR | NR | 4, 9, 12, 13, 15, 18, 23, 30, 78, 79, 80 |
| 143 | c.674G>A | p.Arg225Gln | Pathogenic | 66 | NR | NR | 2, 3, 7, 9, 12, 13, 24, 25, 29, 39, 46, 53, 64, 71, 77, 79, 80, 81, 82, 83, 84, 85, 86, 87, 88, 89, 90, 91, 125, 126, 127 |
| 144 | c.674G>T | p.Arg225Leu | not provided | 9 | NR | NR | 14, 25, 44, 63, 72 |
| 145 | c.685+4delAAGT | - | NR | 1 | NR | NR | 88 |
| **146** | **c.685+2T>G** | **-** | **NR** | **1** | **NR** | **NR** | **This study** |
| 147 | c.685+5G>A | - | Uncertain significance | 2 | NR | NR | 2, 92 |
| 148 | c.682A>G | p.Arg228Gly | Uncertain significance | 1 | NR | NR | 14 |
| 149 | c.694C>T | p.Gln232* | NR | 7 | NR | NR | 14, 2 |
| 150 | c.716delC | p.Pro239Leufs*11 | NR | 2 | NR | NR | 24 |
| 151 | c.718_721del | p.Ser240Cysfs*9 | NR | 1 | NR | NR | 14 |
| 152 | c.722delT | p.Leu241Cysfs*9 | NR | 1 | NR | NR | 93 |
| 153 | c.722_725delTGTT | p.Leu241Serfs*8 | NR | 1 | NR | NR | 18 |
| 154 | c.739delA | p.Ser247Valfs*3 | Pathogenic | 1 | NR | NR | 80 |
| 155 | c.751C>T | p.Arg251Cys | NR | 1 | NR | 0.000007 | 22 |
| 156 | c.774_779delAGTAGGinsGA | p.Val259Metfs*11 | NR | 1 | NR | NR | 64 |
| 157 | c.821delC | p.Pro274Glnfs*34 | NR | 2 | NR | NR | 50 |
| 158 | c.821_822delCA | p.Pro274Glnfs*4 | NR | 2 | NR | NR | 25 |
| 159 | c.824delG | p.Ser275Ilefs*33 | NR | 1 | NR | NR | 13 |
| 160 | c.831delT | p.Phe277Leufs*31 | NR | 1 | NR | NR | 14 |
| 161 | c.838C>T | p.Gln280* | Pathogenic | 4 | NR | NR | 9 |
| 162 | c.844C>T | p.Gln282* | NR | 3 | NR | NR | 94 |
| 163 | c.859G>A | p.Asp287Asn | NR | 1 | NR | NR | 14 |
| 164 | c.859+1G>T | - | NR | 1 | NR | NR | 7 |
| 165 | c.860-2A>G | - | NR | 11 | NR | NR | 95 |
| 166 | c.860-2A>C | - | NR | 2 | NR | NR | 2, 24, 96 |
| 167 | c.860-1G>C | - | NR | 1 | NR | NR | 97 |
| 168 | c.864insC | p.Arg289Glnfs*12 | NR | 3 | NR | NR | 98 |
| 169 | c.868C>T | p.Gln290* | Pathogenic | 2 | NR | NR | 25 |
| 170 | c.874_875delCA | p.Gln292Valfs*8 | Pathogenic | 4 | NR | NR | 4, 71 |
| 171 | c.878C>T | p.Gln290* | NR | 1 | NR | NR | 24 |
| 172 | c.879_885del | p.Ser294Argfs*12 | NR | 1 | NR | NR | 14 |
| 173 | c.884delC | p.Pro295Argfs*13 | Pathogenic | 3 | NR | NR | 7, 99 |
| 174 | c.887insC | p.Trp297Valfs*4 | NR | 2 | NR | NR | 6 |
| 175 | c.887delC | p.Pro296Argfs*12 | NR | 1 | NR | NR | 13 |
| 176 | c.891G>A | p.Trp297* | Pathogenic | 3 | NR | NR | 13, 39, 41 |
| 177 | c.895T>C | p.Tyr299His | NR | 1 | NR | NR | 100 |
| 178 | c.897T>G | p.Tyr299* | NR | 1 | NR | NR | 101 |
| 179 | c.901C>T | p.Gln301* | Pathogenic | 2 | NR | NR | 80 |
| 180 | c.909C>A | p.Tyr303* | NR | 1 | NR | NR | 67 |
| 181 | c.909C>G | p.Tyr303* | Pathogenic | 2 | NR | NR | 18 |
| 182 | c.915delC | p.Tyr306Thrfs*2 | NR | 1 | NR | NR | 13 |
| 183 | c.938C>T | p.Pro313Leu | NR | 2 | NR | 0.000026 | 102 |
| 184 | c.938delC | p.Pro313Argfs*171 | Pathogenic | 1 | NR | NR | 12 |
| 185 | c.947delA | p.His316Profs*168 | NR | 1 | NR | NR | 103 |
| 186 | c.950_971del | p.Ser317Tyrfs*160 | NR | 1 | NR | NR | 97 |
| 187 | c.977delG | p.Gly326Alafs*158 | NR | 1 | NR | NR | 14 |
| 188 | c.1000G>A | p.Asp334Asn | Uncertain significance | 1 | 0.000024 | NR | 22 |
| 189 | c.1019del | p.Ser340* | NR | 1 | NR | NR | 101 |
| 190 | c.1056G>A | p.Trp352* | NR | 1 | NR | NR | 14 |
| 191 | c.1061insT | p.Ser354Phefs*5 | NR | 5 | NR | NR | 104 |
| 192 | c.1064C>T | p.Thr355Ile | NR | 1 | NR | NR | 105 |
| 193 | c.1081C>T | p.Gln361* | Pathogenic | 1 | NR | NR | 80 |
| 194 | c.1085C>T | p.Ala362Val | Pathogenic | 1 | NR | NR | 9 |
| 195 | c.1088-1G>C | - | NR | 1 | NR | NR | 14 |
| 196 | c.1088G>T | p.Gly363Val | Likely pathogenic | 3 | NR | NR | 2 |
| 197 | c.1096G>T | p.Glu366* | NR | 2 | NR | NR | 106 |
| 198 | c.1111dupT | p.Ser371Phefs*14 | NR | 1 | NR | NR | 107 |
| 199 | c.1111_1129del19 | p.Ser371Glnfs*107 | NR | 1 | NR | NR | 4 |
| 200 | c.1119insC | p.Arg374Glnfs*11 | NR | 1 | NR | NR | 108 |
| 201 | c.1119delC | p.Arg374Glyfs*110 | NR | 3 | NR | NR | 39, 53, 81 |
| 202 | c.1122delG | p.Arg374Serfs*110 | NR | 3 | NR | NR | 25 |
| 203 | c.1124insA | p.Phe376Valfs*9 | NR | 1 | NR | NR | 29 |
| 204 | c.1127insT | p.Ser378Lysfs*7 | NR | 1 | NR | NR | 13 |
| 205 | c.1132delA | p.Ser378Alafs*106 | NR | 1 | NR | NR | 25 |
| 206 | c.1153_1154delGA | p.Ser385Profs*104 | NR | 1 | NR | NR | 14 |
| 207 | c.1157delG | p.Arg386Profs*98 | NR | 2 | NR | NR | 12, 13 |
| 208 | c.1169delC | p.Pro390Hisfs*94 | NR | 1 | NR | NR | 14 |
| 209 | c.1171C>T | p.Arg391* | Pathogenic | 19 | NR | NR | 3, 4, 7, 5, 24, 39, 46, 53, 79, 81, 88, 109 |
| 210 | c.1182T>A | p.Tyr394* | Pathogenic | 1 | NR | NR | 110 |
| 211 | c.1199-1200insA | p.Tyr400* | NR | 1 | NR | NR | 64 |
| 212 | c.1205delC | p.Pro402Argfs*82 | NR | 1 | NR | NR | 14 |
| 213 | c.1205dupC | p.Pro403Alafs*87 | NR | 1 | NR | NR | 13 |
| 214 | c.1215dupC | p.Ser406Leufs*84 | NR | 1 | NR | NR | 97 |
| 215 | c.1228dupC | p.Leu410Profs*80 | Pathogenic | 1 | NR | NR | 111 |
| 216 | c.1246_1247insACCC | p.Thr416Asnfs*75 | NR | 1 | NR | NR | 114 |
| 217 | c.1250insA | p.His417Glnfs*73 | NR | 1 | NR | NR | 24 |
| 218 | c.1250_1251delCA | p.His417Leufs*72 | NR | 1 | NR | NR | 14 |
| 219 | c.1254C>A | p.Tyr418* | NR | 1 | NR | NR | 14 |
| 220 | c.1259C>A | p.Thr420Asn | NR | 1 | NR | NR | 14 |
| 221 | c.1259C>T | p.Thr420Ile | NR | 4 | NR | NR | 112, 113 |
| 222 | C.1271_1272insTACCTACCTGTACACTCACT | p.Pro425Thrfs*66 | NR | 1 | NR | NR | 39 |
| 223 | c.1281delC | p.Gly428Alafs*56 | Pathogenic | 5 | NR | NR | 2 |
| 224 | c.1321dupT | p.Ser370Phefs*13 | NR | 1 | NR | NR | 17 |
| 225 | c.1335_1336delTC | p.Tyr446Leufs*43 | NR | 1 | NR | NR | 14 |
| 226 | c.1361A>G | p.Tyr454Cys | Uncertain significance | 1 | NR | NR | 22 |
| 227 | c.1379dupC | p.Asp463Argfs*27 | Pathogenic/Likely pathogenic | 1 | NR | NR | 13 |
| 228 | c.1381G>C | p.Gly461Arg | NR | 1 | 0.000033 | 0.000026 | 28 |
| 229 | c.1384G>T | p.Gly462* | NR | 1 | NR | NR | 46 |
| 230 | c.1385dupG | p.Asp463Argfs*27 | Pathogenic | 1 | NR | NR | 14 |
| 231 | c.1531G>A | p.Gly511Ser | Benign/Likely benign | 1 | 0.000008 | 0.008299 | 22 |
| 232 | c.1550_1553delTTTG | p.Val517Glyfs*61 | Pathogenic | 6 | NR | NR | 12, 24, 39, 115 |
| 233 | c.1552delT | p.Trp518Glyfs*60 | NR | 1 | NR | NR | 116 |
| 234 | c.1554delG | p.Trp518Cysfs*61 | Pathogenic | 2 | NR | NR | 117 |
| 235 | c.1565G>C | p.*522Serext*23 | Pathogenic | 1 | NR | NR | 10 |
| 236 | Large deletion | exon 0~7, 3’UTR deletion | NR | 1 | NR | NR | 53 |
| 237 | Large deletion | exon 1 to exon 3 deletion | NR | 3 | NR | NR | 118 |
| 238 | Large deletion | exon 1 to exon 5 deletion | NR | 5 | NR | NR | 25 |
| 239 | Large deletion | exon 2 deletion | NR | 1 | NR | NR | 119 |
| 240 | Large deletion | exon 2 to exon 6 deletion | NR | 8 | NR | NR | 120 |
| 241 | Large deletion | exon 2 to exon 9 deletion | NR | 1 | NR | NR | 2 |
| 242 | Large deletion | exon 3 to exon 9 deletion | NR | 1 | NR | NR | 2 |
| 243 | Large deletion | exon 6 to exon 9 deletion | NR | 1 | NR | NR | 2 |
| 244 | Large deletion | exon 2 to exon 9 deletion | NR | 1 | NR | NR | 121 |
| 245 | Large deletion | exon 4 to intron 7 deletion | NR | 1 | NR | NR | 122 |
| 246 | Large deletion | 498 kb deletion | NR | 1 | NR | NR | 123 |
| 247 | Large deletion | 344 kb deletion | NR | 1 | NR | NR | 124 |

References

1. Yang L, Lu G, Shen W, Chen W, Lu H, Zhang G *et al*. Whole-exome sequencing of a novel initiation codon mutation in *RUNX2* in a Chinese family with cleidocranial dysplasia. *Medicine* 2021; **100**: e27746.
2. Berkay EG, Elkanova L, Kalaycı T, Alkaya DU, Altunoglu U, Cefle K *et al*. Skeletal and molecular findings in 51 cleidocranial dysplasia patients from Turkey. *Am J Med Genet A* 2021; **185**: 2488-2495.
3. Kim HJ, Nam SH, Kim HJ, Park HS, Ryoo HM, Kim SY *et al*. Four novel *RUNX2* mutations including a splice donor site result in the cleidocranial dysplasia phenotype. *J Cell Physiol* 2006; **207**: 114-122.
4. Tessa A, Salvi S, Casali C, Garavelli L, Digilio MC, Dotti MT *et al*. Six novel mutations of the *RUNX2* gene in Italian patients with cleidocranial dysplasia. *Hum Mutat* 2003; **22**: 104.
5. Zhang YW, Yasui N, Kakazu N, Abe T, Takada K, Imai S *et al*. *PEBP2*a*A*/*CBFA1* mutations in Japanese cleidocranial dysplasia patients. *Gene* 2000; **244**: 21-28.
6. Huang Y, Song Y, Zhang C, Chen G, Wang S, Bian Z. Novel RUNX2 frameshift mutations in Chinese patients with cleidocranial dysplasia. *Eur J Oral Sci* 2013; **121**: 142-147.
7. Zhou G, Chen Y, Zhou L, Thirunavukkarasu K, Hecht J, Chitayat D *et al*. *CBFA1* mutation analysis and functional correlation with phenotypic variability in cleidocranial dysplasia. *Hum Mol Genet* 1999; **8**: 2311-2316.
8. Pan Y, Lu W, Meng W, Liao W, Hu A, Wu B, Xiong F. A novel single-base deletion of the RUNX Family Transcription Factor 2 gene associated with cleidocranial dysplasia. *Eur J Oral Sci* 2023; **131**: e12910.
9. Yoshida T, Kanegane H, Osato M, Yanagida M, Miyawaki T, Ito Y, Shigesada K. Functional analysis of *RUNX2* mutations in Japanese patients with cleidocranial dysplasia demonstrates novel genotype-phenotype correlations. *Am J Hum Genet* 2002; **71**: 724-738.
10. [Machuca-Tzili](https://pubmed.ncbi.nlm.nih.gov/?term=Machuca-Tzili+L&cauthor_id=12081718) L, [Monroy-Jaramillo](https://pubmed.ncbi.nlm.nih.gov/?term=Monroy-Jaramillo+N&cauthor_id=12081718) N, [González-del Angel](https://pubmed.ncbi.nlm.nih.gov/?term=Gonz%C3%A1lez-del+Angel+A&cauthor_id=12081718) A, [Kofman-Alfaro](https://pubmed.ncbi.nlm.nih.gov/?term=Kofman-Alfaro+S&cauthor_id=12081718) S. New mutations in the *CBFA1* gene in two Mexican patients with cleidocranial dysplasia. *Clin Genet* 2002; **61**: 349-353.
11. Singh A, Goswami M, Pradhan G, Han MS, Choi JY, Kapoor S. Cleidocranial dysplasia with normal clavicles: A report of a novel genotype and a review of seven previous cases. *Mol Syndromol* 2015; **6**: 83-86.
12. Baumert U, Golan I, Redlich M, Aknin JJ, Muessig D. Cleidocranial dysplasia: Molecular genetic analysis and phenotypic-based description of a Middle European patient group. *Am J Med Genet A* 2005; **139A**: 78-85.
13. Quack I, Vonderstrass B, Stock M, Aylsworth AS, Becker A, Brueton L *et al*. Mutation analysis of core binding factor A1 in patients with cleidocranial dysplasia. *Am J Hum Genet* 1999; **65**: 1268-1278.
14. Ott CE, Leschik G, Trotier F, Brueton L, Brunner HG, Brussel W *et al*. Deletions of the *RUNX2* gene are present in about 10% of individuals with cleidocranial dysplasia. *Hum Mutat* 2010; **31**: E1587-E1593.
15. Zhang X, Liu Y, Wang X, Sun X, Zhang C, Zheng S. Analysis of novel *RUNX2* mutations in Chinese patients with cleidocranial dysplasia. *PLoS ONE* 2017; **12**: e0181653.
16. Mastushita M, Kitoh H, Subasioglu A, Colak FK, Dundar M, Mishima K *et al*. A glutamine repeat variant of the *RUNX2* gene causes cleidocranial dysplasia. *Mol Syndromol* 2015; **6**: 50-53.
17. [Qi](https://pubmed.ncbi.nlm.nih.gov/?term=Qi+Z&cauthor_id=25119901) Z, [Yang](https://pubmed.ncbi.nlm.nih.gov/?term=Yang+W&cauthor_id=25119901) W, [Meng](https://pubmed.ncbi.nlm.nih.gov/?term=Meng+Y&cauthor_id=25119901) Y, [Liu](https://pubmed.ncbi.nlm.nih.gov/?term=Liu+Y&cauthor_id=25119901) Y. Identification of three novel frameshift mutations in the RUNX2 gene in three sporadic Chinese cases with cleidocranial dysplasia. *Zhonghua Yi Xue Yi Chuan Xue Za Zhi* 2014; **31**: 415-419.
18. Gao X, Li K, Fan Y, Sun Y, Luo X, Wang L *et al*. Identification of *RUNX2* variants associated with cleidocranial dysplasia. *Hereditas* 2019; **156**: 31.
19. Shibata A, Machida J, Yamaguchi S, Kimura M, Tatematsu T, Miyachi H *et al*. Characterisation of novel *RUNX2* mutation with alanine tract expansion from Japanese cleidocranial dysplasia patient. *Mutagenesis* 2016; **31**: 61-67.
20. Zeng L, Wei J, Zhao N, Sun S, Wang Y, Feng H. A novel 18-bp in-frame deletion mutation in *RUNX2* causes cleidocranial dysplasia. *Arch Oral Biol* 2018; **96**: 243-248.
21. Hashmi JA, Almatrafi A, Latif M, Nasir A, Basit S. An 18 bps in-frame deletion mutation in *RUNX2* gene is a population polymorphism rather than a pathogenic variant. *Eur J Med Genet* 2019; **62**: 124-128.
22. Cuellar A, Bala K, Di Pietro L, Barba M, Yagnik G, Liu JL *et al*. Gain-of-function variants and overexpression of *RUNX2* in patients with nonsyndromic midline craniosynostosis. *Bone* 2020; **137**: 115395.
23. Ryoo HM, Kang HY, Lee SK, Lee KE, Kim JW. *RUNX2* mutations in cleidocranial dysplasia patients. *Oral Dis* 2010; **16**: 55-60.
24. Napierala D, Garcia-Rojas X, Sam K, Wakui K, Chen C, Mendoza-Londono R *et al*. Mutations and promoter SNPs in *RUNX2*, a transcriptional regulator of bone formation. *Mol Genet Metab* 2005; **86**: 257-268.
25. Hansen L, Riis AK, Silahtaroglu A, Hove H, Lauridsen E, Eiberg H, Kreiborg S. *RUNX2* analysis of Danish cleidocranial dysplasia families. *Clin Genet* 2011; **79**: 254-263.
26. [Gao](https://pubmed.ncbi.nlm.nih.gov/?term=Gao+C&cauthor_id=20376792) C, [Wu](https://pubmed.ncbi.nlm.nih.gov/?term=Wu+L&cauthor_id=20376792) L, [Geng](https://pubmed.ncbi.nlm.nih.gov/?term=Geng+XJ&cauthor_id=20376792) XJ, [Song](https://pubmed.ncbi.nlm.nih.gov/?term=Song+LJ&cauthor_id=20376792) LJ, [Luo](https://pubmed.ncbi.nlm.nih.gov/?term=Luo+Q&cauthor_id=20376792) Q. Two novel *RUNX2* gene mutations in two Chinese families with cleidocranial dysplasia. *Zhonghua Yi Xue Yi Chuan Xue Za Zhi* 2010; **27**: 140-143.
27. Hamada A, Mukasa H, Taguchi Y, Akagi E, Obayashi F, Yamasaki S *et al*. Identification of a familial cleidocranial dysplasia with a novel *RUNX2* mutation and establishment of patient‑derived induced pluripotent stem cells. *Odontology* 2022; **110**: 444–451.
28. Scully A, Zhang H, Kim-Berman H, Benavides E, Hardy NC, Hu JCC. Management of Two Cases with Supernumerary Teeth. *Pediatr Dent* 2020; **42**: 58-61.
29. Suda N, Hattori M, Kosak Ki, Banshodani A, Kozai K, Tanimoto K, Moriyama K. Correlation between genotype and supernumerary tooth formation in cleidocranial dysplasia. *Orthod Craniofac Res* 2010; **13**: 197-202.
30. Hordyjewska-Kowalczyk E, Sowinska-Seidler A, Olech EM, Socha M, Glazar R, Kruczek A *et al*. Functional analysis of novel *RUNX2* mutations identified in patients with cleidocranial dysplasia. *Clin Genet* 2019; **96**: 429-438.
31. Callea M, Fattori F, Yavuz I, Bertini E. A new phenotypic variant in cleidocranial dysplasia (CCD) associated with mutation c.391C>T of the *RUNX2* gene. *BMJ Case Rep* 2012; **2012**: bcr1220115422.
32. Xu W, Chen Q, Liu C, Chen J, Xiong F, Wu B. A novel, complex *RUNX2* gene mutation causes cleidocranial dysplasia. *BMC Med Genet* 2017; **18**: 13.
33. Tang S, Xu Q, Xu X, Du J, Yang X, Jiang Y *et al*. A novel RUNX2 missense mutation predicted to disrupt DNA binding causes cleidocranial dysplasia in a large Chinese family with hyperplastic nails. *BMC Med Genet* 2007; **8**: 82.
34. Goseki-Sone M, Orimo H, Watanabe A, Hamatani R, Yokozeki M, Ohyama K *et al*. Identification of a novel frameshift mutation (383insT) in the *RUNX2* (PEBP2 á/CBFA1/AML3) gene in a Japanese patient with cleidocranial dysplasia. *J Bone Miner Metab* 2001; **19**: 263-266.
35. Çamtosun E, Akıncı A, Demiral E, Tekedereli I, Sığırcı A. A case of cleidocranial dysplasia with a novel mutation and growth velocity gain with growth hormone treatment. *J Clin Res Pediatr Endocrinol* 2019; **11**: 301-305.
36. Hebbar M, Girisha KM, Shukla A. Classical cleidocranial dysplasia in an adult, due to a novel frameshift pathogenic variant in *RUNX2*. *BMJ Case Rep* 2016; **2016**: bcr2016215162.
37. Ma D, Wang X, Guo J, Zhang J, Cai T. Identification of a novel mutation of *RUNX2* in a family with supernumerary teeth and craniofacial dysplasia by whole-exome sequencing. A case report and literature review. *Medicine (Baltimore)* 2018; **97**: e11328.
38. Xuan D, Li S, Zhang X, Lin L, Wang C, Zhang J. A novel *RUNX2* mutation in cleidocranial dysplasia patients. *Biochem Genet* 2008; **46**: 702-707.
39. Bir FD, Dinçkan N, Güven Y, Bas F, Altunoglu U, Kuvvetli SS *et al*. Cleidocranial dysplasia: Clinical, endocrinologic and molecular findings in 15 patients from 11 families. *Eur J Med Genet* 2017; **60**: 163-168.
40. Guo YW, Chiu CY, Liu CL, Jap TS, Lin LY. Novel mutation of *RUNX2* gene in a patient with cleidocranial dysplasia. *Int J Clin Exp Pathol* 2015; **8**: 1057-1062.
41. Mundlos S, Otto F, Mundlos C, Mulliken JB, Aylsworth AS, Albright S *et al*. Mutations involving the transcription factor CBFA1 cause cleidocranial dysplasia. *Cell* 1997; **89**: 773-779.
42. Carbonare LD, Minoia A, Gandini A, Piritore FC, Patuzzo C, Ceretti L *et al*. Unraveling *RUNX2* mutation in a cleidocranial dysplasia patient: Molecular insights into osteogenesis and proteostasis. *Genes Dis* 2025; **12**: 101449.
43. Morava E, Karteszi J, Weisenbach J, Caliebe A, Mundlos S, Mehes K. Cleidocranial dysplasia with decreased bone density and biochemical findings of hypophosphatasia. *Eur J Pediatr* 2002; 161: 619-622.
44. Zhang C, Zhen S, Wang Y, Zhao Y, Zhu J, Ge L. Mutational analysis of *RUNX2* gene in Chinese patients with cleidocranial dysplasia. *Mutagenesis* 2010; **25**: 589-594.
45. Lee B, Thirunavukkarasu K, Zhou L, Pastore L, Baldini A, Hecht J *et al*. Missense mutations abolishing DNA binding of the osteoblast-specific transcription factor *OSF2/CBFA1* in cleidocranial dysplasia. *Nat Genet* 1997; **16**: 307-310.
46. Jung YJ, Bae HS, Ryoo HM, Baek SH. A novel *RUNX2* mutation in exon 8, G462X, in a patient with Cleidocranial Dysplasia. *J Cell Biochem* 2018; **119**: 1152-1162.
47. Hordyjewska E, Jaruga A, Kandzierski G, Tylzanowski P. Novel mutation of the *RUNX2* gene in patients with cleidocranial dysplasia. *Mol Syndromol* 2017; **8**: 253-260.
48. Fang CY, Xue JJ, Tan L, Jiang CH, Gao QP, Liang DS, Wu LQ. A novel single-base deletion mutation of the *RUNX2* gene in a Chinese family with cleidocranial dysplasia. *Genet Mol Res* 2011; **10**: 3539-3544.
49. Giannotti A, Tessa A, Patrono C, De Florio L, Velardo M, Dionisi-Vici C *et al*. A novel CBFA1 mutation (R190W) in an Italian family with cleidocranial dysplasia. *Hum Mutat* 2000; **16**: 277.
50. Bergwitz C, Prochnau A, Mayr B, Kramer FJ, Rittieodt M, Berten HL *et al*. Identification of novel *CBFA1/RUNX2* mutations causing cleidocranial dysplasia. *J Inherit Metab Dis* 2001; **24**: 648-656.
51. [Qiu](https://pubmed.ncbi.nlm.nih.gov/?term=Qiu+ZQ&cauthor_id=16221346) ZQ, [Tang](https://pubmed.ncbi.nlm.nih.gov/?term=Tang+AL&cauthor_id=16221346) AL, [Yu](https://pubmed.ncbi.nlm.nih.gov/?term=Yu+W&cauthor_id=16221346) W, [Ao](https://pubmed.ncbi.nlm.nih.gov/?term=Ao+Y&cauthor_id=16221346) Y, [Wilson](https://pubmed.ncbi.nlm.nih.gov/?term=Wilson+HY&cauthor_id=16221346) HYL, [Wei](https://pubmed.ncbi.nlm.nih.gov/?term=Wei+M&cauthor_id=16221346) M, [Zhang](https://pubmed.ncbi.nlm.nih.gov/?term=Zhang+X&cauthor_id=16221346) X. A Chinese girl with cleidocranial dysplasia (CCD) caused by the recurrent R190W mutation in *RUNX 2*. *Zhonghua Er Ke Za Zhi* 2004; **42**: 759-761.
52. Kisiel BM, Kostrzewa G, Wlasienko P, Kruczek A, Gajdulewicz M, Maciejak D *et al*. Cleidocranial dysplasia in a Polish population: high frequency of the R193X mutation. *Clin Genet* 2006; **70**: 167-169.
53. Lin WD, Lin SP, Wang CH, Tsai Y, Chen CP, Tsai FJ. *RUNX2* mutations in Taiwanese patients with cleidocranial dysplasia. *Genet Mol Biol* 2011; **34**: 201-204.
54. Wang J, Huang X, Lai C, Jiang K. Clinical spectrum of cleidocranial dysplasia in a family with twins. *Pediatr Int* 2013; **55**: 392-395.
55. [Jiang](https://pubmed.ncbi.nlm.nih.gov/?term=Jiang+T&cauthor_id=24298808) T, [Jiang](https://pubmed.ncbi.nlm.nih.gov/?term=Jiang+X&cauthor_id=24298808) X, [Zhang](https://pubmed.ncbi.nlm.nih.gov/?term=Zhang+Y&cauthor_id=24298808) Y. Mutation analysis of the *RUNX2* gene in a family with cleidocranial dysplasia. *Hua Xi Kou Qiang Yi Xue Za Zhi* 2013; **31**: 522-525.
56. Xue R, Zhang G, Chen X, Ye X. Cleidocranial dysplasia causing respiratory distress in neonates: A case report and literature review. *Front Genet* 2021; **12**: 696685.
57. Zhao S, Wang T, Yang H, Huang R. Neonatal familiar cleidocranial dysplasia: A case report. *Am J Case Rep* 2025; **26**: e946322.
58. [Golan](https://pubmed.ncbi.nlm.nih.gov/?term=Golan+I&cauthor_id=15301373) I, [Baumert](https://pubmed.ncbi.nlm.nih.gov/?term=Baumert+U&cauthor_id=15301373) U, [Pragier](https://pubmed.ncbi.nlm.nih.gov/?term=Pragier+R&cauthor_id=15301373) R, [Aknin](https://pubmed.ncbi.nlm.nih.gov/?term=Aknin+JJ&cauthor_id=15301373) JJ, [Rodde](https://pubmed.ncbi.nlm.nih.gov/?term=Rodde+J&cauthor_id=15301373) J, [Müssig](https://pubmed.ncbi.nlm.nih.gov/?term=M%C3%BCssig+D&cauthor_id=15301373) D. Inter- and intrafamilial expression of cleidocranial dysostosis. *Orthod Fr* 2003; **74**: 7-13.
59. [Schnerch](https://pubmed.ncbi.nlm.nih.gov/?term=Schnerch+D&cauthor_id=24138303) D, [Lausch](https://pubmed.ncbi.nlm.nih.gov/?term=Lausch+E&cauthor_id=24138303) E, [Becker](https://pubmed.ncbi.nlm.nih.gov/?term=Becker+H&cauthor_id=24138303) H, [Felthaus](https://pubmed.ncbi.nlm.nih.gov/?term=Felthaus+J&cauthor_id=24138303) J, [Pfeifer](https://pubmed.ncbi.nlm.nih.gov/?term=Pfeifer+D&cauthor_id=24138303) D, [Mundlos](https://pubmed.ncbi.nlm.nih.gov/?term=Mundlos+S&cauthor_id=24138303) S *et al*. Up-regulation of *RUNX2* in acute myeloid leukemia in a patient with an inherent *RUNX2* haploinsufficiency and cleidocranial dysplasia. *Leuk Lymphoma* 2014; **55**: 1930-1932.
60. Puppin C, Pellizzari L, Fabbro D, Fogolari F, Tell G, Tessa A *et al*. Functional analysis of a novel *RUNX2* missense mutation found in a family with cleidocranial dysplasia. *J Hum Genet* 2005; **50**: 679-683.
61. [Wang](https://pubmed.ncbi.nlm.nih.gov/?term=Wang+GX&cauthor_id=21215027) GX, [Ma](https://pubmed.ncbi.nlm.nih.gov/?term=Ma+LX&cauthor_id=21215027) LX, [Xu](https://pubmed.ncbi.nlm.nih.gov/?term=Xu+WF&cauthor_id=21215027) WF, [Song](https://pubmed.ncbi.nlm.nih.gov/?term=Song+FL&cauthor_id=21215027) FL, [Sun](https://pubmed.ncbi.nlm.nih.gov/?term=Sun+RP&cauthor_id=21215027) RP. Clinical and image features, and identification of pathogenic gene mutation of two cleidocranial dysplasia families. *Zhonghua Er Ke Za Zhi* 2010; **48**: 834-8.
62. Li Y, Pan W, Xu W, He N, Chen X, Liu H *et al*. *RUNX2* mutations in Chinese patients with cleidocranial dysplasia. *Mutagenesis* 2009; **24**: 425-431.
63. Cao Y, Cheng YKY, Leung TY, Xue S, Zheng Y, Choy KW *et al*. Early prenatal detection of autosomal dominant skeletal dysplasia using first-trimester ultrasound and cell-free fetal DNA screening: three case reports. *Hong Kong Med J* 2026; **32**: 58-61.
64. Zeng L, Wei J, Han D, Liu H, Liu Y, Zhao N *et al*. Functional analysis of novel *RUNX2* mutations in cleidocranial dysplasia. *Mutagenesis* 2017; **32**: 437-443.
65. Lu H, Zeng B, Yu D, Jing X, Hu B, Zhao W, Wang Y. Complex dental anomalies in a belatedly diagnosed cleidocranial dysplasia patient. *Imaging Sci Dent* 2015; **45**: 187-192.
66. Kamil G, Yoon JY, Yoo S, Cheon CK. Clinical relevance of targeted exome sequencing in patients with rare syndromic short stature. *Orphanet J Rare Dis* 2021; **16**: 297.
67. [Jiao](https://pubmed.ncbi.nlm.nih.gov/?term=Jiao+Z&cauthor_id=31813142) Z, [Chen](https://pubmed.ncbi.nlm.nih.gov/?term=Chen+Y&cauthor_id=31813142) Y, [Zhao](https://pubmed.ncbi.nlm.nih.gov/?term=Zhao+Z&cauthor_id=31813142) Z, [Wu](https://pubmed.ncbi.nlm.nih.gov/?term=Wu+Q&cauthor_id=31813142) Q, [Ren](https://pubmed.ncbi.nlm.nih.gov/?term=Ren+S&cauthor_id=31813142) S, [Kong](https://pubmed.ncbi.nlm.nih.gov/?term=Kong+X&cauthor_id=31813142) X. Prenatal diagnosis for two families affected with cleidocranial dysplasia due to novel *RUNX2* variants. *Zhonghua Yi Xue Yi Chuan Xue Za Zhi* 2019; **36**: 1179-1182.
68. Marchisella C, Rolando F, Muscarella LA, Zelante L, Bracco P, Piemontese MR. Identification of a novel *RUNX2* gene mutation in an Italian family with cleidocranial dysplasia. *Eur J Orthod* 2011; **33**: 498-502.
69. Zhang T, Wu J, Zhao X, Hou F, Ma T, Wang H *et al*. Whole-exome sequencing identification of a novel splicing mutation of *RUNX2* in a Chinese family with cleidocranial dysplasia. *Arch Oral Biol* 2019; **100**: 49-56.
70. Matsushita M, Kitoh H, Kaneko H, Mishima K, Itoh Y, Tokita Y, Ishiguro N. A novel in-frame deletion of the *RUNX2* gene causes a classic form of cleidocranial dysplasia. *J Bone Miner Metab* 2014; **32**: 96-99.
71. Bufalino A, Paranaıba LMR, Gouvea AF, Gueiros LA, Martelli-Junior H, Junior JJ *et al*. Cleidocranial dysplasia: oral features and genetic analysis of 11 patients. *Oral Dis* 2012; **18**: 184-190.
72. [Zhang](https://pubmed.ncbi.nlm.nih.gov/?term=Zhang+CY&cauthor_id=19767586) CY, [Zheng](https://pubmed.ncbi.nlm.nih.gov/?term=Zheng+SG&cauthor_id=19767586) SG, [Wang](https://pubmed.ncbi.nlm.nih.gov/?term=Wang+YX&cauthor_id=19767586) YX, [Zhu](https://pubmed.ncbi.nlm.nih.gov/?term=Zhu+JX&cauthor_id=19767586) JX, [Zhu](https://pubmed.ncbi.nlm.nih.gov/?term=Zhu+X&cauthor_id=19767586) X, [Zhao](https://pubmed.ncbi.nlm.nih.gov/?term=Zhao+YM&cauthor_id=19767586) YM, [Ge](https://pubmed.ncbi.nlm.nih.gov/?term=Ge+LH&cauthor_id=19767586) LH. Novel *RUNX2* mutations in Chinese individuals with cleidocranial dysplasia. *J Dent Res* 2009; **88**: 861-866.
73. Yamachika E, Tsujigiwa H, Ishiwari Y, Mizukawa N, Nagai N, Sugahara T. Identification of a stop codon mutation in the CBFA1 *runt* domain from a patient with cleidocranial dysplasia and cleft lip. *J Oral Pathol Med* 2001; **30**: 381-383.
74. Suda N, Hamada T, Hattori M, Torii C, Kosaki K, Moriyama K. Diversity of supernumerary tooth formation in siblings with cleidocranial dysplasia having identical mutation in *RUNX2*: possible involvement of non-genetic or epigenetic regulation. *Orthod Craniofacial Res* 2007; **10**: 222-225.
75. Ge J, Guo S, Fu Y, Zhou P, Zhang P, Du Y *et al*. Dental follicle cells participate in tooth eruption via the RUNX2-MiR-31-SATB2 loop. *J Dent Res* 2015; **94**: 936-944.
76. Chen T, Hou J, Hu LL, Gao J, Wu BL. A novel small deletion mutation in *RUNX2* gene in one Chinese family with cleidocranial dysplasia. *Int J Clin Exp Pathol* 2014; **7**: 2490-2495.
77. López JT, Martinez SG, del Refugio Rivera Vega M, Hernández-Zamora E, Covarrubias SC, Castrejón BAI, Huerta LMG. New genetic variants of *RUNX2* in Mexican families cause cleidocranial dysplasia. *Biology (Basel)* 2024; **13**: 173.
78. Sakai N, Hasegawa H, Yamazaki Y, Ui K, Tokunaga K, Hirose R *et al*. A case of a Japanese patient with cleidocranial dysplasia possessing a mutation of *CBFA1* gene. *J Craniofac Surg* 2002; **13**: 31-34.
79. Xuan D, Li S, Zhang X, Hu F, Lin L, Wang C, Zhang J. Mutations in the *RUNX2* gene in Chinese patients with cleidocranial dysplasia. *Ann Clin Lab Sci* 2008; **38**: 15-24.
80. Thaweesapphithak S, Saengsin J, Kamolvisit W, Theerapanon T, Porntaveetus T, Shotelersuk V. Cleidocranial dysplasia and novel *RUNX2* variants: dental, craniofacial, and osseous manifestations. *J Appl Oral Sci* 2022; **30**: e20220028.
81. [Lee](https://pubmed.ncbi.nlm.nih.gov/?term=Lee+KE&cauthor_id=24222232) KE, [Seymen](https://pubmed.ncbi.nlm.nih.gov/?term=Seymen+F&cauthor_id=24222232) F, [Ko](https://pubmed.ncbi.nlm.nih.gov/?term=Ko+J&cauthor_id=24222232) J, [Yildirim](https://pubmed.ncbi.nlm.nih.gov/?term=Yildirim+M&cauthor_id=24222232) M, [Tuna](https://pubmed.ncbi.nlm.nih.gov/?term=Tuna+EB&cauthor_id=24222232) EB, [Gencay](https://pubmed.ncbi.nlm.nih.gov/?term=Gencay+K&cauthor_id=24222232) K, [Kim](https://pubmed.ncbi.nlm.nih.gov/?term=Kim+JW&cauthor_id=24222232) JW. *RUNX2* mutations in cleidocranial dysplasia. *Genet Mol Res* 2013; **12**: 4567-4574.
82. Wu LZ, Su WO, Liu YF, Ge X, Zhang Y, Wang XJ. Role of the *RUNX2* p.R225Q mutation in cleidocranial dysplasia: a rare presentation and an analysis of the RUNX2 protein structure. *Genet Mol Res* 2014; **13**: 1187-1194.
83. Hsueh SJ, Lee NC, Yang SH, Lin HI, Lin CH. A limb-girdle myopathy phenotype of *RUNX2* mutation in a patient with cleidocranial dysplasia: a case study and literature review. *BMC Neurol* 2017; **17**: 2.
84. Medina O, Munoz N, Moneriz C. Cleidocranial dysplasia: a case report. *Rev Chil Pediatr* 2017; **88**: 517-523.
85. Callea M, Fattori F, Bertini ES, Yavuz I, Bellacchio E, Avendaño A *et al*. Clinical and molecular study in a family with cleidocranial dysplasia. *Arch Argent Pediatr* 2017; **115**: e440-e444.
86. Liu Y, Sun X, Zhang X, Wang X, Zhang C, Zheng S. *RUNX2* mutation impairs osteogenic differentiation of dental follicle cells. *Arch Oral Biol* 2019; **97**: 156-164.
87. Kim JH, Kim SJ, Kim HH, Kim JK. Sporadic cleidocranial dysplasia in a newborn: A 4-year follow-up study. *Neonatal Med* 2020; **27**: 192-196.
88. Yigin AK, Duz MB, Seven M. Rare Findings in cleidocranial dysplasia caused by RUNX mutation. *Glob Med Genet* 2021; **9**: 23-28.
89. Inchingolo AD, Patano A, Coloccia G, Ceci S, Angelo Inchingolo AM, Marinelli G *et al*. Genetic pattern, orthodontic and surgical management of multiple supplementary impacted teeth in a rare, cleidocranial dysplasia patient: A case report. *Medicina (Kaunas)* 2021; **57**: 1350.
90. Zhao W, An Y, Zhang X, Zhuo N, Cai C, Arigong G. A Chinese premature infant with cleidocranial dysplasia characterized by heterozygous *RUNX2* mutation and cerebral infarction: a case report. *Front Pediatr* 2025; **13**: 1643266.
91. Wang F, Dai PF, Gao WJ. Prenatal ultrasonography and genetic analysis of fetal cleidocranial dysplasia: A case report. *World J Clin Cases* 2025; **13**: 97584.
92. Wang J, Li Q, Li H, Liu X, Hu Y, Bai Y, Yang K. A novel *RUNX2* splice site mutation in Chinese associated with cleidocranial dysplasia. *Heliyon* 2024; **10**: e40277.
93. Kamamoto M, Machida J, Miyachi H, Ono T, Nakayama A, Shimozato K, Tokita Y. A novel mutation in the C-terminal region of RUNX2/CBFA1 distal to the DNA-binding runt domain in a Japanese patient with cleidocranial dysplasia. *Int J Oral Maxillofac Surg* 2011; **40**: 434-449.
94. Takaki N, Mori J, Matsuo S, Osamura T, Michigami T. Cleidocranial dysplasia with growth hormone deficiency: a case report. *BMC Pediatr* 2020; **20**: 19.
95. Daneshjoo O, Ebrahimi P, Salehi LB, Pizzuti A, Garshasbi M. Identification of a novel *RUNX2* gene mutation and early diagnosis of CCD in a cleidocranial dysplasia suspected Iranian family. *Clin Case Rep* 2020; **8**: 2333-2340.
96. Jamali E, Khalesi R, Bitarafan F, Almadani N, Garshasbi M. Identification of a novel splice site mutation in *RUNX2* gene in a family with rare autosomal dominant cleidocranial dysplasia. *Iran Biomed J* 2021; **25**: 297-302.
97. Cunningham ML, Seto ML, Hing AV, Bull MJ, Hopkin RJ, Leppig KA. Cleidocranial dysplasia with severe parietal bone dysplasia: C-terminal *RUNX2* mutations. *Birth Defects Res A Clin Mol Teratol* 2006; **76**: 78-85.
98. Pal T, Napierala D, Becker TA, Loscalzo M, Baldridge D, Lee B, Sutphen R. The presence of germ line mosaicism in cleidocranial dysplasia. *Clin Genet* 2007; **71**: 589-591.
99. Wang S, Zhang S, Wang Y, Chen Y, Zhou L. Cleidocranial dysplasia syndrome: clinical characteristics and mutation study of a Chinese family. *Int J Clin Exp Med* 2013; **6**: 900-907.
100. Peng YJ, Chen QY, Fu DJ, Liu ZM, Mao TT, Li J, She WT. A novel gene mutation of Runx2 in cleidocranial dysplasia. *J Huazhong Univ Sci Technol* 2017; **37**: 772-776.
101. Carbonare LD, Antoniazzi F, Gandini A, Orsi S, Bertacco J, Vigni VL *et al*. Two novel C-terminus *RUNX2* mutations in two cleidocranial dysplasia (CCD) patients impairing p53 expression. *Int J Mol Sci* 2021; **22**: 10336.
102. Anthonappa RP, Yan-Hui F, King NM, Rabie ABM, You-Qiang S. Novel complex disease allele mutations in cleidocranial dysplasia patients. *J Oral Pathol Med* 2014; **43**: 798-800.
103. Ma Y, Zhao F, Yu F. Cleidocranial dysplasia syndrome with epilepsy: a case report. *BMC Pediatr* 2019; **19**: 97.
104. Zhang Z, Li K, Yan M, Lin Q, Lv J, Zhu P, Xu Y. Metabolomics profiling of cleidocranial dysplasia. *Clin Oral Investig* 2019; **23**: 1031-1040.
105. Arikan Y, Onat T. Custom exome panel revealed new mutations in *MAPK14* and novel mutation in *RUNX2* gene in patients with PCOS. *Sci Rep* 2025; **15**: 2128.
106. Xuan DY, Sun X, Yan YX, Xie BY, Xu PP, Zhang JC. Effect of cleidocranial dysplasia-related novel mutation of *RUNX2* on characteristics of dental pulp cells and tooth development. *J Cell Biochem* 2010; **111**: 1473-1481.
107. Qin XY, Jia PZ, Zhao HX, Li WR, Chen F, Lin JX. Novel mutation of cleidocranial dysplasia‑related frameshift runt‑related transcription factor 2 in a sporadic Chinese case. *Chi Med J (Engl)* 2017; **130**: 165-170.
108. Ding B, Li C, Xuan K, Liu N, Tang L, Liu Y *et al*. The effect of the cleidocranial dysplasia-related novel 1116_1119insC mutation in the *RUNX2* gene on the biological function of mesenchymal cells. *Eur J Med Genet* 2013; **56**: 180-187.
109. Tsai FJ, Wu JY, Lin WD, Tsai CH. A stop codon mutation in the *CBFA1* gene causes cleidocranial dysplasia. *Acta Pñdiatr* 2000; **89**: 1262-1269.
110. Muzio LL, Tetè S, Mastrangelo F, Cazzolla AP, Lacaita MG, Margaglione M, Campisi G. A novel mutation of gene CBFA1/RUNX2 in cleidocranial dysplasia. *Ann Clin Lab Sci* 2007; **37**: 115-120.
111. Zheng Q, Sebald E, Zhou G, Chen Y, Wilcox W, Lee B, Krakow D. Dysregulation of chondrogenesis in human cleidocranial dysplasia. *Am J Hum Genet* 2005; **77**: 305-312.
112. Wang GX, Sun RP, Song FL. A novel *RUNX2* mutation (T420I) in Chinese patients with cleidocranial dysplasia. *Genet Mol Res* 2010; **9**: 41-47.
113. Lee C, Jung HS, Baek JA, Leem DH, Ko SO. Manifestation and treatment in a cleidocranial dysplasia patient with a RUNX2 (T420I) mutation. *Maxillofac Plast Reconstr Surg* 2015; **37**: 41.
114. [Le Caignec](https://pubmed.ncbi.nlm.nih.gov/?term=Le+Caignec+C&cauthor_id=16865744) C, [Winer](https://pubmed.ncbi.nlm.nih.gov/?term=Winer+N&cauthor_id=16865744) N, [Philippe](https://pubmed.ncbi.nlm.nih.gov/?term=Philippe+HJ&cauthor_id=16865744) HJ, [Rival](https://pubmed.ncbi.nlm.nih.gov/?term=Rival+JM&cauthor_id=16865744) JM. Two independent rare events—a mutation in the *RUNX2* gene and a *de novo* balanced translocation—in a fetus with cleidocranial dysplasia: pitfall in prenatal diagnosis. *Prenat Diagn* 2006; **26**: 759-760.
115. Sekerci AE, Balta B, Bahadir O, Sisman Y, Dundar M, Tokmak TT, Mundlos S. Cleidocranial dysplasia with a rare mutation: Study of a family with review of literature. *Open J Stomatol* 2013; **3**: 402-410.
116. Gong L, Odilov B, Han F, Liu F, Sun Y, Zhang N *et al*. Identification a novel de novo *RUNX2* frameshift mutation associated with cleidocranial dysplasia. *Genes Genomics* 2022; **44**: 683-690.
117. Zhao W, Ye G, Yu X, Xu J, Li Z. Identification of a novel mutation in the runt-related transcription factor 2 gene in a Chinese family with cleidocranial dysplasia. *J Craniofac Surg* 2021; **32**: e541-e544.
118. Franceschi R, Maines E, Fedrizzi M, Piemontese MR, De Bonis P, Agarwal N *et al*. Familial cleidocranial dysplasia misdiagnosed as rickets over three generations. *Pediatr Int* 2015; **57**: 1003-1006.
119. Bedeschi MF, Bonarrigo F, Manzoni F, Milani D, Piemontese MR, Guez S, Esposito S. Ehlers-Danlos syndrome versus cleidocranial dysplasia. *Ital J Pediatr* 2014; **40**: 49.
120. Lee MTM, Tsai ACH, Chou CH, Sun FM, Huang LC, Yen P *et al*. Intragenic microdeletion of RUNX2 is a novel mechanism for cleidocranial dysplasia. *Genomic Med* 2008; **2**: 45-49.
121. El-Gharbawy AH, Peeden JN, Lachman RS, Graham JM, Moore SR, Rimoin DL. Severe cleidocranial dysplasia and hypophosphatasia in a child with microdeletion of the C-terminal region of RUNX2. *Am J Med Genet A* 2010; **152A**: 169-174.
122. Zhang Y, Duan X. A Novel 90-kbp Deletion of RUNX2 Associated with cleidocranial dysplasia. *Genes* 2022; **13**: 1128.
123. Qian Y, Zhang Y, Wei B, Zhang M, Yang J, Leng C *et al*. A novel Alu-mediated microdeletion in the *RUNX2* gene in a Chinese patient with cleidocranial dysplasia. *J Genet* 2018; **97**: 137-143.
124. Franceschi R, Stringari G, Soli F, Pedrolli A, Maines E. Newborn with cleidocranial dysplasia. Skeletal Radiol 2022; **51**: 2351-2532.
125. Li ZJ, Wang JY, Gao MF, Wu DL, Chang X. Orthodontic treatment of a patient with cleidocranial dysplasia: A case report. *Exp Ther Med* 2016; **12**: 690-694.
126. Pamuk ON, Mundlos S, Cakir N. Cleidocranial dysplasia in a mother and her two children. *Joint Bone Spine* 2008; **75**: 725-727.
127. [Cavalli](https://pubmed.ncbi.nlm.nih.gov/?term=Cavalli+P&cauthor_id=14634985) P, [Santorelli](https://pubmed.ncbi.nlm.nih.gov/?term=Santorelli+FM&cauthor_id=14634985) FM, [Bontardelli](https://pubmed.ncbi.nlm.nih.gov/?term=Bontardelli+M&cauthor_id=14634985) M, [Tessa](https://pubmed.ncbi.nlm.nih.gov/?term=Tessa+A&cauthor_id=14634985) A, [Bosi](https://pubmed.ncbi.nlm.nih.gov/?term=Bosi+A&cauthor_id=14634985) A, [Poggiani](https://pubmed.ncbi.nlm.nih.gov/?term=Poggiani+C&cauthor_id=14634985) C. Prenatal exclusion of cleidocranial dysplasia. 2003; *Prenat Diagn* **23**: 945-946.
128. Goto H, Machida J, Shibata A, Tatematsu T, Osumi E, Miyachi H, *et al*. Novel RUNX2/CBFA1 mutation in the runt domain in a Japanese patient with cleidocranial dysplasia. *Int J Oral Maxillofac Surg* 2017; **29**: 222-224.
129. Zhang P, He P, Xu P, Liao L. Cleidocranial dysplasia: a case report and gene mutation analysis. *Hua Xi Kou Qiang Yi Xue Za Zhi* 2022; **40**: 360-364.
