# Supplementary material for: Cleidocranial dysplasia caused by a novel de novo RUNX2 splice-site variant
Source: Hum Genome Var. 2026 Jun 4;13:19. doi: 10.1038/s41439-026-00353-3 (PMC13478401; doi:10.1038/s41439-026-00353-3)
Supplement: Supplementary file 2 [file 41439_2026_353_MOESM2_ESM.docx]

Supplementary Data 2. *RUNX2* variants and associated CCD features

| Patient No. | Gender | | | | | | Age (Years) | Ethnicity | Nucleic acid  (NM_001024630.4) | Amino acid  (NP_001019801.3) | ClinVar　(accessed Feb. 2026) | Inheritance | Short stature | Head1 | Head2 | Face | Ears | Eyes | Nose | Mouse | Teeth | Chest | Skeletal | Finger | Neurologic | References |
| --- | --- | --- | --- | --- | --- | --- | --- | --- | --- | --- | --- | --- | --- | --- | --- | --- | --- | --- | --- | --- | --- | --- | --- | --- | --- | --- |
| 1 | Female | | | | | | 15 | Chinese | c.2T>C | p.Met1? | NR | N/A | N | N/A | Y | Y | N/A | Y | N/A | Y | Y | Y | N | N/A | N/A | 1 |
| 2 | Male | | | | | | 18 | Turkish | c.29dupT | p.Thr11Aspfs*150 | Pathogenic | *De novo* | N/A | Y | Y | Y | N/A | Y | N/A | N | Y | Y | Y | N | N/A | 2 |
| 3 | Female | | | | | | 50 | Turkish | c.29dupT | p.Thr11Aspfs*150 |  | *De novo* | N/A | N/A | N | N | N/A | N | N/A | N | Y | N | N | N | N/A | 2 |
| 4 | Female | | | | | | 28 | Korean | c.58+1G>A | - | NR | *De novo* | N/A | N/A | N/A | N/A | N/A | N/A | N/A | N/A | Y | N/A | N/A | N/A | N/A | 3 |
| 5 | N/A | | | | | | N/A | Italian | c.58+2T>A | - | NR | N/A | N/A | Y | N/A | N/A | Y | N/A | N/A | N/A | N/A | Y | N/A | N/A | N/A | 4 |
| 6 | Male | | | | | | N/A | Japanese | c.59-1G>T | - | NR | Maternal | N/A | N/A | N/A | N/A | N/A | N/A | N/A | N/A | N/A | N/A | N/A | N/A | N/A | 5 |
| 7 | Female | | | | | | 15 | Turkish | c.90dupC | p.Ser31Leufs*130 | Pathogenic | *De novo* | N/A | Y | N | Y | N/A | N | N/A | Y | Y | N/A | N | Y | N/A | 2 |
| 8 | Male | | | | | | 20 | Chinese | c.90dupC | p.Ser31Leufs*130 |  | N/A | Y | N/A | N/A | N/A | N/A | N/A | N/A | Y | Y | N | N/A | N/A | N/A | 6 |
| 9 | Male | | | | | | N/A | N/A | c.90dupC | p.Ser31Leufs*130 |  | Paternal | N/A | N | N/A | N/A | N/A | N/A | N/A | N/A | Y | N | N/A | N/A | N/A | 7 |
| 10 | Female | | | | | | N/A | N/A | c.90dupC | p.Ser31Leufs*130 |  | N/A | N/A | Y | N/A | N/A | N/A | N/A | N/A | N/A | Y | N | N/A | N/A | N/A | 7 |
| 11 | Male | | | | | | N/A | Chinese | c.132delG | p.Val45Trpfs*99 | NR | *De novo* | Y | N/A | N/A | N/A | N/A | N/A | N/A | N/A | Y | N | N | N/A | N/A | 8 |
| 12 | N/A | | | | | | N/A | Japanese | c.133_134insGTCC | p.Val45Glyfs*117 | NR | N/A | N/A | Y | N/A | N/A | N/A | N/A | N/A | N/A | N/A | Y | N/A | N/A | N/A | 9 |
| 13 | N/A | | | | | | N/A | Japanese | c.133_134insGTCC | p.Val45Glyfs*117 |  | N/A | N/A | Y | N/A | N/A | N/A | N/A | N/A | N/A | N/A | Y | N/A | N/A | N/A | 9 |
| 14 | N/A | | | | | | N/A | Japanese | c.133_134insGTCC | p.Val45Glyfs*117 |  | N/A | N/A | Y | N/A | N/A | N/A | N/A | N/A | N/A | N/A | Y | N/A | N/A | N/A | 9 |
| 15 | N/A | | | | | | N/A | Japanese | c.133_134insGTCC | p.Val45Glyfs*117 |  | N/A | N/A | Y | N/A | N/A | N/A | N/A | N/A | N/A | N/A | Y | N/A | N/A | N/A | 9 |
| 16 | N/A | | | | | | N/A | Japanese | c.133_134insGTCC | p.Val45Glyfs*117 |  | N/A | N/A | Y | N/A | N/A | N/A | N/A | N/A | N/A | N/A | Y | N/A | N/A | N/A | 9 |
| 17 | Female | | | | | | 15 | Korean | c.148C>T | p.Gln50* | Pathogenic | Familial | N/A | N/A | N/A | N/A | N/A | N/A | N/A | N/A | Y | N/A | N/A | N/A | N/A | 3 |
| 18 | Female | | | | | | 4 | Mexican | c.158A>T | p.Gln53Leu | NR | *De novo* | N | Y | Y | N/A | Y | N/A | N/A | Y | Y | Y | N/A | N/A | N/A | 10 |
| 19 | Male | | | | | | 11 | N/A | c.166C>T | p.Gln56* | NR | Maternal | N | Y | Y | N/A | N/A | N/A | N/A | N/A | Y | N | Y | N/A | N/A | 11 |
| 20 | Male | | | | | | 23 | Caucasian | c.169C>T | p.Gln57* | NR | Familial | N/A | N/A | Y | Y | N/A | N/A | N/A | N/A | N | N/A | N/A | N/A | N/A | 12 |
| 21 | Male | | | | | | 50 | Caucasian | c.169C>T | p.Gln57* |  | Familial | N/A | N/A | Y | N | N/A | N/A | N/A | N/A | N | N/A | N/A | N/A | N/A | 12 |
| 22 | N/A | | | | | | N/A | Japanese | c.178delC | p.Gln60Serfs*84 | NR | N/A | N/A | Y | N/A | N/A | N/A | N/A | N/A | N/A | Y | Y | N/A | N/A | N/A | 9 |
| 23 | N/A | | | | | | N/A | Japanese | c.178delC | p.Gln60Serfs*84 |  | N/A | N/A | Y | N/A | N/A | N/A | N/A | N/A | N/A | Y | Y | N/A | N/A | N/A | 9 |
| 24 | N/A | | | | | | N/A | N/A | c.186_201dup | p.Gln68Alafs*98 | NR | N/A | N/A | N/A | N/A | N/A | N/A | N/A | N/A | N/A | N/A | N/A | N/A | N/A | N/A | 13 |
| 25 | N/A | | | | | | N/A | N/A | c.186_201dup | p.Gln68Alafs*98 |  | N/A | N/A | N/A | N/A | N/A | N/A | N/A | N/A | N/A | N/A | N/A | N/A | N/A | N/A | 13 |
| 26 | N/A | | | | | | N/A | N/A | c.193C>T | p.Gln65* | NR | N/A | N/A | N/A | N/A | N/A | N/A | N/A | N/A | N/A | N/A | N/A | N/A | N/A | N/A | 14 |
| 27 | N/A | | | | | | N/A | Japanese | c.196C>T | p.Gln66* | Pathogenic | N/A | N/A | N/A | N/A | N/A | N/A | N/A | N/A | N/A | Y | Y | N/A | N/A | N/A | 9 |
| 28 | N/A | | | | | | N/A | Japanese | c.196C>T | p.Gln66* |  | N/A | N/A | N/A | N/A | N/A | N/A | N/A | N/A | N/A | Y | Y | N/A | N/A | N/A | 9 |
| 29 | N/A | | | | | | N/A | Japanese | c.196C>T | p.Gln66* |  | N/A | N/A | N/A | N/A | N/A | N/A | N/A | N/A | N/A | Y | Y | N/A | N/A | N/A | 9 |
| 30 | Female | | | | | | 14 | Chinese | c.199C>T | p.Gln67* | Pathogenic | Maternal | N | Y | N/A | Y | N/A | N/A | N/A | Y | Y | Y | N/A | N/A | N/A | 15 |
| 31 | Female | | | | | | N/A | Chinese | c.199C>T | p.Gln67* |  | *De novo* | N | N/A | N/A | N/A | N/A | N/A | N/A | N/A | Y | N/A | N/A | N/A | N/A | 15 |
| 32 | Male | | | | | | 37 | Turkish | c.203delAinsCG | p.Gln68Profs*93 | Likely pathogenic | Familial | Y | N/A | Y | Y | N/A | Y | N/A | N/A | Y | Y | Y | N | N/A | 2 |
| 33 | Male | | | | | | 3M | Turkish | c.203delAinsCG | p.Gln68Profs*93 |  | Familial | N/A | Y | Y | Y | N/A | Y | N/A | N | Y | Y | Y | N | N/A | 2 |
| 34 | N/A | | | | | | N/A | N/A | c.207_208ins13 | p.Glu72Glnfs*93 | NR | N/A | N/A | N/A | N/A | N/A | N/A | N/A | N/A | N/A | N/A | N/A | N/A | N/A | N/A | 14 |
| 35 | Male | | | | | | 2 | Turkey | c.213_214insCAGCAGCAGCAG | p.Gln71_Glu72insGlnGlnGlnGln | NR | Familial | N/A | Y | Y | Y | N/A | N/A | N/A | Y | Y | Y | N/A | N/A | N/A | 16 |
| 36 | Male | | | | | | 27 | Turkey | c.213_214insCAGCAGCAGCAG | p.Gln71_Glu72insGlnGlnGlnGln |  | N/A | N/A | Y | Y | Y | N/A | N/A | N/A | Y | N/A | Y | N/A | N/A | N/A | 16 |
| 37 | N/A | | | | | | N/A | N/A | c.220_342del | p.Ala74_Val114del | NR | *De novo* | N/A | Y | N/A | N/A | N/A | N/A | N/A | N/A | N/A | Y | N/A | N/A | N/A | 7 |
| 38 | N/A | | | | | | N/A | Chinese | c.227_306del | p.Ala76Glyfs*58 | NR | N/A | N/A | N/A | N/A | N/A | N/A | N/A | N/A | N/A | N/A | N/A | N/A | N/A | N/A | 17 |
| 39 | N/A | | | | | | N/A | N/A | c.230_276del | p.Ala77Valfs*68 | NR | N/A | N/A | N/A | N/A | N/A | N/A | N/A | N/A | N/A | N/A | N/A | N/A | N/A | N/A | 14 |
| 40 | Female | | | | | | 1M | Chinese | c.231_232delTG | p.Ala78Glyfs*82 | NR | *De novo* | Y | Y | Y | Y | N/A | Y | Y | N/A | Y | Y | Y | Y | Y | 18 |
| 41 | Female | | | | | | 29 | Japanese | c.231_239dup | p.Ala87_Ala89dup | Uncertain significance | *De novo* | Y | N/A | N/A | Y | N/A | N/A | N/A | N/A | Y | Y | Y | Y | N/A | 19 |
| 42 | Male | | | | | | 8 | Turkish | c.240_260del21ins3 | p.Ala82_87del | NR | *De novo* | N/A | Y | Y | Y | N/A | Y | N/A | N | Y | Y | Y | N | N/A | 2 |
| 43 | N/A | | | | | | 24 | Chinese | c.243_260del | p.Ala84_Ala89del | NR | *De novo* | N/A | Y | N/A | N/A | N/A | N/A | N/A | N/A | Y | Y | N/A | N/A | N/A | 20 |
| 44 | Female | | | | | | 6M | Saudi Arabia | c.243_260del | p.Ala84_Ala89del |  | Maternal | Y | N/A | Y | Y | N/A | N/A | N/A | N/A | N/A | Y | Y | N/A | N/A | 21 |
| 45 | N/A | | | | | | N/A | Italian | c.243_260del | p.Ala84_Ala89del |  | N/A | N/A | N/A | N/A | N/A | N/A | N/A | N/A | N/A | N/A | N/A | N/A | N/A | N/A | 22 |
| 46 | Male | | | | | | 32 | Turkish | c.243_260del | p.Ala84_Ala89del |  | *De novo* | N/A | N/A | Y | Y | N/A | Y | N/A | N | Y | Y | N | N | N/A | 2 |
| 47 | Male | | | | | | 11Y 10M | Korean | c.278T>A | p.Leu93* | Pathogenic | Paternal | N | N/A | N/A | Y | N/A | N/A | N/A | N/A | Y | Y | N/A | N/A | N/A | 23 |
| 48 | Male | | | | | | N/A | Korean | c.278T>A | p.Leu93* |  | N/A | N/A | N/A | N/A | N/A | N/A | N/A | N/A | N/A | N/A | N/A | N/A | N/A | N/A | 23 |
| 49 | N/A | | | | | | N/A | N/A | c.274delC | p.Arg92Glyfs*52 | Pathogenic | N/A | N/A | N/A | N/A | N/A | N/A | N/A | N/A | N/A | N/A | N/A | N/A | N/A | N/A | 14 |
| 50 | N/A | | | | | | N/A | N/A | c.276_277ins17 | p.Leu93* | NR | N/A | N/A | N/A | N/A | N/A | N/A | N/A | N/A | N/A | N/A | N/A | N/A | N/A | N/A | 14 |
| 51 | N/A | | | | | | N/A | N/A | c.278T>A | p.Leu93* | Pathogenic | N/A | N/A | N/A | N/A | N/A | N/A | N/A | N/A | N/A | N/A | N/A | N/A | N/A | N/A | 14 |
| 52 | N/A | | | | | | N/A | Caucasian | c.278insT | p.Leu93Phefs*68 | NR | N/A | N/A | Y | N/A | N/A | N/A | N/A | N/A | N/A | Y | Y | N/A | N/A | N/A | 24 |
| 53 | N/A | | | | | | N/A | N/A | c.282delG | p.Pro95Argfs*49 | NR | N/A | N/A | N/A | N/A | N/A | N/A | N/A | N/A | N/A | N/A | N/A | N/A | N/A | N/A | 14 |
| 54 | N/A | | | | | | N/A | Danish | c.296insA | p.Asn99Lysfs*62 | NR | Familial | Y | N/A | Y | N/A | N/A | N/A | N/A | N/A | Y | N/A | N/A | N/A | N/A | 25 |
| 55 | N/A | | | | | | N/A | Danish | c.296insA | p.Asn99Lysfs*62 |  | Familial | Y | N/A | Y | N/A | N/A | N/A | N/A | N/A | Y | N/A | N/A | N/A | N/A | 25 |
| 56 | N/A | | | | | | N/A | Danish | c.296insA | p.Asn99Lysfs*62 |  | Familial | Y | N/A | Y | N/A | N/A | N/A | N/A | N/A | Y | N/A | N/A | N/A | N/A | 25 |
| 57 | N/A | | | | | | N/A | Danish | c.296insA | p.Asn99Lysfs*62 |  | Familial | N/A | N/A | N/A | N/A | N/A | N/A | N/A | N/A | N/A | N/A | N/A | N/A | N/A | 25 |
| 58 | N/A | | | | | | N/A | N/A | c.329delC | p.Pro110Argfs*34 | Pathogenic | N/A | N/A | N/A | N/A | N/A | N/A | N/A | N/A | N/A | N/A | N/A | N/A | N/A | N/A | 14 |
| 59 | Male | | | | | | 21 | Korean | c.334G>T | p.Glu112* | NR | Familial | N/A | N/A | N/A | N/A | N/A | N/A | N/A | N/A | Y | N/A | N/A | N/A | N/A | 3 |
| 60 | Male | | | | | | 16 | Chinese | c.338T>G | p.Leu113Arg | NR | Familial | Y | Y | N/A | N/A | N/A | N/A | N/A | N/A | Y | Y | N/A | N/A | N/A | 15 |
| 61 | N/A | | | | | | N/A | N/A | c.338T>G/366_367GT>TC | p.Leu113Arg and p.Cys123Arg | NR | N/A | N/A | N/A | N/A | N/A | N/A | N/A | N/A | N/A | N/A | N/A | N/A | N/A | N/A | 13 |
| 62 | N/A | | | | | | N/A | Chinese | c.346T>A | p.Trp116Arg | NR | N/A | N/A | N/A | N/A | N/A | N/A | N/A | N/A | N/A | N/A | N/A | N/A | N/A | N/A | 26 |
| 63 | N/A | | | | | | N/A | N/A | c.353G>A | p.Ser118Asn | NR | N/A | N/A | N/A | N/A | N/A | N/A | N/A | N/A | N/A | N/A | N/A | N/A | N/A | N/A | 14 |
| 64 | Male | | | | | | 16 | Turkish | c.354C>A | p.Ser118Arg | Likely pathogenic | Familial | N/A | Y | Y | Y | N/A | Y | N/A | N | Y | Y | N | N | N/A | 2 |
| 65 | Male | | | | | | 41 | Turkish | c.354C>A | p.Ser118Arg |  | Familial | N/A | Y | Y | Y | N/A | Y | N/A | N | Y | Y | Y | N | N/A | 2 |
| 66 | N/A | | | | | | N/A | Danish | c.354C>A | p.Ser118Arg |  | Familial | N/A | N/A | N | N/A | N/A | N/A | N/A | N/A | Y | N/A | N/A | N/A | N/A | 25 |
| 67 | N/A | | | | | | N/A | Danish | c.354C>A | p.Ser118Arg |  | Familial | N | N/A | Y | N/A | N/A | N/A | N/A | N/A | Y | N/A | N/A | N/A | N/A | 25 |
| 68 | N/A | | | | | | N/A | Danish | c.354C>A | p.Ser118Arg |  | Familial | Y | N/A | N | N/A | N/A | N/A | N/A | N/A | Y | N/A | N/A | N/A | N/A | 25 |
| 69 | N/A | | | | | | N/A | N/A | c.354C>G | p.Ser118Arg |  | N/A | N/A | N/A | N/A | N/A | N/A | N/A | N/A | N/A | N/A | N/A | N/A | N/A | N/A | 13 |
| 70 | Female | | | | | | 8 | Japanese | c.371C>G | p.Ser124Trp | Uncertain significance | Maternal | N/A | Y | N/A | N/A | N/A | N/A | N/A | Y | Y | Y | N/A | N/A | N/A | 27 |
| 71 | Male | | | | | | 10 | Japanese | c.371C>G | p.Ser124Trp |  | Maternal | N/A | N | N/A | N/A | N/A | N/A | N/A | Y | Y | Y | N/A | N/A | N/A | 27 |
| 72 | Female | | | | | | N/A | Japanese | c.371C>G | p.Ser124Trp |  | *De novo* | N/A | N | N/A | N/A | N/A | N/A | N/A | N/A | N/A | Y | N/A | N/A | N/A | 27 |
| 73 | Female | | | | | | 8Y9M | Hispanic | c.379C>T | p.Pro127Ser | Likely pathogenic | Maternal | Y | N/A | Y | Y | N/A | Y | N/A | N/A | Y | N/A | N/A | Y | N/A | 28 |
| 74 | Female | | | | | | N/A | Hispanic | c.379C>T | p.Pro127Ser |  | *De novo* | N/A | N/A | N/A | N/A | N/A | N/A | N/A | N/A | N/A | N/A | N/A | N/A | N/A | 28 |
| 75 | Male | | | | | | 16 | Japanese | c.382insT | p.Ser128Phefs*33 | NR | *De novo* | Y | Y | N/A | N/A | N/A | N/A | N/A | N/A | Y | Y | N/A | N/A | N/A | 29 |
| 76 | N/A | | | | | | N/A | N/A | c.383C>A | p.Ser128* | NR | N/A | N/A | N/A | N/A | N/A | N/A | N/A | N/A | N/A | N/A | N/A | N/A | N/A | N/A | 14 |
| 77 | N/A | | | | | | N/A | Caucasian | c.386A>G | p.His129Arg | Uncertain significance | N/A | N/A | Y | N/A | N/A | N/A | N/A | N/A | N/A | Y | Y | N/A | N/A | N/A | 24 |
| 78 | Female | | | | | | N/A | Italian | c.389G>A | p.Trp130* | Pathogenic | Paternal | Y | Y | N/A | N/A | N/A | N/A | N/A | N/A | N/A | Y | N/A | N/A | N/A | 4 |
| 79 | Male | | | | | | N/A | Italian | c.389G>A | p.Trp130* |  | N/A | N/A | N/A | N/A | N/A | N/A | N/A | N/A | N/A | N/A | N/A | N/A | N/A | N/A | 4 |
| 80 | N/A | | | | | | N/A | N/A | c.391C>A | p.Arg131Ser | Uncertain significance | N/A | N/A | N/A | N/A | N/A | N/A | N/A | N/A | N/A | N/A | N/A | N/A | N/A | N/A | 14 |
| 81 | N/A | | | | | | N/A | N/A | c.391C>T | p.Arg131Cys | NR | N/A | N/A | N/A | N/A | N/A | N/A | N/A | N/A | N/A | N/A | N/A | N/A | N/A | N/A | 14 |
| 82 | Female | | | | | | 7M | Polish | c.391C>T | p.Arg131Cys |  | *De novo* | Y | Y | Y | N/A | N | N/A | N/A | N/A | Y | Y | N/A | Y | N/A | 30 |
| 83 | Female | | | | | | 11 | Italian | c.391C>T | p.Arg131Cys |  | *De novo* | N/A | Y | Y | N/A | Y | N/A | N/A | N/A | Y | Y | N/A | N/A | N/A | 31 |
| 84 | Female | | | | | | 13 | Korean | c.391C>G | p.Arg131Gly | Likely pathogenic | *De novo* | N/A | N/A | N/A | N/A | N/A | N/A | N/A | N/A | Y | N/A | N/A | N/A | N/A | 3 |
| 85 | N/A | | | | | | N/A | N/A | c.396C>A | p.Cys132* | NR | N/A | N/A | N/A | N/A | N/A | N/A | N/A | N/A | N/A | N/A | N/A | N/A | N/A | N/A | 14 |
| 86 | N/A | | | | | | N/A | Italian | c.397_399delAAC | p.Asn133del | NR | Familial | Y | Y | N/A | N/A | N/A | N/A | N/A | N/A | N/A | Y | N/A | N/A | N/A | 4 |
| 87 | Female | | | | | | 17 | Chinese | c.398–399insACAGCAGCAGCAGCA and c.411–412insG | p.Asn133delinsLysGlnGlnGlnGlnHis/p.Val138Glyfs*23 | NR | *De novo* | Y | Y | N/A | N/A | N/A | N/A | N/A | Y | Y | Y | N/A | N/A | N/A | 32 |
| 88 | N/A | | | | | | N/A | N/A | c.399_401delCAA | p.Asn134del | NR | *De novo* | N/A | Y | N/A | N/A | N/A | N/A | N/A | N/A | N/A | Y | N/A | N/A | N/A | 7 |
| 89 | Male | | | | | | 3Y 6M | Polish | c.407T>A | p.Leu136Gln | NR | *De novo* | Y | Y | Y | N/A | N | N/A | N/A | N/A | Y | Y | N/A | Y | N/A | 30 |
| 90 | N/A | | | | | | N/A | N/A | c.407T>C | p.Leu136Pro | Pathogenic | N/A | N/A | N/A | N/A | N/A | N/A | N/A | N/A | N/A | N/A | N/A | N/A | N/A | N/A | 14 |
| 91 | Female | | | | | 76 | | Chinese | c.407T>C | p.Leu136Pro |  | N/A | Y | N/A | Y | N/A | N/A | N/A | N/A | N/A | N/A | N/A | N/A | Y | N/A | 33 |
| 92 | Female | | | | | 52 | | Chinese | c.407T>C | p.Leu136Pro |  | Maternal | Y | Y | N | N/A | N/A | N/A | N/A | N/A | N/A | N/A | N/A | Y | N/A | 33 |
| 93 | Male | | | | | 49 | | Chinese | c.407T>C | p.Leu136Pro |  | Maternal | Y | N/A | N | N/A | N/A | N/A | N/A | N/A | N/A | Y | N/A | Y | N/A | 33 |
| 94 | Male | | | | | 40 | | Chinese | c.407T>C | p.Leu136Pro |  | Maternal | Y | N/A | Y | N/A | N/A | N/A | N/A | N/A | Y | Y | Y | Y | N/A | 33 |
| 95 | Female | | | | | 34 | | Chinese | c.407T>C | p.Leu136Pro |  | Maternal | Y | N/A | Y | N/A | N/A | Y | Y | Y | N/A | Y | N/A | Y | N/A | 33 |
| 96 | Male | | | | | 33 | | Chinese | c.407T>C | p.Leu136Pro |  | Maternal | Y | Y | N | N/A | N/A | N/A | N/A | N/A | N/A | Y | N/A | Y | N/A | 33 |
| 97 | Female | | | | | 26 | | Chinese | c.407T>C | p.Leu136Pro |  | Paternal | Y | N/A | N | N/A | N/A | N/A | N/A | N/A | N/A | N/A | N/A | Y | N/A | 33 |
| 98 | Female | | | | | 12 | | Chinese | c.407T>C | p.Leu136Pro |  | Maternal | Y | N/A | Y | N/A | N/A | N/A | N/A | N/A | N/A | N | N/A | Y | N/A | 33 |
| 99 | Male | | | | | 3 | | Chinese | c.407T>C | p.Leu136Pro |  | Maternal | Y | N/A | Y | N/A | N/A | N/A | N/A | N/A | N/A | Y | N/A | Y | N/A | 33 |
| 100 | Male | | | | | 4 | | Chinese | c.407T>C | p.Leu136Pro |  | Paternal | Y | N/A | Y | N/A | N/A | N/A | N/A | N/A | N/A | Y | N/A | Y | N/A | 33 |
| 101 | Male | | | | | 2 | | Chinese | c.407T>C | p.Leu136Pro |  | Maternal | Y | N/A | Y | N/A | N/A | N/A | N/A | N/A | N/A | Y | N/A | Y | N/A | 33 |
| 102 | Male | | | | | 7.5 | | Turkish | c.423+2delT | - | Likely pathogenic | Familial | N/A | Y | N | Y | N/A | N | N/A | Y | Y | Y | Y | Y | N/A | 2 |
| 103 | Male | | | | | 1M | | Turkish | c.423+2delT | - |  | Familial | N/A | N/A | N | Y | N/A | N | N/A | N | Y | Y | N | N | N/A | 2 |
| 104 | Male | | | | | 39 | | Turkish | c.423+2delT | - |  | Familial | N/A | Y | N | Y | N/A | N | N/A | N/A | Y | N | N | N | N/A | 2 |
| 105 | Female | | | | | N/A | | Japanese | c.424-1G>T | - | NR | N/A | N/A | N/A | N/A | N/A | N/A | N/A | N/A | N/A | N/A | N/A | N/A | N/A | N/A | 5 |
| 106 | Male | | | | | 13 | | Japanese | c.425insT | p.Val143Glyfs*18 | NR | *De novo* | Y | Y | N/A | N/A | N/A | N/A | N/A | N/A | Y | Y | N/A | N/A | N/A | 34 |
| 107 | Male | | | | | 11 | | Caucasian | c.436G>A | p.Gly146Arg | Conflicting classifications of pathogenicity | Familial | N/A | N/A | Y | Y | N/A | N/A | N/A | N/A | Y | N/A | N/A | N/A | N/A | 12 |
| 108 | Male | | | | | 37 | | Caucasian | c.436G>A | p.Gly146Arg |  | Familial | N/A | N/A | Y | Y | N/A | N/A | N/A | N/A | Y | N/A | N/A | N/A | N/A | 12 |
| 109 | Male | | | | | 10 | | Turkish | c.443_454delTACCAGATGGGAinsG | p.Val148Glyfs*9 | Pathogenic | *De novo* | N/A | Y | Y | Y | N/A | Y | N/A | Y | Y | Y | Y | N | N/A | 2 |
| 110 | Female | | | | | 5Y 6M | | Turkish | c.463A>C | p.Thr155Pro | NR | *De novo* | Y | Y | Y | Y | N/A | Y | N/A | Y | Y | Y | Y | Y | N/A | 35 |
| 111 | N/A | | | | | N/A | | N/A | c.467T>A | p.Val156Asp | Likely pathogenic | N/A | N/A | N/A | N/A | N/A | N/A | N/A | N/A | N/A | N/A | N/A | N/A | N/A | N/A | 14 |
| 112 | N/A | | | | | N/A | | N/A | c.469insA | p.Met157Asnfs*4 | NR | N/A | N/A | N/A | N/A | N/A | N/A | N/A | N/A | N/A | N/A | N/A | N/A | N/A | N/A | 14 |
| 113 | Female | | | | | 27 | | Indian | c.470insT | p.Met157Ilefs*4 | NR | Maternal | Y | Y | N/A | Y | N/A | N/A | N/A | N/A | N/A | Y | N/A | Y | N/A | 36 |
| 114 | N/A | | | | | N/A | | Chinese | c.471_472dupGG | p.Ala158Glyfs*19 | NR | N/A | N/A | N/A | N/A | N/A | N/A | N/A | N/A | N/A | N/A | N/A | N/A | N/A | N/A | 17 |
| 115 | Female | | | | | 16 | | Chinese | c.473C>A | p.Ala158Glu | NR | Maternal | Y | N/A | Y | Y | N/A | Y | Y | Y | Y | N | N/A | N/A | N/A | 37 |
| 116 | Female | | | | | 48 | | Chinese | c.473C>A | p.Ala158Glu |  | *De novo* | Y | N/A | Y | Y | N/A | Y | Y | Y | Y | N | N/A | N/A | N/A | 37 |
| 117 | Male | | | | | 9 | | Japanese | c.473C>A | p.Ala158Glu |  | *De novo* | N | Y | N | Y | N | N | Y | N | Y | Y | Y | N | N | 128 |
| 118 | Female | | | | | N/A | | Chinese | c.475G>C | p.Gly159Arg | NR | *De novo* | Y | Y | N/A | Y | N/A | N/A | N/A | N/A | Y | Y | Y | N/A | N/A | 38 |
| 119 | Female | | | | | 29 | | Chinese | c.475G>C | p.Gly159Arg |  | N/A | N/A | Y | N/A | Y | N/A | N/A | N/A | Y | Y | Y | N/A | N/A | N/A | 38 |
| 120 | Male | | | | | 22 | | N/A | c.476G>A | p.Gly159Asp | Pathogenic/Likely pathogenic | *De novo* | N | Y | Y | Y | Y | Y | N/A | N/A | Y | Y | Y | N/A | N/A | 39 |
| 121 | Female | | | | | 52 | | Han chinese | c.476delG | p.Gly159fs*175 | NR | *De novo* | Y | N/A | N/A | Y | N/A | Y | N/A | Y | Y | Y | N/A | N/A | N/A | 40 |
| 122 | Male | | | | | 2 | | Polish | c.480C>G | p.Asn160Lys | Likely pathogenic | Familial | Y | Y | Y | N/A | N | N/A | N/A | N/A | Y | Y | N/A | Y | N/A | 30 |
| 123 | N/A | | | | | N/A | | N/A | c.481_482delGA | p.Asp161* | NR | N/A | N/A | N/A | N/A | N/A | N/A | N/A | N/A | N/A | N/A | N/A | N/A | N/A | N/A | 13 |
| 124 | N/A | | | | | N/A | | N/A | c.484G>T | p.Glu162* | NR | N/A | N/A | N/A | N/A | N/A | N/A | N/A | N/A | N/A | N/A | N/A | N/A | N/A | N/A | 14 |
| 125 | N/A | | | | | N/A | | Japanese | c.495delT | A166Lfs*10 | Likely pathogenic | N/A | N/A | Y | N/A | N/A | N/A | N/A | N/A | N/A | Y | Y | N/A | N/A | N/A | 9 |
| 126 | N/A | | | | | N/A | | Japanese | c.495delT | A166Lfs*10 |  | N/A | N/A | Y | N/A | N/A | N/A | N/A | N/A | N/A | N/A | Y | N/A | N/A | N/A | 9 |
| 127 | N/A | | | | | N/A | | N/A | c.497_506del | p.Ala166Glyfs*7 | NR | N/A | N/A | N/A | N/A | N/A | N/A | N/A | N/A | N/A | N/A | N/A | N/A | N/A | N/A | 41 |
| 128 | Male | | | | | 4M | | Turkish | c.505C>T | p.Arg169Trp | Pathogenic/Likely pathogenic | Familial | Y | Y | Y | Y | N/A | Y | N/A | N | N | Y | Y | N | N/A | 2 |
| 129 | Male | | | | | 32 | | Turkish | c.505C>T | p.Arg169Trp |  | Familial | N/A | Y | Y | Y | N/A | Y | N/A | N/A | Y | Y | N | N | N/A | 2 |
| 130 | Female | | | | | 26 | | Italian | c.505C>T | p.Arg169Trp |  | N/A | Y | N/A | N/A | N/A | N/A | N/A | N/A | N/A | N/A | N/A | Y | N/A | N/A | 42 |
| 131 | N/A | | | | | N/A | | N/A | c.506G>A | p.Arg169Gln | Conflicting classifications of pathogenicity | Familial | N/A | Y | N/A | N/A | N/A | N/A | N/A | N/A | N/A | Y | N/A | N/A | N/A | 7 |
| 132 | Female | | | | | 33 | | N/A | c.506G>C | p.Arg169Pro | Pathogenic | *De novo* | N/A | Y | Y | N/A | N/A | N/A | N/A | N/A | Y | Y | Y | N/A | N/A | 43 |
| 133 | Female | | | | | 8 | | N/A | c.506G>C | p.Arg169Pro |  | Maternal | N/A | Y | N/A | N/A | N/A | N/A | N/A | N/A | Y | Y | Y | Y | N/A | 43 |
| 134 | Male | | | | | 10 | | Chinese | c.514delT | p.Ser172Leufs*4 | NR | Paternal | Y | Y | Y | N | N/A | N/A | N/A | N/A | Y | Y | N/A | N/A | N/A | 44 |
| 135 | Male | | | | | 20 | | Chinese | c.514delT | p.Ser172Leufs*4 |  | *De novo* | Y | Y | Y | Y | N/A | N/A | N/A | N/A | Y | Y | N/A | N/A | N/A | 44 |
| 136 | N/A | | | | | N/A | | Italian | c.522insA | p.Met175fs*218 | NR | Familial | Y | Y | N/A | N/A | N/A | N/A | N/A | N/A | N/A | Y | N/A | N/A | N/A | 4 |
| 137 | N/A | | | | | N/A | | N/A | c.523A>G | p.Met175Val | Conflicting classifications of pathogenicity | N/A | N/A | N/A | N/A | N/A | N/A | N/A | N/A | N/A | N/A | N/A | N/A | N/A | N/A | 14 |
| 138 | N/A | | | | | N/A | | N/A | c.524T>A | p.Met175Lys | NR | N/A | N/A | N/A | N/A | N/A | N/A | N/A | N/A | N/A | N/A | N/A | N/A | N/A | N/A | 14 |
| 139 | Male | | | | | 3Y 6M | | N/A | c.524T>G | p.Met175Arg | Likely pathogenic | *De novo* | Y | N/A | Y | Y | N/A | N/A | N/A | N/A | N/A | Y | N/A | N/A | N/A | 45 |
| 140 | N/A | | | | | N/A | | N/A | c.524T>G | p.Met175Arg |  | *De novo* | N/A | Y | N/A | N/A | N/A | N/A | N/A | N/A | N/A | Y | N/A | N/A | N/A | 7 |
| 141 | N/A | | | | | N/A | | N/A | c.524T>G | p.Met175Arg |  | N/A | N/A | N/A | N/A | N/A | N/A | N/A | N/A | N/A | N/A | N/A | N/A | N/A | N/A | 13 |
| 142 | Female | | | | | N/A | | Korean | c.524T>G | p.Met175Arg |  | N/A | N/A | N/A | N/A | N/A | N/A | N/A | N/A | N/A | N/A | N/A | N/A | N/A | N/A | 46 |
| 143 | Male | | | | | N/A | | Korean | c.524T>G | p.Met175Arg |  | N/A | N/A | N/A | N/A | N/A | N/A | N/A | N/A | N/A | N/A | N/A | N/A | N/A | N/A | 46 |
| 144 | Female | | | | | N/A | | Polish | c.532C>T | p.Gln178* | NR | N/A | N/A | N/A | N/A | N/A | N/A | N/A | N/A | N/A | N/A | Y | N/A | N/A | N/A | 47 |
| 145 | Female | | | | | N/A | | Polish | c.532C>T | p.Gln178* |  | Maternal | Y | Y | N/A | N/A | N/A | N/A | N/A | N/A | Y | Y | N/A | N/A | N/A | 47 |
| 146 | Female | | | | | N/A | | Polish | c.532C>T | p.Gln178* |  | Maternal | Y | Y | N/A | N/A | N/A | N/A | N/A | N/A | N/A | Y | N/A | N/A | N/A | 47 |
| 147 | N/A | | | | | N/A | | Japanese | c.532delC | p.Gln178Lysfs*2 | NR | N/A | N/A | N/A | N/A | N/A | N/A | N/A | N/A | N/A | N/A | N/A | N/A | N/A | N/A | 5 |
| 148 | Male | | | | | 12 | | Chinese | c.534dupA | p.Val179Serfs*40 | NR | Familial | Y | N/A | Y | Y | N/A | Y | Y | N/A | Y | Y | N/A | Y | N/A | 129 |
| 149 | Male | | | | | 1 | | Mexican | c.538_539delincCA | p.Ala180Gln | NR | *De novo* | N/A | Y | Y | N/A | N/A | Y | N/A | N/A | Y | Y | N/A | N | N | 77 |
| 150 | N/A | | | | | N/A | | N/A | c.540_549del10 | p.Arg181Ilefs*2 | NR | N/A | N/A | N/A | N/A | N/A | N/A | N/A | N/A | N/A | N/A | N/A | N/A | N/A | N/A | 13 |
| 151 | Female | | | | | 18 | | Chinese | c.549delC | p.Asn183fs*185 | NR | Paternal | N/A | Y | Y | Y | N/A | Y | Y | Y | Y | Y | N/A | N/A | N/A | 48 |
| 152 | Male | | | | | N/A | | Chinese | c.549delC | p.Asn183fs*185 |  | Paternal | N/A | Y | Y | Y | N/A | N/A | N/A | Y | N/A | Y | N/A | N/A | N/A | 48 |
| 153 | N/A | | | | | N/A | | N/A | c.553_554delCT | p.Leu185Glufs*33 | NR | Familial | N/A | Y | N/A | N/A | N/A | N/A | N/A | N/A | N/A | Y | N/A | N/A | N/A | 7 |
| 154 | Female | | | | | 42 | | Chinese | c.557G>C | p.Arg186Thr | NR | *De novo* | N | Y | N/A | N/A | N/A | N/A | N/A | N/A | Y | Y | N/A | N/A | N/A | 15 |
| 155 | N/A | | | | | N/A | | N/A | c.560T>C | p.Phe187Ser | NR | N/A | N/A | N/A | N/A | N/A | N/A | N/A | N/A | N/A | N/A | N/A | N/A | N/A | N/A | 14 |
| 156 | N/A | | | | | N/A | | Italian | c.568C>T | p.Arg190Trp | Pathogenic | *De novo* | N/A | N/A | N/A | N/A | N/A | N/A | N/A | N/A | N/A | N/A | N/A | N/A | N/A | 49 |
| 157 | N/A | | | | | N/A | | Italian | c.568C>T | p.Arg190Trp |  | *De novo* | N/A | N/A | N/A | N/A | N/A | N/A | N/A | N/A | N/A | N/A | N/A | N/A | N/A | 49 |
| 158 | N/A | | | | | N/A | | Italian | c.568C>T | p.Arg190Trp |  | *De novo* | N/A | N/A | N/A | N/A | N/A | N/A | N/A | N/A | N/A | N/A | N/A | N/A | N/A | 49 |
| 159 | N/A | | | | | N/A | | Italian | c.568C>T | p.Arg190Trp |  | *De novo* | N/A | N/A | N/A | N/A | N/A | N/A | N/A | N/A | N/A | N/A | N/A | N/A | N/A | 49 |
| 160 | Female | | | | | 26 | | N/A | c.568C>T | p.Arg190Trp |  | *De novo* | N/A | Y | Y | N/A | N/A | Y | N/A | N/A | Y | Y | N/A | Y | N/A | 50 |
| 161 | Male | | | | | 4Y 6M | | N/A | c.568C>T | p.Arg190Trp |  | Maternal | N/A | Y | Y | N/A | N/A | Y | N/A | N/A | Y | Y | N/A | N/A | N/A | 50 |
| 162 | N/A | | | | | N/A | | Japanese | c.568C>T | p.Arg190Trp |  | N/A | N/A | Y | N/A | N/A | N/A | N/A | N/A | N/A | N/A | Y | N/A | N/A | N/A | 9 |
| 163 | Female | | | | | N/A | | Italian | c.568C>T | p.Arg190Trp |  | N/A | N/A | N/A | N/A | N/A | N/A | N/A | N/A | N/A | N/A | N/A | N/A | N/A | N/A | 4 |
| 164 | Male | | | | | N/A | | Italian | c.568C>T | p.Arg190Trp |  | Maternal | N/A | N/A | N/A | N/A | N/A | N/A | N/A | N/A | N/A | N/A | N/A | N/A | N/A | 4 |
| 165 | Male | | | | | N/A | | Italian | c.568C>T | p.Arg190Trp |  | Paternal | Y | Y | N/A | N/A | N/A | N/A | N/A | N/A | N/A | Y | N/A | N/A | N/A | 4 |
| 166 | N/A | | | | | N/A | | Italian | c.568C>T | p.Arg190Trp |  | *De novo* | N/A | Y | N/A | N/A | N/A | N/A | N/A | N/A | N/A | Y | N/A | N/A | N/A | 4 |
| 167 | Female | | | | | 5 | | Chinese | c.568C>T | p.Arg190Trp |  | N/A | Y | Y | N/A | Y | N/A | N/A | Y | N/A | Y | Y | N/A | N/A | N/A | 51 |
| 168 | Male | | | | | 7 | | Caucasian | c.568C>T | p.Arg190Trp |  | Familial | N/A | N/A | Y | Y | N/A | N/A | N/A | N/A | Y | N/A | N/A | N/A | N/A | 12 |
| 169 | Female | | | | | 25 | | Caucasian | c.568C>T | p.Arg190Trp |  | Familial | N/A | N/A | Y | Y | N/A | N/A | N/A | N/A | Y | N/A | N/A | N/A | N/A | 12 |
| 170 | N/A | | | | | N/A | | Polish | c.568C>T | p.Arg190Trp |  | *De novo* | Y | Y | Y | N/A | N/A | N/A | N/A | N/A | Y | Y | Y | N/A | N/A | 52 |
| 171 | N/A | | | | | N/A | | Danish | c.568C>T | p.Arg190Trp |  | *De novo* | N | N/A | Y | N/A | N/A | N/A | N/A | N/A | Y | N/A | N/A | N/A | N/A | 25 |
| 172 | N/A | | | | | N/A | | Danish | c.568C>T | p.Arg190Trp |  | Familial | Y | N/A | Y | N/A | N/A | N/A | N/A | N/A | Y | N/A | N/A | N/A | N/A | 25 |
| 173 | N/A | | | | | N/A | | Danish | c.568C>T | p.Arg190Trp |  | Familial | Y | N/A | Y | N/A | N/A | N/A | N/A | N/A | Y | N/A | N/A | N/A | N/A | 25 |
| 174 | Male | | | | | 10 | | Taiwanese | c.568C>T | p.Arg190Trp |  | Maternal | Y | N/A | Y | N/A | N/A | N/A | N/A | N/A | Y | Y | Y | N/A | N/A | 53 |
| 175 | Female | | | | | 40 | | Taiwanese | c.568C>T | p.Arg190Trp |  | N/A | Y | N/A | Y | N/A | N/A | N/A | N/A | N/A | Y | Y | Y | N/A | N/A | 53 |
| 176 | N/A | | | | | N/A | | Polish | c.568C>T | p.Arg190Trp |  | Familial | Y | Y | Y | N/A | N | N/A | N/A | N/A | Y | Y | N/A | Y | N/A | 30 |
| 177 | Male | | | | | 8M | | Polish | c.568C>T | p.Arg190Trp |  | *De novo* | Y | Y | Y | N/A | N | N/A | N/A | N/A | Y | Y | N/A | Y | N/A | 30 |
| 178 | Male | | | | | 13 | | Chinese | c.568C>T | p.Arg190Trp |  | *De novo* | N | Y | Y | Y | N | Y | Y | N/A | Y | Y | Y | Y | N/A | 54 |
| 179 | Male | | | | | 13 | | Chinese | c.568C>T | p.Arg190Trp |  | *De novo* | N | Y | Y | Y | N | Y | Y | N/A | Y | Y | Y | Y | N/A | 54 |
| 180 | 29 | | | | |  | | Chinese | c.568C>T | p.Arg190Trp |  | Maternal | N/A | Y | N/A | N/A | N/A | N/A | N/A | N/A | Y | N/A | N/A | N/A | N/A | 55 |
| 181 | Female | | | | | 49 | | N/A | c.568C>T | p.Arg190Trp |  | N/A | N | Y | Y | Y | N | Y | N/A | N/A | N/A | Y | N | N/A | N/A | 39 |
| 182 | Male | | | | | 13 | | N/A | c.568C>T | p.Arg190Trp |  | Maternal | N | Y | Y | Y | N | Y | N/A | N/A | Y | Y | Y | N/A | N/A | 39 |
| 183 | Male | | | | | 12 | | N/A | c.568C>T | p.Arg190Trp |  | Maternal | Y | Y | Y | Y | N | Y | N/A | N/A | Y | Y | Y | N/A | N/A | 39 |
| 184 | Male | | | | | N/A | | N/A | c.568C>T | p. Arg190Trp |  | Paternal | N/A | Y | Y | N/A | N/A | N/A | N/A | N/A | N/A | Y | Y | N/A | N/A | 56 |
| 185 | Male | | | | | N/A | | N/A | c.568C>T | p. Arg190Trp |  | Paternal | Y | N/A | Y | N/A | Y | Y | N/A | N/A | Y | Y | Y | Y | N/A | 56 |
| 186 | Male | | | | | 2D | | Chinese | c.569G>A | p.Arg190Gln | Pathogenic | Maternal | N/A | Y | N/A | N/A | N/A | N/A | N/A | N/A | N/A | Y | N/A | N/A | N/A | 57 |
| 187 | N/A | | | | | N/A | | N/A | c.569G>A | p.Arg190Gln |  | *De novo* | N/A | Y | N/A | N/A | N/A | N/A | N/A | N/A | N/A | Y | N/A | N/A | N/A | 7 |
| 188 | Male | | | | | 18 | | N/A | c.569G>A | p.Arg190Gln |  | *De novo* | N/A | Y | Y | Y | N | N/A | N/A | Y | Y | Y | N/A | N/A | N/A | 58 |
| 189 | Male | | | | | 14 | | Caucasian | c.569G>A | p.Arg190Gln |  | *De novo* | N/A | N/A | Y | Y | N/A | N/A | N/A | N/A | Y | N/A | N/A | N/A | N/A | 12 |
| 190 | Male | | | | | 16 | | Caucasian | c.569G>A | p.Arg190Gln |  | *De novo* | N/A | N/A | Y | Y | N/A | N/A | N/A | N/A | Y | N/A | N/A | N/A | N/A | 12 |
| 191 | N/A | | | | | N/A | | Caucasian | c.569G>A | p.Arg190Gln |  | N/A | N/A | Y | N/A | N/A | N/A | N/A | N/A | N/A | Y | Y | N/A | N/A | N/A | 24 |
| 192 | Male | | | | | 19 | | Chinese | c.569G>A | p.Arg190Gln |  | *De novo* | Y | Y | N/A | N/A | N/A | N/A | N/A | N/A | Y | Y | N/A | N/A | N/A | 44 |
| 193 | N/A | | | | | N/A | | Danish | c.569G>A | p.Arg190Gln |  | *De novo* | Y | N/A | Y | N/A | N/A | N/A | N/A | N/A | Y | N/A | N/A | N/A | N/A | 25 |
| 194 | Female | | | | | 82 | | N/A | c.569G>A | p.Arg190Gln |  | N/A | N/A | Y | N/A | N/A | N/A | N/A | N/A | N/A | N/A | Y | N/A | N/A | N/A | 59 |
| 195 | Male | | | | | N/A | | Korean | c.569G>A | p.Arg190Gln |  | N/A | N/A | N/A | N/A | N/A | N/A | N/A | N/A | N/A | N/A | N/A | N/A | N/A | N/A | 46 |
| 196 | Male | | | | | N/A | | Korean | c.569G>A | p.Arg190Gln |  | N/A | N/A | N/A | N/A | N/A | N/A | N/A | N/A | N/A | N/A | N/A | N/A | N/A | N/A | 46 |
| 197 | Male | | | | | 10 | | Chinese | c.569G>A | p.Arg190Gln |  | Maternal | N | Y | Y | Y | N/A | Y | N/A | Y | Y | Y | Y | Y | N/A | 1 |
| 198 | Female | | | | | 20 | | Chinese | c.569G>A | p.Arg190Gln |  | Maternal | Y | Y | Y | Y | N/A | Y | N/A | Y | Y | Y | N | N/A | N/A | 1 |
| 199 | Female | | | | | N/A | | Chinese | c.569G>A | p.Arg190Gln |  | Familial | N/A | N/A | N/A | N/A | N/A | N/A | N/A | N/A | N/A | N/A | N/A | N/A | N/A | 1 |
| 200 | Male | | | | | 6 | | Turkish | c.569G>A | p.Arg190Gln |  | *De novo* | N/A | Y | Y | Y | N/A | Y | N/A | Y | Y | Y | Y | N | N/A | 2 |
| 201 | N/A | | | | | N/A | | Danish | c.569G>C | p.Arg190Pro | NR | *De novo* | Y | N/A | N/A | N/A | N/A | N/A | N/A | N/A | Y | N/A | N/A | N/A | N/A | 25 |
| 202 | Female | | | | | 5M | | N/A | c.572G>A | p.Ser191Asn | Likely pathogenic | *De novo* | Y | Y | Y | N/A | N/A | N/A | N/A | N/A | Y | Y | Y | N/A | N/A | 45 |
| 203 | N/A | | | | | N/A | | N/A | c.572G>A | p.Ser191Asn |  | N/A | N/A | N/A | N/A | N/A | N/A | N/A | N/A | N/A | N/A | N/A | N/A | N/A | N/A | 13 |
| 204 | N/A | | | | | N/A | | N/A | c.572G>A | p.Ser191Asn |  | *De novo* | N/A | Y | N/A | N/A | N/A | N/A | N/A | N/A | N/A | Y | N/A | N/A | N/A | 7 |
| 205 | Female | | | | | 1Y9M | | Chinese | c.574G>A | p.Gly192Arg | NR | *De novo* | Y | Y | Y | Y | N/A | Y | Y | N/A | Y | Y | Y | N/A | Y | 18 |
| 206 | Female | | | | | 55 | | N/A | c.574G>A | p.Gly192Arg |  | *De novo* | Y | Y | N/A | N/A | N/A | N/A | N/A | N/A | Y | Y | Y | N/A | N/A | 60 |
| 207 | Male | | | | | 27 | | N/A | c.574G>A | p.Gly192Arg |  | Maternal | Y | Y | N/A | N/A | N/A | N/A | N/A | N/A | Y | Y | N/A | N/A | N/A | 60 |
| 208 | Female | | | | | 33 | | N/A | c.574G>A | p.Gly192Arg |  | Maternal | Y | Y | N/A | N/A | N/A | N/A | N/A | N/A | Y | Y | N/A | N/A | N/A | 60 |
| 209 | N/A | | | | | N/A | | N/A | c.577C>T | p.Arg193* | Pathogenic | N/A | N/A | N/A | N/A | N/A | N/A | N/A | N/A | N/A | N/A | N/A | N/A | N/A | N/A | 13 |
| 210 | N/A | | | | | N/A | | Japanese | c.577C>T | p.Arg193* |  | N/A | N/A | N/A | N/A | N/A | N/A | N/A | N/A | N/A | N/A | N/A | N/A | N/A | N/A | 5 |
| 211 | N/A | | | | | N/A | | Japanese | c.577C>T | p.Arg193* |  | N/A | N/A | Y | N/A | N/A | N/A | N/A | N/A | N/A | N/A | Y | N/A | N/A | N/A | 9 |
| 212 | N/A | | | | | N/A | | Japanese | c.577C>T | p.Arg193* |  | N/A | N/A | Y | N/A | N/A | N/A | N/A | N/A | N/A | N/A | Y | N/A | N/A | N/A | 9 |
| 213 | N/A | | | | | N/A | | Japanese | c.577C>T | p.Arg193* |  | N/A | N/A | Y | N/A | N/A | N/A | N/A | N/A | N/A | N/A | Y | N/A | N/A | N/A | 9 |
| 214 | N/A | | | | | N/A | | Japanese | c.577C>T | p.Arg193* |  | N/A | N/A | Y | N/A | N/A | N/A | N/A | N/A | N/A | N/A | Y | N/A | N/A | N/A | 9 |
| 215 | Female | | | | | 24 | | Caucasian | c.577C>T | p.Arg193* |  | N/A | N/A | N/A | Y | Y | N/A | N/A | N/A | N/A | Y | N/A | N/A | N/A | N/A | 12 |
| 216 | Female | | | | | 16 | | Chinese | c.577C>T | p.Arg193* |  | Paternal | Y | Y | Y | Y | N/A | N/A | N/A | Y | Y | Y | N/A | N/A | N/A | 61 |
| 217 | N/A | | | | | N/A | | Danish | c.577C>T | p.Arg193* |  | *De novo* | N | N/A | Y | N/A | N/A | N/A | N/A | N/A | Y | N/A | N/A | N/A | N/A | 25 |
| 218 | Male | | | | | 3 | | Chinese | c.577C>T | p.Arg193* |  | *De novo* | Y | Y | Y | Y | N/A | N | Y | N/A | Y | Y | Y | N/A | Y | 18 |
| 219 | 32 | | | | |  | | Chinese | c.577C>T | p.Arg193* |  | N/A | N/A | Y | Y | N/A | N/A | N/A | N/A | N/A | N/A | Y | N/A | N/A | N/A | 62 |
| 220 | Female | | | | | 8 | | Chinese | c.577C>T | p.Arg193* |  | Paternal | N/A | Y | Y | N/A | N/A | N/A | N/A | N/A | Y | Y | N/A | N/A | N/A | 62 |
| 221 | Female | | | | | 1 | | Chinese | c.577C>T | p.Arg193* |  | Paternal | N/A | Y | Y | N/A | N/A | N/A | N/A | N/A | N/A | Y | N/A | N/A | N/A | 62 |
| 222 | N/A | | | | | N/A | | Polish | c.577C>T | p.Arg193* |  | *De novo* | Y | Y | Y | N/A | N/A | N/A | N/A | N/A | Y | Y | Y | N/A | N/A | 52 |
| 223 | N/A | | | | | N/A | | Polish | c.577C>T | p.Arg193* |  | *De novo* | Y | Y | Y | N/A | N/A | N/A | N/A | N/A | Y | Y | Y | N/A | N/A | 52 |
| 224 | N/A | | | | | N/A | | Polish | c.577C>T | p.Arg193* |  | *De novo* | Y | Y | Y | N/A | N/A | N/A | N/A | N/A | Y | Y | Y | N/A | N/A | 52 |
| 225 | Male | | | | | 6 | | Turkish | c.577C>T | p.Arg193* |  | *De novo* | Y | Y | N | Y | N/A | Y | N/A | Y | N | Y | N | Y | N/A | 2 |
| 226 | Male | | | | | Newborn | | Chinese | c.577C>T | p.Arg193* |  | Paternal | N/A | Y | N/A | N/A | N/A | N/A | N/A | N/A | N/A | N/A | N/A | N/A | N/A | 63 |
| 227 | Female | | | | | Newborn | | Chinese | c.577C>T | p.Arg193* |  | Paternal | N/A | Y | N/A | N/A | N/A | N/A | N/A | N/A | N/A | Y | N/A | N/A | N/A | 63 |
| 228 | Female | | | | | 9 | | N/A | c.577C>G | p.Arg193Gly | NR | *De novo* | N/A | Y | N/A | N/A | N/A | N/A | N/A | N/A | Y | Y | N/A | N/A | N/A | 64 |
| 229 | Female | | | | | 13 | | Turkish | c.577C>G | p.Arg193Gly |  | *De novo* | N/A | Y | Y | Y | N/A | N | N/A | Y | Y | Y | Y | N | N/A | 2 |
| 230 | N/A | | | | | N/A | | N/A | c.577_579delCGAinsTGT | p.Arg193Cys | NR | *De novo* | N/A | Y | N/A | N/A | N/A | N/A | N/A | N/A | N/A | Y | N/A | N/A | N/A | 7 |
| 231 | N/A | | | | | N/A | | N/A | c.578G>A | p.Arg193Gln | Pathogenic/  Likely pathogenic | N/A | N/A | N/A | N/A | N/A | N/A | N/A | N/A | N/A | N/A | N/A | N/A | N/A | N/A | 14 |
| 232 | Female | | | | | 20 | | Chinese | c.578G>A | p.Arg193Gln |  | *De novo* | Y | Y | Y | Y | N/A | Y | Y | Y | Y | Y | N/A | N/A | N/A | 65 |
| 233 | Male | | | | | 3.6 | | Korean | c.578G>A | p.Arg193Gln |  | *De novo* | N/A | Y | Y | N/A | N/A | Y | N/A | N/A | Y | Y | Y | N/A | N/A | 66 |
| 234 | N/A | | | | | N/A | | Chinese | c.578G>C | p.Arg193Pro | NR | N/A | N/A | N/A | N/A | N/A | N/A | N/A | N/A | N/A | N/A | N/A | N/A | N/A | N/A | 67 |
| 235 | Female | | | | | 27 | | Italian | c.580+1G>A | - | NR | Maternal | Y | N/A | Y | N/A | N/A | N/A | N/A | N/A | Y | Y | N/A | N/A | N/A | 68 |
| 236 | Female | | | | | 54 | | Italian | c.580+1G>A | - |  | N/A | N/A | N/A | N/A | N/A | N/A | N/A | N/A | N/A | N/A | N/A | N/A | N/A | N/A | 68 |
| 237 | Female | | | | | 24 | | Italian | c.580+1G>A | - |  | Maternal | N/A | N/A | N/A | N/A | N/A | N/A | N/A | N/A | N/A | N/A | N/A | N/A | N/A | 68 |
| 238 | N/A | | | | | N/A | | N/A | c.580+1G>T | - | NR | N/A | N/A | N/A | N/A | N/A | N/A | N/A | N/A | N/A | N/A | N/A | N/A | N/A | N/A | 14 |
| 239 | Male | | | | | 4 | | Polish | c.580+1G>T | - |  | *De novo* | Y | Y | Y | N/A | N | N/A | N/A | N/A | Y | Y | N/A | Y | N/A | 30 |
| 240 | N/A | | | | | N/A | | Japanese | c.580+2T>C | - | NR | N/A | N/A | N/A | N/A | N/A | N/A | N/A | N/A | N/A | N/A | Y | N/A | N/A | N/A | 9 |
| 241 | N/A | | | | | N/A | | Japanese | c.580+2T>C | - |  | N/A | N/A | N/A | N/A | N/A | N/A | N/A | N/A | N/A | N/A | Y | N/A | N/A | N/A | 9 |
| 242 | N/A | | | | | N/A | | Japanese | c.580+3delGTAA | - | NR | N/A | N/A | Y | N/A | N/A | N/A | N/A | N/A | N/A | N/A | Y | N/A | N/A | N/A | 9 |
| 243 | N/A | | | | | N/A | | Japanese | c.580+3delGTAA | - |  | N/A | N/A | Y | N/A | N/A | N/A | N/A | N/A | N/A | N/A | Y | N/A | N/A | N/A | 9 |
| 244 | Female | | | | | 13 | | Chinese | c.581–9T>G | - | NR | Maternal | Y | N/A | Y | Y | N/A | Y | Y | N/A | Y | Y | N/A | N/A | N/A | 69 |
| 245 | Female | | | | | 43 | | Chinese | c.581–9T>G | - |  | Maternal | Y | N/A | N | Y | N/A | Y | Y | N/A | Y | Y | N/A | N/A | N/A | 69 |
| 246 | Female | | | | | 47 | | Chinese | c.581–9T>G | - |  | Maternal | Y | N/A | Y | Y | N/A | Y | Y | N/A | Y | Y | N/A | N/A | N/A | 69 |
| 247 | Female | | | | | 51 | | Chinese | c.581–9T>G | - |  | Maternal | Y | N/A | N | Y | N/A | Y | Y | N/A | Y | Y | N/A | N/A | N/A | 69 |
| 248 | Female | | | | | 76 | | Chinese | c.581–9T>G | - |  | N/A | Y | N/A | Y | Y | N/A | Y | Y | N/A | Y | Y | N/A | N/A | N/A | 69 |
| 249 | N/A | | | | | N/A | | Japanese | c.590T>C | p.Phe197Ser | Pathogenic | N/A | N/A | N/A | N/A | N/A | N/A | N/A | N/A | N/A | N/A | N/A | N/A | N/A | N/A | 5 |
| 250 | N/A | | | | | N/A | | Japanese | c.590T>C | p.Phe197Ser |  | N/A | N/A | Y | N/A | N/A | N/A | N/A | N/A | N/A | N/A | Y | N/A | N/A | N/A | 9 |
| 251 | N/A | | | | | N/A | | Japanese | c.590T>C | p.Phe197Ser |  | N/A | N/A | Y | N/A | N/A | N/A | N/A | N/A | N/A | N/A | Y | N/A | N/A | N/A | 9 |
| 252 | N/A | | | | | N/A | | Japanese | c.590T>C | p.Phe197Ser |  | N/A | N/A | Y | N/A | N/A | N/A | N/A | N/A | N/A | N/A | Y | N/A | N/A | N/A | 9 |
| 253 | Male | | | | | 25 | | Chinese | c.592delA | p.Thr198Profs*2 | NR | Maternal | N/A | Y | Y | Y | N/A | Y | N/A | N/A | Y | Y | N/A | N/A | N/A | 6 |
| 254 | Female | | | | | 50 | | Chinese | c.592delA | p.Thr198Profs*2 |  | N/A | N/A | N/A | N/A | Y | N/A | Y | N/A | N/A | Y | N/A | N/A | N/A | N/A | 6 |
| 255 | Male | | | | | 2.8Y | | Japanese | c.593_601delCCTTGACCA | p.Thr198_Thr200del | NR | *De novo* | Y | Y | N/A | Y | N/A | N/A | N/A | N/A | N/A | Y | Y | Y | N/A | 70 |
| 256 | Male | | | | | 22 | | Turkish | c.594_595delCTinsG | p.Leu199* | Pathogenic | *De novo* | N/A | Y | Y | Y | N/A | Y | N/A | Y | Y | Y | Y | Y | N/A | 2 |
| 257 | Female | | | | | 18 | | N/A | c.596G>A | p.Arg190Gln | NR | N/A | Y | Y | Y | Y | N/A | Y | N/A | Y | Y | Y | Y | Y | N/A | 71 |
| 258 | Male | | | | | 17 | | N/A | c.596G>A | p.Arg190Gln |  | N/A | Y | Y | Y | Y | N/A | Y | N/A | Y | Y | Y | Y | Y | N/A | 71 |
| 259 | N/A | | | | | N/A | | N/A | c.597G>T | p.Leu199Phe | Likely pathogenic | *De novo* | N/A | Y | N/A | N/A | N/A | N/A | N/A | N/A | N/A | Y | N/A | N/A | N/A | 7 |
| 260 | Male | | | | | N/A | | N/A | c.598A>G | p.Thr200Ala | Pathogenic | Paternal | N/A | Y | N/A | N/A | N/A | N/A | N/A | N/A | Y | Y | N/A | N/A | N/A | 7 |
| 261 | Male | | | | | N/A | | N/A | c.598A>G | p.Thr200Ala |  | Paternal | N/A | Y | N/A | N/A | N/A | N/A | N/A | N/A | Y | Y | N/A | N/A | N/A | 7 |
| 262 | Male | | | | | N/A | | N/A | c.598A>G | p.Thr200Ala |  | N/A | N/A | N | N/A | N/A | N/A | N/A | N/A | N/A | Y | N | N/A | N/A | N/A | 7 |
| 263 | Female | | | | | N/A | | Chinese | c.599C>T | p.Thr200Ile | NR | *De novo* | N/A | Y | N/A | N/A | N/A | N/A | N/A | N/A | Y | Y | N/A | N/A | N/A | 72 |
| 264 | N/A | | | | | N/A | | N/A | c.599C>T | p.Thr200Ile |  | N/A | N/A | N/A | N/A | N/A | N/A | N/A | N/A | N/A | N/A | N/A | N/A | N/A | N/A | 14 |
| 265 | Female | | | | | 16 | | Chinese | c.599C>T | p.Thr200Ile |  | *De novo* | N | Y | N/A | N/A | N/A | N/A | N/A | N/A | Y | Y | N/A | N/A | N/A | 44 |
| 266 | N/A | | | | | N/A | | Caucasian | c.604delA | p.Thr202Profs*9 | Pathogenic | N/A | N/A | Y | N/A | N/A | N/A | N/A | N/A | N/A | Y | Y | N/A | N/A | N/A | 24 |
| 267 | N/A | | | | | N/A | | Caucasian | c.604delA | p.Thr202Profs*9 |  | N/A | N/A | Y | N/A | N/A | N/A | N/A | N/A | N/A | Y | Y | N/A | N/A | N/A | 24 |
| 268 | N/A | | | | | N/A | | Chinese | c.610A>T | p.Lys 204* | NR | N/A | N/A | N/A | N/A | N/A | N/A | N/A | N/A | N/A | N/A | N/A | N/A | N/A | N/A | 26 |
| 269 | N/A | | | | | N/A | | N/A | c.614C>G | p.Thr205Arg | NR | N/A | N/A | N/A | N/A | N/A | N/A | N/A | N/A | N/A | N/A | N/A | N/A | N/A | N/A | 13 |
| 270 | Male | | | | | 10.5 | | Turkish | c.614C>G | p.Thr205Arg |  | Familial | Y | Y | Y | Y | N/A | Y | N/A | Y | Y | Y | Y | N | N/A | 2 |
| 271 | Female | | | | | 10.5 | | Turkish | c.614C>G | p.Thr205Arg |  | Familial | N | Y | Y | Y | N/A | Y | N/A | Y | Y | Y | Y | N | N/A | 2 |
| 272 | Female | | | | | 42 | | Turkish | c.614C>G | p.Thr205Arg |  | Familial | Y | Y | Y | Y | N/A | Y | N/A | Y | Y | Y | Y | N | N/A | 2 |
| 273 | Male | | | | | 10.5 | | Turkish | c.614C>G | p.Thr205Arg |  | Familial | Y | Y | Y | Y | N/A | Y | N/A | Y | Y | Y | Y | N | N/A | 2 |
| 274 | N/A | | | | | N/A | | N/A | c.624_625dup | p.Gln209Profs*3 | NR | N/A | N/A | N/A | N/A | N/A | N/A | N/A | N/A | N/A | N/A | N/A | N/A | N/A | N/A | 14 |
| 275 | Male | | | | | 12 | | N/A | c.625C>T | p.Gln209* | Pathogenic | N/A | N/A | Y | N/A | N/A | N/A | N/A | N/A | N | Y | Y | N/A | N/A | N/A | 73 |
| 276 | N/A | | | | | N/A | | N/A | c.626A>G | p.Gln209Arg | NR | *De novo* | N/A | Y | N/A | N/A | N/A | N/A | N/A | N/A | N/A | Y | N/A | N/A | N/A | 7 |
| 277 | N/A | | | | | N/A | | N/A | c.627A>T | p.Gln209His | NR | N/A | N/A | N/A | N/A | N/A | N/A | N/A | N/A | N/A | N/A | N/A | N/A | N/A | N/A | 14 |
| 278 | Male | | | | | 17 | | Japanese | c.628C>T | p.Pro224Ser | NR | Paternal | Y | Y | Y | N/A | N/A | N/A | N/A | N/A | Y | N | N/A | N/A | N/A | 74 |
| 279 | Female | | | 15 | | | | Japanese | c.628C>T | p.Pro224Ser |  | Paternal | Y | Y | Y | N/A | N/A | N/A | N/A | N/A | Y | N | N/A | N/A | N/A | 74 |
| 280 | Male | | | 12 | | | | Japanese | c.628C>T | p.Pro224Ser |  | Paternal | Y | Y | Y | N/A | N/A | N/A | N/A | N/A | Y | N | N/A | N/A | N/A | 74 |
| 281 | Male | | | 52 | | | | Japanese | c.628C>T | p.Pro224Ser |  | *De novo* | N/A | N/A | N/A | N/A | N/A | N/A | N/A | N/A | Y | N/A | N/A | N/A | N/A | 74 |
| 282 | N/A | | | | | | N/A | N/A | c.631G>C | p.Ala211Pro | Uncertain significance | N/A | N/A | N/A | N/A | N/A | N/A | N/A | N/A | N/A | N/A | N/A | N/A | N/A | N/A | 14 |
| 283 | N/A | | | | | | N/A | N/A | c.631_633dup | p.Ala211dup | NR | N/A | N/A | N/A | N/A | N/A | N/A | N/A | N/A | N/A | N/A | N/A | N/A | N/A | N/A | 14 |
| 284 | Male | | | | | | 11 | Chinese | c.634T>G | p.Thr212Pro | NR | Maternal | N/A | N/A | N/A | N/A | N/A | N/A | N/A | N/A | Y | Y | N/A | N/A | N/A | 75 |
| 285 | Female | | | | | | N/A | Chinese | c.634T>G | p.Thr212Pro |  | N/A | N/A | N/A | N/A | N/A | N/A | N/A | N/A | N/A | N/A | N/A | N/A | N/A | N/A | 75 |
| 286 | Female | | | | | | 29 | Chinese | c.635_638delCCTA | p.Thr212Ilefs*9 | NR | Paternal | N/A | Y | Y | N/A | N/A | N/A | Y | N/A | Y | Y | Y | Y | N/A | 76 |
| 287 | Male | | | | | | N/A | Chinese | c.635_638delCCTA | p.Thr212Ilefs*9 |  | Maternal | N/A | Y | N/A | N/A | N/A | N/A | N/A | N/A | N/A | Y | N/A | N/A | N/A | 76 |
| 288 | Male | | | | | | N/A | Chinese | c.635_638delCCTA | p.Thr212Ilefs*9 |  | Paternal | N/A | Y | N/A | N/A | N/A | N/A | N/A | N/A | N/A | Y | N/A | N/A | N/A | 76 |
| 289 | Female | | | | | | N/A | Chinese | c.635_638delCCTA | p.Thr212Ilefs*9 |  | Paternal | N/A | Y | N/A | N/A | N/A | N/A | N/A | N/A | N/A | Y | N/A | N/A | N/A | 76 |
| 290 | N/A | | | | | | N/A | N/A | c.636delC | p.Tyr213Ilefs*9 | NR | *De novo* | N/A | Y | N/A | N/A | N/A | N/A | N/A | N/A | N/A | Y | N/A | N/A | N/A | 7 |
| 291 | Female | | | | | | 50 | Turkish | c.636dupC | p.Tyr213Leufs*6 | Pathogenic | Familial | N/A | Y | Y | Y | N/A | N | N/A | N | Y | Y | N | Y | N/A | 2 |
| 292 | Male | | | | | | 27 | Turkish | c.636dupC | p.Tyr213Leufs*6 |  | Familial | N/A | Y | Y | Y | N/A | Y | N/A | Y | Y | Y | Y | Y | N/A | 2 |
| 293 | Female | | | | | | 14 | Turkish | c.636dupC | p.Tyr213Leufs*6 |  | Familial | N/A | Y | Y | Y | N/A | Y | N/A | N | Y | Y | N | N | N/A | 2 |
| 294 | Female | | | | | | 13 | Turkish | c.636dupC | p.Tyr213Leufs*6 |  | Familial | N/A | Y | Y | Y | N/A | Y | N/A | N | Y | Y | Y | Y | N/A | 2 |
| 295 | Male | | | | | | N/A | Chinese | c.644delG | p.Arg215Lysfs*7 | NR | *De novo* | N/A | Y | Y | N/A | N/A | N/A | N/A | N/A | Y | Y | N/A | N/A | N/A | 72 |
| 296 | Male | | | | | | 15 | Chinese | c.644delG | p.Arg215Lysfs*7 |  | *De novo* | Y | Y | N/A | N/A | N/A | N/A | N/A | N/A | Y | Y | N/A | N/A | N/A | 44 |
| 297 | Male | | | | | | 12 | Mexican | c.651_652delTA | p.Lys218Serfs*17 | NR | Maternal | N/A | Y | Y | Y | N/A | Y | Y | N/A | Y | Y | Y | N | N/A | 77 |
| 298 | N/A | | | | | | N/A | N/A | c.652A>C | p.Lys218Gln | Likely pathogenic | N/A | N/A | N/A | N/A | N/A | N/A | N/A | N/A | N/A | N/A | N/A | N/A | N/A | N/A | 14 |
| 299 | N/A | | | | | | N/A | N/A | c.652A>G | p.Lys218Glu | Pathogenic | N/A | N/A | N/A | N/A | N/A | N/A | N/A | N/A | N/A | N/A | N/A | N/A | N/A | N/A | 14 |
| 300 | N/A | | | | | | N/A | Polish | c.652A>G | p.Lys218Glu |  | *De novo* | Y | Y | Y | N/A | N | N/A | N/A | N/A | Y | N | N/A | Y | N/A | 30 |
| 301 | N/A | | | | | | N/A | Polish | c.652A>G | p.Lys218Glu |  | Familial | Y | Y | Y | N/A | N | N/A | N/A | N/A | Y | Y | N/A | Y | N/A | 30 |
| 302 | N/A | | | | | | N/A | Japanese | c.654A>T | p.Lys218Asn | NR | N/A | N/A | Y | N/A | N/A | N/A | N/A | N/A | N/A | N/A | Y | N/A | N/A | N/A | 9 |
| 303 | N/A | | | | | | N/A | Japanese | c.654A>T | p.Lys218Asn |  | N/A | N/A | Y | N/A | N/A | N/A | N/A | N/A | N/A | N/A | Y | N/A | N/A | N/A | 9 |
| 304 | N/A | | | | | | N/A | Japanese | c.654A>T | p.Lys218Asn |  | N/A | N/A | Y | N/A | N/A | N/A | N/A | N/A | N/A | N/A | Y | N/A | N/A | N/A | 9 |
| 305 | N/A | | | | | | N/A | Japanese | c.654A>T | p.Lys218Asn |  | N/A | N/A | Y | N/A | N/A | N/A | N/A | N/A | N/A | N/A | Y | N/A | N/A | N/A | 9 |
| 306 | N/A | | | | | | N/A | Japanese | c.659C>T | p.Thr220Ile | Pathogenic | N/A | N/A | Y | N/A | N/A | N/A | N/A | N/A | N/A | N/A | Y | N/A | N/A | N/A | 9 |
| 307 | N/A | | | | | | N/A | Japanese | c.659C>T | p.Thr220Ile |  | N/A | N/A | Y | N/A | N/A | N/A | N/A | N/A | N/A | N/A | Y | N/A | N/A | N/A | 9 |
| 308 | N/A | | | | | | N/A | Japanese | c.659C>T | p.Thr220Ile |  | N/A | N/A | Y | N/A | N/A | N/A | N/A | N/A | N/A | N/A | Y | N/A | N/A | N/A | 9 |
| 309 | Male | | | | | | 5Y 6M | Polish | c.659C>G | p.Thr220Arg | NR | *De novo* | Y | Y | Y | N/A | Y | N/A | N/A | N/A | Y | Y | N/A | Y | N/A | 30 |
| 310 | Female | | | | | | 29 | Mexican | c.662T>A | p.Val221Glu | NR | Maternal | Y | N/A | Y | Y | N/A | Y | Y | N/A | Y | Y | N/A | Y | N/A | 77 |
| 311 | Female | | | | | | N/A | Italian | c.662T>G | p.Val221Gly | NR | Maternal | N/A | N/A | N/A | N/A | N/A | N/A | N/A | N/A | N/A | N/A | N/A | N/A | N/A | 4 |
| 312 | Female | | | | | | N/A | Italian | c.662T>G | p.Val221Gly |  | N/A | N/A | N/A | N/A | N/A | N/A | N/A | N/A | N/A | N/A | N/A | N/A | N/A | N/A | 4 |
| 313 | Female | | | | | | N/A | Italian | c.662T>G | p.Val221Gly |  | N/A | N/A | N/A | N/A | N/A | N/A | N/A | N/A | N/A | N/A | N/A | N/A | N/A | N/A | 4 |
| 314 | N/A | | | | | | N/A | N/A | c.667G>A | p.Gly223Arg | NR | N/A | N/A | N/A | N/A | N/A | N/A | N/A | N/A | N/A | N/A | N/A | N/A | N/A | N/A | 14 |
| 315 | Male | | | | | | 6 | Chinese | c.668G>T | p.Gly223Val | NR | Maternal | Y | Y | Y | Y | N/A | Y | Y | N/A | Y | Y | Y | Y | Y | 18 |
| 316 | Female | | | | | | N/A | Chinese | c.668G>T | p.Gly223Val |  | Maternal | N/A | N/A | N/A | N/A | N/A | N/A | N/A | N/A | N/A | N/A | N/A | N/A | N/A | 18 |
| 317 | Male | | | | | | N/A | Chinese | c.668G>T | p.Gly223Val |  | Maternal | N/A | N/A | N/A | N/A | N/A | N/A | N/A | N/A | N/A | N/A | N/A | N/A | N/A | 18 |
| 318 | Female | | | | | | N/A | Chinese | c.668G>T | p.Gly223Val |  | N/A | N/A | N/A | N/A | N/A | N/A | N/A | N/A | N/A | N/A | N/A | N/A | N/A | N/A | 18 |
| 319 | N/A | | | | | | N/A | Japanese | c.673C>T | p.Arg225Trp | Pathogenic | N/A | N/A | Y | N/A | N/A | N/A | N/A | N/A | N/A | Y | Y | N/A | N/A | N/A | 9 |
| 320 | N/A | | | | | | N/A | Japanese | c.673C>T | p.Arg225Trp |  | N/A | N/A | Y | N/A | N/A | N/A | N/A | N/A | N/A | Y | Y | N/A | N/A | N/A | 9 |
| 321 | Male | | | | | | 75 | Japanese | c.673C>T | p.Arg225Trp |  | *De novo* | Y | N/A | Y | Y | N/A | Y | N/A | N/A | N/A | Y | Y | Y | N/A | 78 |
| 322 | N/A | | | | | | N/A | Italian | c.673C>T | p.Arg225Trp |  | Familial | N/A | N/A | N/A | N/A | N/A | N/A | N/A | N/A | N/A | N/A | N/A | N/A | N/A | 4 |
| 323 | Female | | | | | | 11 | Caucasian | c.673C>T | p.Arg225Trp |  | *De novo* | N/A | N/A | Y | Y | N/A | N/A | N/A | N/A | Y | N/A | N/A | N/A | N/A | 12 |
| 324 | N/A | | | | | | N/A | N/A | c.673C>T | p.Arg225Trp |  | N/A | N/A | N/A | N/A | N/A | N/A | N/A | N/A | N/A | N/A | N/A | N/A | N/A | N/A | 13 |
| 325 | Male | | | | | | 15 | Chinese | c.673C>T | p.Arg225Trp |  | N/A | N/A | Y | N/A | Y | N/A | N/A | N/A | Y | Y | Y | N/A | Y | N/A | 38 |
| 326 | Female | | | | | | 10Y 10M | Korean | c.673C>T | p.Arg225Trp |  | *De novo* | Y | N/A | N/A | Y | N/A | N/A | N/A | N/A | Y | Y | N/A | N/A | N/A | 23 |
| 327 | Male | | | | | | 12 | Chinese | c.673C>T | p.Arg225Trp |  | *De novo* | Y | Y | Y | N/A | N/A | N/A | N/A | N/A | Y | Y | N/A | N/A | N/A | 15 |
| 328 | N/A | | | | | | N/A | Polish | c.673C>T | p.Arg225Trp |  | *De novo* | Y | Y | Y | N/A | N | N/A | N/A | N/A | Y | N | N/A | Y | N/A | 30 |
| 329 | N/A | | | | | | N/A | Polish | c.673C>T | p.Arg225Trp |  | *De novo* | Y | Y | Y | N/A | N | N/A | N/A | N/A | Y | Y | N/A | Y | N/A | 30 |
| 330 | Male | | | | | | 9Y11M | Chinese | c.673C>T | p.Arg225Trp |  | *De novo* | Y | Y | Y | Y | N/A | Y | Y | N/A | Y | Y | Y | Y | Y | 18 |
| 331 | Female | | | | | | 20 | Thai | c.673C>T | p.Arg225Trp |  | *De novo* | N/A | Y | Y | N/A | N/A | N/A | N/A | Y | Y | Y | N/A | N/A | N/A | 80 |
| 332 | N/A | | | N/A | | | | N/A | c.674G>A | p.Arg225Gln | Pathogenic | N/A | N/A | N/A | N/A | N/A | N/A | N/A | N/A | N/A | N/A | N/A | N/A | N/A | N/A | 13 |
| 333 | N/A | | | N/A | | | | N/A | c.674G>A | p.Arg225Gln |  | N/A | N/A | N/A | N/A | N/A | N/A | N/A | N/A | N/A | N/A | N/A | N/A | N/A | N/A | 13 |
| 334 | N/A | | | N/A | | | | N/A | c.674G>A | p.Arg225Gln |  | N/A | N/A | N/A | N/A | N/A | N/A | N/A | N/A | N/A | N/A | N/A | N/A | N/A | N/A | 13 |
| 335 | N/A | | | N/A | | | | N/A | c.674G>A | p.Arg225Gln |  | *De novo* | N/A | Y | N/A | N/A | N/A | N/A | N/A | N/A | N/A | Y | N/A | N/A | N/A | 7 |
| 336 | N/A | | | N/A | | | | Japanese | c.674G>A | p.Arg225Gln |  | N/A | N/A | Y | N/A | N/A | N/A | N/A | N/A | N/A | Y | Y | N/A | N/A | N/A | 9 |
| 337 | Female | | | 36 | | | | N/A | c.674G>A | p.Arg225Gln |  | *De novo* | N/A | N/A | N/A | N/A | N/A | N/A | N/A | N/A | N/A | N/A | N/A | N/A | N/A | 127 |
| 338 | Male | | | 7 | | | | Caucasian | c.674G>A | p.Arg225Gln |  | Familial | N/A | N/A | Y | Y | N/A | N/A | N/A | N/A | Y | N/A | N/A | N/A | N/A | 12 |
| 339 | Male | | | 9 | | | | Caucasian | c.674G>A | p.Arg225Gln |  | Familial | N/A | N/A | N | Y | N/A | N/A | N/A | N/A | Y | N/A | N/A | N/A | N/A | 12 |
| 340 | Female | | | 29 | | | | Caucasian | c.674G>A | p.Arg225Gln |  | Familial | N/A | N/A | Y | Y | N/A | N/A | N/A | N/A | Y | N/A | N/A | N/A | N/A | 12 |
| 341 | Female | | | 19 | | | | Caucasian | c.674G>A | p.Arg225Gln |  | N/A | N/A | N/A | Y | Y | N/A | N/A | N/A | N/A | Y | N/A | N/A | N/A | N/A | 12 |
| 342 | N/A | | | N/A | | | | Caucasian | c.674G>A | p.Arg225Gln |  | N/A | N/A | Y | N/A | N/A | N/A | N/A | N/A | N/A | Y | Y | N/A | N/A | N/A | 24 |
| 343 | Female | | | 28 | | | | Korean | c.674G>A | p.Arg225Gln |  | Familial | N/A | N/A | N/A | N/A | N/A | N/A | N/A | N/A | Y | N/A | N/A | N/A | N/A | 3 |
| 344 | Female | | | 17 | | | | Korean | c.674G>A | p.Arg225Gln |  | *De novo* | N/A | N/A | N/A | N/A | N/A | N/A | N/A | N/A | Y | N/A | N/A | N/A | N/A | 3 |
| 345 | Female | | | 28 | | | | N/A | c.674G>A | p.Arg225Trp |  | *De novo* | N | Y | N/A | N/A | N | Y | N/A | N/A | Y | Y | Y | Y | N/A | 126 |
| 346 | Male | | | 8 | | | | N/A | c.674G>A | p.Arg225Trp |  | Maternal | N/A | Y | N/A | N/A | N | Y | N/A | N/A | Y | Y | Y | N/A | N/A | 126 |
| 347 | Male | | | 4 | | | | N/A | c.674G>A | p.Arg225Trp |  | Maternal | N/A | Y | N/A | N/A | N | Y | N/A | N/A | Y | Y | Y | N/A | N/A | 126 |
| 348 | Male | | | 29 | | | | Chinese | c.674G>A | p.Arg225Gln |  | Maternal | N/A | Y | N/A | N/A | N/A | N/A | N/A | Y | Y | Y | N/A | N/A | N/A | 38 |
| 349 | Female | | | N/A | | | | Chinese | c.674G>A | p.Arg225Gln |  | N/A | N/A | N/A | N/A | N/A | N/A | N/A | N/A | N/A | N/A | N/A | N/A | N/A | N/A | 38 |
| 350 | Male | | | N/A | | | | Chinese | c.674G>A | p.Arg225Gln |  | Maternal | N/A | N/A | N/A | N/A | N/A | N/A | N/A | N/A | N/A | N/A | N/A | N/A | N/A | 38 |
| 351 | Female | | | 50 | | | | Japanese | c.674G>A | p.Arg225Gln |  | N/A | N/A | N/A | N/A | N/A | N/A | N/A | N/A | N/A | Y | N/A | N/A | N/A | N/A | 29 |
| 352 | Male | | | 11 | | | | Japanese | c.674G>A | p.Arg225Gln |  | Maternal | N/A | N/A | N/A | N/A | N/A | N/A | N/A | N/A | Y | N/A | N/A | N/A | N/A | 29 |
| 353 | Female | | | 10 | | | | Japanese | c.674G>A | p.Arg225Gln |  | N/A | Y | Y | N/A | N/A | N/A | N/A | N/A | N/A | Y | Y | N/A | N/A | N/A | 29 |
| 354 | Female | | | 10 | | | | Japanese | c.674G>A | p.Arg225Gln |  | N/A | Y | Y | N/A | N/A | N/A | N/A | N/A | N/A | Y | Y | N/A | N/A | N/A | 29 |
| 355 | N/A | | | N/A | | | | Danish | c.674G>A | p.Arg225Gln |  | Familial | N/A | N/A | N/A | N/A | N/A | N/A | N/A | N/A | Y | N/A | N/A | N/A | N/A | 25 |
| 356 | N/A | | | N/A | | | | Danish | c.674G>A | p.Arg225Gln |  | Familial | Y | N/A | N/A | N/A | N/A | N/A | N/A | N/A | Y | N/A | N/A | N/A | N/A | 25 |
| 357 | N/A | | | N/A | | | | Danish | c.674G>A | p.Arg225Gln |  | Familial | Y | N/A | N/A | N/A | N/A | N/A | N/A | N/A | Y | N/A | N/A | N/A | N/A | 25 |
| 358 | N/A | | | N/A | | | | Danish | c.674G>A | p.Arg225Gln |  | Familial | N | N/A | Y | N/A | N/A | N/A | N/A | N/A | Y | N/A | N/A | N/A | N/A | 25 |
| 359 | N/A | | | N/A | | | | Danish | c.674G>A | p.Arg225Gln |  | Familial | Y | N/A | Y | N/A | N/A | N/A | N/A | N/A | Y | N/A | N/A | N/A | N/A | 25 |
| 360 | N/A | | | N/A | | | | Danish | c.674G>A | p.Arg225Gln |  | Familial | N/A | N/A | N/A | N/A | N/A | N/A | N/A | N/A | N/A | N/A | N/A | N/A | N/A | 25 |
| 361 | Female | | | 8 | | | | Taiwanese | c.674G>A | p.Arg225Gln |  | *De novo* | N | N/A | Y | N/A | N/A | N/A | N/A | N/A | Y | Y | Y | N/A | N/A | 53 |
| 362 | Male | | | 19 | | | | N/A | c.674G>A | p.Arg225Gln |  | *De novo* | Y | Y | Y | Y | N/A | Y | N/A | N | Y | Y | N | N | N/A | 71 |
| 363 | Male | | | 46 | | | | N/A | c.674G>A | p.Arg225Gln |  | Paternal | Y | Y | Y | Y | N/A | Y | N/A | Y | Y | Y | N | Y | N/A | 71 |
| 364 | Female | | | 15 | | | | N/A | c.674G>A | p.Arg225Gln |  | N/A | Y | Y | Y | Y | N/A | Y | N/A | Y | Y | Y | N | Y | N/A | 71 |
| 365 | Male | | | 17 | | | | N/A | c.674G>A | p.Arg225Gln |  | *De novo* | N/A | N/A | N/A | Y | N/A | N/A | N/A | N/A | Y | Y | N/A | N/A | N/A | 81 |
| 366 | Male | | | 8 | | | | Chinese | c.674G>A | p.Arg225Gln |  | Maternal | Y | Y | Y | Y | N/A | N/A | N/A | Y | Y | Y | N/A | N/A | N/A | 82 |
| 367 | Male | | | 33 | | | | Chinese | c.674G>A | p.Arg225Gln |  | Maternal | Y | Y | Y | Y | N/A | N/A | N/A | Y | Y | Y | N/A | N/A | N/A | 82 |
| 368 | Female | | | 58 | | | | Chinese | c.674G>A | p.Arg225Gln |  | *De novo* | Y | Y | Y | Y | N/A | N/A | N/A | Y | Y | Y | N/A | N/A | N/A | 82 |
| 369 | Female | | | 35 | | | | Chinese | c.674G>A | p.Arg225Gln |  | Maternal | Y | Y | Y | Y | N/A | N/A | N/A | Y | Y | Y | N/A | N/A | N/A | 82 |
| 370 | Male | | | 23 | | | | Chinese | c.674G>A | p.Arg225Gln |  | *De novo* | Y | Y | Y | N/A | N/A | Y | Y | Y | Y | Y | N/A | N/A | N/A | 125 |
| 371 | Male | | | 58 | | | | N/A | c.674G>A | p.Arg225Gln |  | Paternal | Y | N/A | Y | Y | N/A | Y | Y | Y | N/A | Y | Y | N/A | N/A | 83 |
| 372 | Female | | | 39 | | | | N/A | c.674G>A | p.Arg225Gln |  | Familial | N/A | Y | N/A | N/A | N/A | N/A | N/A | N/A | Y | Y | N/A | N/A | N/A | 64 |
| 373 | Female | | | 34 | | | | N/A | c.674G>A | p.Arg225Gln |  | Familial | N/A | Y | N/A | N/A | N/A | N/A | N/A | N/A | Y | Y | N/A | N/A | N/A | 64 |
| 374 | Female | | | 16 | | | | N/A | c.674G>A | p.Arg225Gln |  | Maternal | N/A | Y | N/A | N/A | N/A | N/A | N/A | N/A | Y | Y | N/A | N/A | N/A | 64 |
| 375 | Male | | | 9 | | | | N/A | c.674G>A | p.Arg225Gln |  | Maternal | N/A | Y | N/A | N/A | N/A | N/A | N/A | N/A | Y | Y | N/A | N/A | N/A | 64 |
| 376 | Male | | | 3 | | | | Colombia | c.674G>A | p.Arg225Gln |  | *De novo* | N/A | Y | N/A | N/A | N/A | N/A | N/A | N | N/A | Y | Y | N | N/A | 84 |
| 377 | Female | | | N/A | | | | Korean | c.674G>A | p.Arg225Gln |  | N/A | N/A | N/A | N/A | N/A | N/A | N/A | N/A | N/A | N/A | N/A | N/A | N/A | N/A | 46 |
| 378 | Female | | | N/A | | | | Korean | c.674G>A | p.Arg225Gln |  | N/A | N/A | N/A | N/A | N/A | N/A | N/A | N/A | N/A | N/A | N/A | N/A | N/A | N/A | 46 |
| 379 | Male | | | 13 | | | | N/A | c.674G>A | p.Arg225Gln |  | *De novo* | N | Y | Y | Y | N | Y | N/A | N/A | Y | Y | N | N/A | N/A | 39 |
| 380 | Male | | | 37 | | | | N/A | c.674G>A | p.Arg225Gln |  | N/A | Y | Y | Y | Y | N | Y | N/A | N/A | Y | Y | N | N/A | N/A | 39 |
| 381 | Female | | | 11 | | | | N/A | c.674G>A | p.Arg225Gln |  | Maternal | N | N | Y | Y | N | Y | N/A | N/A | Y | Y | N | N/A | N/A | 39 |
| 382 | Female | | | 2 | | | | N/A | c.674G>A | p.Arg225Gln |  | Maternal | N | Y | Y | Y | N | Y | N/A | N/A | N/A | Y | Y | N/A | N/A | 39 |
| 383 | Male | | | 15 | | | | Italian | c.674G>A | p.Arg225Gln |  | Familial | Y | Y | Y | Y | N/A | Y | N/A | N/A | Y | Y | Y | N/A | N/A | 85 |
| 384 | Male | | | 13 | | | | Italian | c.674G>A | p.Arg225Gln |  | Familial | Y | Y | Y | Y | N/A | Y | N/A | N/A | Y | Y | Y | N/A | N/A | 85 |
| 385 | Male | | | 12 | | | | Chinese | c.674G>A | p.Arg225Gln |  | Paternal | N/A | N/A | N/A | N/A | N/A | N/A | N/A | N/A | Y | N/A | N/A | N/A | N/A | 86 |
| 386 | Male | | | 4y | | | | Korean | c.674G>A | p.Arg225Gln |  | De novo | N/A | Y | N/A | N/A | N/A | N/A | N/A | N/A | Y | Y | N/A | N/A | N/A | 87 |
| 387 | Female | | | 16M | | | | Turkish | c.674G>A | p.Arg225Gln |  | *De novo* | Y | Y | Y | Y | N/A | Y | N/A | N | Y | Y | Y | N | N/A | 2 |
| 388 | Male | | | 3.5 | | | | Turkish | c.674G>A | p.Arg225Gln |  | *De novo* | N | Y | Y | Y | N/A | Y | N/A | N | Y | Y | Y | Y | N/A | 2 |
| 389 | Female | | | 9 | | | | Turkish | c.674G>A | p.Arg225Gln |  | Familial | N/A | Y | Y | Y | N/A | Y | N/A | Y | Y | Y | Y | Y | N/A | 2 |
| 390 | Female | | | 29 | | | | Turkish | c.674G>A | p.Arg225Gln |  | Familial | N/A | Y | N | Y | N/A | N | N/A | N/A | Y | N/A | Y | N | N/A | 2 |
| 391 | Male | | | 13 | | | | Turkish | c.674G>A | p.Arg225Gln |  | *De novo* | N/A | Y | Y | Y | N/A | Y | N/A | Y | Y | Y | Y | N | N/A | 2 |
| 392 | Female | | | 30 | | | | Turkish | c.674G>A | p.Arg225Gln |  | N/A | Y | Y | Y | Y | N | Y | Y | N/A | Y | Y | Y | Y | N/A | 88 |
| 393 | Female | | | 22 | | | | N/A | c.674G>A | p.Arg225Gln |  | Paternal | Y | Y | Y | Y | N/A | N/A | Y | N | Y | N/A | N/A | Y | N/A | 89 |
| 394 | Male | | | 3 | | | | Thai | c.674G>A | p.Arg225Gln |  | *De novo* | N/A | Y | Y | N/A | N/A | N/A | N/A | N/A | N/A | Y | Y | N/A | N/A | 80 |
| 395 | Male | | | 7 | | | | Mexican | c.674G>A | p.Arg225Gln |  | *De novo* | Y | Y | Y | Y | N/A | Y | Y | N/A | Y | Y | Y | N | N | 77 |
| 396 | Male | | | Newborn | | | | Mongolian | c.674G>A | p.Arg225Gln |  | N/A | N/A | Y | N/A | N/A | N/A | Y | Y | N/A | N/A | N/A | Y | N/A | N/A | 57 |
| 397 | Male | | | Fetus | | | | Chinese | c.674G>A | p.Arg225Gln |  | N/A | N/A | Y | N/A | N/A | N/A | N/A | Y | N/A | N/A | Y | Y | N/A | N/A | 91 |
| 398 | N/A | | | Fetus | | | | Chinese | c.674G>T | p.Arg225Leu | not provided | De novo | N/A | Y | N/A | N/A | N/A | N/A | Y | N/A | N/A | Y | N/A | N/A | N/A | 63 |
| 399 | Female | | | N/A | | | | Chinese | c.674G>T | p.Arg225Leu |  | Paternal | N/A | Y | Y | Y | N/A | N/A | Y | N/A | Y | Y | N/A | Y | N/A | 72 |
| 400 | Male | | | N/A | | | | Chinese | c.674G>T | p.Arg225Leu |  | N/A | N | Y | N/A | N/A | N/A | N/A | N/A | N/A | Y | Y | N/A | N/A | N/A | 72 |
| 401 | Male | | | N/A | | | | Chinese | c.674G>T | p.Arg225Leu |  | Paternal | Y | Y | N/A | N/A | N/A | N/A | N/A | N/A | Y | Y | Y | N/A | N/A | 72 |
| 402 | N/A | | | N/A | | | | N/A | c.674G>T | p.Arg225Leu |  | N/A | N/A | N/A | N/A | N/A | N/A | N/A | N/A | N/A | N/A | N/A | N/A | N/A | N/A | 14 |
| 403 | Female | | | 16 | | | | Chinese | c.674G>T | p.Arg225Leu |  | Paternal | Y | Y | N/A | N/A | N/A | N/A | N/A | N/A | Y | Y | N/A | N/A | N/A | 44 |
| 404 | Male | | | N/A | | | | Chinese | c.674G>T | p.Arg225Leu |  | Paternal | N/A | N/A | N/A | N/A | N/A | N/A | N/A | N/A | N/A | N/A | N/A | N/A | N/A | 44 |
| 405 | Male | | | N/A | | | | Chinese | c.674G>T | p.Arg225Leu |  | *De novo* | N/A | N/A | N/A | N/A | N/A | N/A | N/A | N/A | N/A | N/A | N/A | N/A | N/A | 44 |
| 406 | N/A | | | N/A | | | | Danish | c.674G>T | p.Arg225Leu |  | *De novo* | N | N/A | Y | N/A | N/A | N/A | N/A | N/A | Y | N/A | N/A | N/A | N/A | 25 |
| 407 | N/A | | | N/A | | | | N/A | c.682A>G | p.Arg228Gly | Uncertain significance | N/A | N/A | N/A | N/A | N/A | N/A | N/A | N/A | N/A | N/A | N/A | N/A | N/A | N/A | 14 |
| 408 | Female | | | 11 | | | | Turkish | c.685+4delAAGT | - | NR | *De novo* | Y | Y | Y | Y | N | Y | N/A | Y | Y | Y | Y | Y | N/A | 88 |
| **409** | **Male** | | | **3Y9M** | | | | **Japanese** | **c.685+2T>G** | **-** | **NR** | ***De novo*** | **Y** | **Y** | **Y** | **Y** | **N** | **Y** | **Y** | **N** | **Y** | **Y** | **Y** | **N** | **N** | **This study** |
| 410 | Male | | | 24M | | | | Turkish | c.685+5G>A | - | Uncertain significance | *De novo* | Y | Y | Y | Y | N/A | Y | N/A | Y | Y | Y | Y | Y | N/A | 2 |
| 411 | Female | | | 18 | | | | Chinese | c.685+5G>A | - |  | *De novo* | Y | Y | Y | Y | N/A | Y | Y | N/A | Y | Y | Y | N/A | N/A | 92 |
| 412 | N/A | | | N/A | | | | N/A | c.694C>T | p.Gln232* | NR | N/A | N/A | N/A | N/A | N/A | N/A | N/A | N/A | N/A | N/A | N/A | N/A | N/A | N/A | 14 |
| 413 | Female | | | 25 | | | | Turkish | c.694C>T | p.Gln232* |  | *De novo* | N/A | N | Y | Y | N/A | N | N/A | N | Y | Y | Y | N | N/A | 2 |
| 414 | Male | | | 10.5 | | | | Turkish | c.694C>T | p.Gln232* |  | Familial | Y | Y | Y | Y | N/A | Y | N/A | Y | Y | Y | Y | Y | N/A | 2 |
| 415 | Female | | | 12.5 | | | | Turkish | c.694C>T | p.Gln232* |  | Familial | N/A | N | Y | Y | N/A | Y | N/A | Y | Y | Y | Y | N | N/A | 2 |
| 416 | Female | | | 7.5 | | | | Turkish | c.694C>T | p.Gln232* |  | Familial | N/A | Y | Y | Y | N/A | Y | N/A | Y | Y | Y | N | Y | N/A | 2 |
| 417 | Female | | | 5 | | | | Turkish | c.694C>T | p.Gln232* |  | Familial | N/A | Y | Y | Y | N/A | Y | N/A | Y | N | Y | N | Y | N/A | 2 |
| 418 | Female | | | 37 | | | | Turkish | c.694C>T | p.Gln232* |  | Familial | N/A | N/A | Y | Y | N/A | Y | N/A | N | Y | Y | Y | N | N/A | 2 |
| 419 | N/A | | | N/A | | | | Caucasian | c.716delC | p.Pro239Leufs*11 | NR | N/A | N/A | Y | N/A | N/A | N/A | N/A | N/A | N/A | Y | Y | N/A | N/A | N/A | 24 |
| 420 | N/A | | | N/A | | | | Caucasian | c.716delC | p.Pro239Leufs*11 |  | N/A | N/A | Y | N/A | N/A | N/A | N/A | N/A | N/A | Y | Y | N/A | N/A | N/A | 24 |
| 421 | N/A | | | N/A | | | | N/A | c.718_721del | p.Ser240Cysfs*9 | NR | N/A | N/A | N/A | N/A | N/A | N/A | N/A | N/A | N/A | N/A | N/A | N/A | N/A | N/A | 14 |
| 422 | Male | | | 28 | | | | Japanese | c.722delT | p.Leu241Cysfs*9 | NR | *De novo* | Y | Y | N/A | Y | N/A | N/A | N/A | Y | Y | Y | Y | Y | N/A | 93 |
| 423 | Male | | | 12 | | | | Chinese | c.722_725delTGTT | p.Leu241Serfs*8 | NR | *De novo* | Y | Y | Y | Y | N/A | Y | Y | N/A | Y | Y | Y | Y | Y | 18 |
| 424 | Female | | | 24 | | | | Thai | c.739delA | p.Ser247Valfs*3 | Pathogenic | Maternal | Y | Y | Y | N/A | N/A | N/A | N/A | Y | Y | Y | N/A | N/A | N/A | 80 |
| 425 | N/A | | | N/A | | | | Italian | c.751C>T | p.Arg251Cys | NR | N/A | N/A | N/A | N/A | N/A | N/A | N/A | N/A | N/A | N/A | N/A | N/A | N/A | N/A | 22 |
| 426 | Male | | | 32 | | | | N/A | c.774_779delAGTAGGinsGA | p.Val259Metfs*11 | NR | *De novo* | N/A | Y | N/A | N/A | N/A | N/A | N/A | N/A | Y | Y | N/A | N/A | N/A | 64 |
| 427 | Male | | | 62 | | | | N/A | c.821delC | p.Pro274Glnfs*34 | NR | N/A | N/A | Y | Y | N/A | N/A | Y | N/A | N/A | Y | N/A | N/A | Y | N/A | 50 |
| 428 | Female | | | 18 | | | | N/A | c.821delC | p.Pro274Glnfs*34 |  | Paternal | N/A | Y | Y | N/A | N/A | Y | N/A | N/A | Y | N | N/A | Y | N/A | 50 |
| 429 | N/A | | | N/A | | | | Danish | c.821_822delCA | p.Pro274Glnfs*4 | NR | Familial | N | N/A | N/A | N/A | N/A | N/A | N/A | N/A | N | N/A | N/A | N/A | N/A | 25 |
| 430 | N/A | | | N/A | | | | Danish | c.821_822delCA | p.Pro274Glnfs*4 |  | Familial | N | N/A | Y | N/A | N/A | N/A | N/A | N/A | Y | N/A | N/A | N/A | N/A | 25 |
| 431 | N/A | | | N/A | | | | N/A | c.824delG | p.Ser275Ilefs*33 | NR | N/A | N/A | N/A | N/A | N/A | N/A | N/A | N/A | N/A | N/A | N/A | N/A | N/A | N/A | 13 |
| 432 | N/A | | | | N/A | | | N/A | c.831delT | p.Phe277Leufs*31 | NR | N/A | N/A | N/A | N/A | N/A | N/A | N/A | N/A | N/A | N/A | N/A | N/A | N/A | N/A | 14 |
| 433 | N/A | | | | N/A | | | Japanese | c.838C>T | p.Gln280* | Pathogenic | N/A | N/A | Y | N/A | N/A | N/A | N/A | N/A | N/A | N/A | Y | N/A | N/A | N/A | 9 |
| 434 | N/A | | | | N/A | | | Japanese | c.838C>T | p.Gln280* |  | N/A | N/A | Y | N/A | N/A | N/A | N/A | N/A | N/A | N/A | Y | N/A | N/A | N/A | 9 |
| 435 | N/A | | | | N/A | | | Japanese | c.838C>T | p.Gln280* |  | N/A | N/A | Y | N/A | N/A | N/A | N/A | N/A | N/A | N/A | Y | N/A | N/A | N/A | 9 |
| 436 | N/A | | | | N/A | | | Japanese | c.838C>T | p.Gln280* |  | N/A | N/A | Y | N/A | N/A | N/A | N/A | N/A | N/A | N/A | Y | N/A | N/A | N/A | 9 |
| 437 | Female | | | | 3Y9M | | | Japanese | c.844C>T | p.Gln282* | NR | Maternal | Y | Y | Y | N/A | N/A | Y | N/A | N/A | N/A | Y | N/A | Y | N/A | 94 |
| 438 | Female | | | | N/A | | | Japanese | c.844C>T | p.Gln282* |  | N/A | N/A | Y | Y | N/A | N/A | Y | N/A | N/A | N/A | N/A | N/A | N/A | N/A | 94 |
| 439 | Male | | | | N/A | | | Japanese | c.844C>T | p.Gln282* |  | Maternal | N/A | Y | Y | N/A | N/A | Y | N/A | N/A | N/A | N/A | N/A | N/A | N/A | 94 |
| 440 | N/A | | | | N/A | | | N/A | c.859G>A | p.Asp287Asn | NR | N/A | N/A | N/A | N/A | N/A | N/A | N/A | N/A | N/A | N/A | N/A | N/A | N/A | N/A | 14 |
| 441 | N/A | | | | N/A | | | N/A | c.859+1G>T | - | NR | Familial | N/A | Y | N/A | N/A | N/A | N/A | N/A | N/A | N/A | Y | N/A | N/A | N/A | 7 |
| 442 | Male | | | | 14M | | | N/A | c.860-1G>C | - | NR | *De novo* | N/A | N/A | Y | N/A | N | Y | Y | N/A | N/A | Y | Y | N/A | N/A | 97 |
| 443 | N/A | | | | N/A | | | Caucasian | c.860-2A>C | - | NR | N/A | N/A | Y | N/A | N/A | N/A | N/A | N/A | N/A | Y | Y | N/A | N/A | N/A | 24 |
| 444 | Male | | | | 26 | | | Turkish | c.860-2A>C | - |  | *De novo* | N/A | Y | Y | Y | N/A | Y | N/A | N | Y | Y | Y | N | N/A | 2 |
| 445 | Male | | | | N/A | | | Iranian | c.860-2A>G | - | NR | Maternal | Y | N/A | Y | Y | N/A | Y | Y | N/A | Y | Y | Y | Y | N/A | 96 |
| 446 | Male | | | | N/A | | | Iranian | c.860-2A>G | - |  | Maternal | Y | N/A | Y | Y | N/A | Y | Y | N/A | Y | Y | Y | Y | N/A | 96 |
| 447 | Male | | | | N/A | | | Iranian | c.860-2A>G | - |  | Maternal | Y | N/A | Y | Y | N/A | Y | Y | N/A | Y | Y | Y | Y | N/A | 96 |
| 448 | Female | | | | N/A | | | Iranian | c.860-2A>G | - |  | Paternal | Y | N/A | Y | Y | N/A | Y | Y | N/A | Y | Y | Y | Y | N/A | 96 |
| 449 | Female | | | | N/A | | | Iranian | c.860-2A>G | - |  | Paternal | Y | N/A | Y | Y | N/A | Y | Y | N/A | Y | Y | Y | Y | N/A | 96 |
| 450 | Female | | | | 56 | | | Iranian | c.860-2A>G | - |  | Familial | Y | N/A | N/A | N/A | N/A | N/A | N/A | N/A | Y | Y | N/A | N/A | N/A | 95 |
| 451 | Female | | | | 23 | | | Iranian | c.860-2A>G | - |  | Familial | Y | N/A | N/A | N/A | N/A | N/A | N/A | N/A | Y | Y | N/A | N/A | N/A | 95 |
| 452 | Male | | | | 26 | | | Iranian | c.860-2A>G | - |  | Familial | Y | N/A | N/A | N/A | N/A | N/A | N/A | N/A | Y | Y | N/A | N/A | N/A | 95 |
| 453 | Male | | | | 28 | | | Iranian | c.860-2A>G | - |  | Familial | Y | N/A | N/A | N/A | N/A | N/A | N/A | N/A | Y | Y | N/A | N/A | N/A | 95 |
| 454 | Male | | | | 31 | | | Iranian | c.860-2A>G | - |  | Familial | Y | N/A | N/A | N/A | N/A | N/A | N/A | N/A | Y | Y | N/A | N/A | N/A | 95 |
| 455 | Female | | | | 6M | | | Iranian | c.860-2A>G | - |  | Familial | Y | N/A | N/A | N/A | N/A | N/A | N/A | N/A | Y | Y | N/A | N/A | N/A | 95 |
| 456 | Male | | | | 3 | | | N/A | c.864insC | p.Arg289Glnfs*12 | NR | Maternal | Y | Y | N/A | N/A | N/A | N/A | N/A | N/A | N/A | N/A | N/A | N/A | N/A | 98 |
| 457 | Male | | | | 2Y 6M | | | N/A | c.864insC | p.Arg289Glnfs*12 |  | Maternal | N | Y | N/A | N/A | N/A | N/A | N/A | N/A | N/A | N/A | N/A | N/A | N/A | 98 |
| 458 | Male | | | | 2M | | | N/A | c.864insC | p.Arg289Glnfs*12 |  | Maternal | N | Y | N/A | N/A | N/A | N/A | N/A | N/A | N/A | N/A | N/A | N/A | N/A | 98 |
| 459 | N/A | | | | N/A | | | Danish | c.868C>T | p.Gln290* | Pathogenic | Familial | N | N/A | Y | N/A | N/A | N/A | N/A | N/A | Y | N/A | N/A | N/A | N/A | 25 |
| 460 | N/A | | | | N/A | | | Danish | c.868C>T | p.Gln290* |  | Familial | N | N/A | Y | N/A | N/A | N/A | N/A | N/A | Y | N/A | N/A | N/A | N/A | 25 |
| 461 | N/A | | | | N/A | | | Italian | c.874_875delCA | p.Gln292Valfs*8 | Pathogenic | Familial | N/A | Y | N/A | N/A | N/A | N/A | N/A | N/A | N/A | Y | N/A | N/A | N/A | 4 |
| 462 | Female | | | | 41 | | | N/A | c.874_875delCA | p.Gln292Valfs*8 |  | N/A | Y | Y | Y | Y | N/A | Y | N/A | Y | Y | Y | N | N | N/A | 71 |
| 463 | Male | | | | 19 | | | N/A | c.874_875delCA | p.Gln292Valfs*8 |  | Maternal | Y | Y | Y | Y | N/A | Y | N/A | Y | Y | Y | N | N | N/A | 71 |
| 464 | Male | | | | 17 | | | N/A | c.874_875delCA | p.Gln292Valfs*8 |  | Maternal | Y | Y | Y | Y | N/A | Y | N/A | Y | Y | Y | N | N | N/A | 71 |
| 465 | N/A | | | | N/A | | | Caucasian | c.878C>T | p.Gln290* | NR | N/A | N/A | Y | N/A | N/A | N/A | N/A | N/A | N/A | Y | Y | N/A | N/A | N/A | 24 |
| 466 | N/A | | | | N/A | | | N/A | c.879_885del | p.Ser294Argfs*12 | NR | N/A | N/A | N/A | N/A | N/A | N/A | N/A | N/A | N/A | N/A | N/A | N/A | N/A | N/A | 14 |
| 467 | N/A | | | | N/A | | | N/A | c.884delC | p.Pro295Argfs*13 | Pathogenic | Familial | N/A | Y | N/A | N/A | N/A | N/A | N/A | N/A | N/A | Y | N/A | N/A | N/A | 7 |
| 468 | Female | | | | 16 | | | Chinese | c.884delC | p.Pro295Argfs*13 |  | Maternal | Y | Y | N/A | Y | N/A | Y | N/A | Y | Y | Y | Y | Y | N/A | 54 |
| 469 | Female | | | | N/A | | | Chinese | c.884delC | p.Pro295Argfs*13 |  | N/A | N/A | Y | N/A | Y | N/A | Y | N/A | N/A | Y | Y | Y | Y | N/A | 54 |
| 470 | Female | | | | 39 | | | Chinese | c.887insC | p.Trp297Valfs*4 | NR | N/A | N/A | Y | Y | N/A | N/A | N/A | N/A | N/A | Y | Y | Y | N/A | N/A | 6 |
| 471 | Female | | | | 14 | | | Chinese | c.887insC | p.Trp297Valfs*4 |  | Maternal | N/A | Y | Y | N/A | N/A | N | N/A | N/A | Y | Y | N | Y | N/A | 6 |
| 472 | N/A | | | | N/A | | | N/A | c.887delC | p.Pro296Argfs*12 | NR | N/A | N/A | N/A | Y | N/A | N/A | N/A | N/A | N/A | N/A | Y | Y | N/A | N/A | 13 |
| 473 | N/A | | | | N/A | | | N/A | c.891G>A | p.Trp297* | Pathogenic | N/A | N/A | N/A | N/A | N/A | N/A | N/A | N/A | N/A | N/A | N/A | N/A | N/A | N/A | 41 |
| 474 | N/A | | | | N/A | | | N/A | c.891G>A | p.Trp297* |  | N/A | N/A | N/A | N/A | N/A | N/A | N/A | N/A | N/A | N/A | N/A | N/A | N/A | N/A | 13 |
| 475 | Female | | | | 4 | | | N/A | c.891G>A | p.Trp297* |  | Familial | N | Y | Y | N | N | Y | N/A | N/A | N/A | Y | N | N/A | N/A | 39 |
| 476 | Female | | | | 25 | | | Chinese | c.895T>C | p.Tyr299His | NR | *De novo* | Y | Y | Y | Y | N/A | Y | N/A | Y | Y | Y | Y | Y | N/A | 100 |
| 477 | N/A | | | | N/A | | | N/A | c.897T>G | p.Tyr299* | NR | N/A | Y | Y | Y | N/A | N/A | N/A | N/A | N/A | N/A | Y | N | Y | N/A | 101 |
| 478 | Male | | | | 21 | | | Thai | c.901C>T | p.Gln301* | Pathogenic | Maternal | N/A | Y | N | Y | N/A | N/A | N/A | Y | Y | N | Y | N/A | N/A | 80 |
| 479 | Female | | | | N/A | | | Thai | c.901C>T | p.Gln301* |  | Maternal | N/A | N/A | N | Y | N/A | N/A | N/A | Y | Y | N | N/A | N/A | N/A | 80 |
| 480 | N/A | | | | N/A | | | Chinese | c.909C>A | p.Tyr303* | NR | N/A | N/A | N/A | N/A | N/A | N/A | N/A | N/A | N/A | N/A | N/A | N/A | N/A | N/A | 67 |
| 481 | Male | | | | 3 | | | Chinese | c.909C>G | p.Tyr303* | Pathogenic | Paternal | Y | Y | Y | Y | N/A | Y | Y | N/A | Y | Y | Y | Y | Y | 18 |
| 482 | Male | | | | N/A | | | Chinese | c.909C>G | p.Tyr303* |  | *De novo* | N/A | N/A | N/A | N/A | N/A | N/A | N/A | N/A | N/A | N/A | N/A | N/A | N/A | 18 |
| 483 | N/A | | | | N/A | | | N/A | c.915delC | p.Tyr306Thrfs*2 | NR | N/A | N/A | N/A | N/A | N/A | N/A | N/A | N/A | N/A | N/A | N/A | N/A | N/A | N/A | 13 |
| 484 | Male | | | | N/A | | | Chinese | c.938C>T | p.Pro313Leu | NR | Paternal | N/A | N/A | N/A | N/A | N/A | N/A | N/A | N/A | Y | N/A | N/A | N/A | N/A | 102 |
| 485 | Male | | | | N/A | | | Chinese | c.938C>T | p.Pro313Leu |  | N/A | N/A | N/A | N/A | N/A | N/A | N/A | N/A | N/A | N/A | N/A | N/A | N/A | N/A | 102 |
| 486 | Male | | | | 33 | | | Caucasian | c.938delC | p.Pro313Argfs*171 | Pathogenic | *De novo* | N/A | N/A | Y | Y | N/A | N/A | N/A | N/A | Y | N/A | N/A | N/A | N/A | 12 |
| 487 | Female | | | | 10Y6M | | | N/A | c.947delA | p.His316Profs*168 | NR | *De novo* | N/A | Y | Y | N/A | N/A | Y | Y | N/A | Y | Y | N | N/A | Y | 103 |
| 488 | Male | | | | 12M | | | N/A | c.950_971del | p.Ser317Tyrfs*160 | NR | N/A | N/A | N/A | Y | N/A | N/A | Y | Y | N/A | N/A | Y | Y | N/A | N/A | 97 |
| 489 | N/A | | | | N/A | | | N/A | c.977delG | p.Gly326Alafs*158 | NR | N/A | N/A | N/A | N/A | N/A | N/A | N/A | N/A | N/A | N/A | N/A | N/A | N/A | N/A | 14 |
| 490 | N/A | | | | N/A | | | Italian | c.1000G>A | p.Asp334Asn | Uncertain significance | N/A | N/A | N/A | N/A | N/A | N/A | N/A | N/A | N/A | N/A | N/A | N/A | N/A | N/A | 22 |
| 491 | N/A | | | | N/A | | | N/A | c.1019del | p.Ser340* | NR | N/A | N/A | Y | Y | N/A | N/A | N/A | N/A | N/A | N/A | Y | N | N/A | N/A | 101 |
| 492 | N/A | | | | N/A | | | N/A | c.1056G>A | p.Trp352* | NR | N/A | N/A | N/A | N/A | N/A | N/A | N/A | N/A | N/A | N/A | N/A | N/A | N/A | N/A | 14 |
| 493 | Female | | | | 25 | | | Chinese | c.1061insT | p.Ser354Phefs*5 | NR | Maternal | Y | N/A | Y | Y | N/A | Y | Y | Y | Y | N/A | N/A | N/A | N/A | 69 |
| 494 | Female | | | | 48 | | | Chinese | c.1061insT | p.Ser354Phefs*5 |  | Maternal | N/A | N/A | N/A | N/A | N/A | N/A | N/A | N/A | N/A | N/A | N/A | N/A | N/A | 69 |
| 495 | Male | | | | 40 | | | Chinese | c.1061insT | p.Ser354Phefs*5 |  | Maternal | N/A | N/A | N/A | N/A | N/A | N/A | N/A | N/A | N/A | N/A | N/A | N/A | N/A | 69 |
| 496 | Male | | | | 14 | | | Chinese | c.1061insT | p.Ser354Phefs*5 |  | Paternal | N/A | N/A | N/A | N/A | N/A | N/A | N/A | N/A | N/A | N/A | N/A | N/A | N/A | 69 |
| 497 | Female | | | | 71 | | | Chinese | c.1061insT | p.Ser354Phefs*5 |  | N/A | N/A | N/A | N/A | N/A | N/A | N/A | N/A | N/A | N/A | N/A | N/A | N/A | N/A | 69 |
| 498 | Female | | | | N/A | | | N/A | c.1064C>T | p.Thr355Ile | NR | N/A | N/A | N/A | N/A | N/A | N/A | N/A | N/A | N/A | N | N/A | N/A | N/A | N/A | 105 |
| 499 | Female | | | | 6 | | | Thai | c.1081C>T | p.Gln361* | Pathogenic | N/A | Y | Y | Y | Y | N/A | Y | N/A | N/A | N/A | Y | Y | Y | N/A | 80 |
| 500 | N/A | | | | N/A | | | Japanese | c.1085C>T | p.Ala362Val | Pathogenic | N/A | N/A | N/A | N/A | N/A | N/A | N/A | N/A | N/A | N | N/A | N/A | N/A | N/A | 9 |
| 501 | N/A | | | | N/A | | | N/A | c.1088-1G>C | - | NR | N/A | N/A | N/A | N/A | N/A | N/A | N/A | N/A | N/A | N/A | N/A | N/A | N/A | N/A | 14 |
| 502 | Male | | | | 38 | | | Turkish | c.1088G>T | p.Gly363Val | Likely pathogenic | Familial | Y | N/A | Y | Y | N/A | N | N/A | N/A | Y | Y | Y | N | N/A | 2 |
| 503 | Female | | | | 8 | | | Turkish | c.1088G>T | p.Gly363Val |  | Familial | Y | N | Y | Y | N/A | N | N/A | Y | Y | Y | Y | N | N/A | 2 |
| 504 | Female | | | | 2 | | | Turkish | c.1088G>T | p.Gly363Val |  | Familial | Y | N | Y | Y | N/A | N | N/A | Y | N | Y | Y | N | N/A | 2 |
| 505 | Female | | | | 28 | | | Chinese | c.1096G>T | p.Glu366* | NR | N/A | N/A | Y | N/A | N/A | N/A | N/A | N/A | N/A | Y | Y | N/A | N/A | N/A | 106 |
| 506 | Male | | | | 23 | | | Chinese | c.1096G>T | p.Glu366* |  | N/A | N/A | Y | N/A | N/A | N/A | N/A | N/A | N/A | Y | Y | N/A | N/A | N/A | 106 |
| 507 | Female | | | | 18 | | | Chinese | c.1111dupT | p.Ser371Phefs*14 | NR | *De novo* | Y | N/A | Y | Y | N/A | N/A | N/A | Y | Y | Y | N/A | N/A | N/A | 107 |
| 508 | N/A | | | | N/A | | | Italian | c.1111_1129del19 | p.Ser371Glnfs*107 | NR | *De novo* | Y | Y | N/A | N/A | N/A | N/A | N/A | N/A | N/A | Y | N/A | N/A | N/A | 4 |
| 509 | Male | | | | 23 | | | Chinese | c.1119insC | p.Arg374Glnfs*11 | NR | *De novo* | N/A | Y | Y | Y | N/A | N/A | Y | Y | Y | Y | N/A | Y | N/A | 108 |
| 510 | Male | | | | 11 | | | Taiwanese | c.1119delC | p.Arg374Glyfs*110 | NR | *De novo* | Y | N/A | Y | N/A | N/A | N/A | N/A | N/A | Y | Y | Y | N/A | N/A | 53 |
| 511 | Male | | | | 12 | | | N/A | c.1119delC | p.Arg374Glyfs*110 |  | *De novo* | N/A | N/A | N/A | N/A | N/A | N/A | N/A | Y | Y | Y | N/A | N/A | N/A | 81 |
| 512 | Male | | | | 13 | | | N/A | c.1119delC | p.Arg374Glyfs*110 |  | *De novo* | N | Y | Y | Y | N | Y | N/A | N/A | N/A | Y | Y | N/A | N/A | 39 |
| 513 | N/A | | | | N/A | | | Danish | c.1122delG | p.Arg374Serfs*110 | NR | Familial | Y | N/A | N/A | N/A | N/A | N/A | N/A | N/A | N/A | N/A | N/A | N/A | N/A | 25 |
| 514 | N/A | | | | N/A | | | Danish | c.1122delG | p.Arg374Serfs*110 |  | Familial | Y | N/A | Y | N/A | N/A | N/A | N/A | N/A | Y | N/A | N/A | N/A | N/A | 25 |
| 515 | N/A | | | | N/A | | | Danish | c.1122delG | p.Arg374Serfs*110 |  | Familial | N | N/A | Y | N/A | N/A | N/A | N/A | N/A | Y | N/A | N/A | N/A | N/A | 25 |
| 516 | Male | | | | 26 | | | Japanese | c.1124insA | p.Phe376Valfs*9 | NR | *De novo* | Y | Y | N/A | N/A | N/A | N/A | N/A | N/A | Y | N | N/A | N/A | N/A | 29 |
| 517 | N/A | | | | N/A | | | N/A | c.1127insT | p.Ser378Lysfs*7 | NR | N/A | N/A | N/A | N/A | N/A | N/A | N/A | N/A | N/A | N/A | N/A | N/A | N/A | N/A | 13 |
| 518 | N/A | | | | N/A | | | Danish | c.1132delA | p.Ser378Alafs*106 | NR | *De novo* | Y | N/A | N/A | N/A | N/A | N/A | N/A | N/A | Y | N/A | N/A | N/A | N/A | 25 |
| 519 | N/A | | | | N/A | | | N/A | c.1153_1154delGA | p.Ser385Profs*104 | NR | N/A | N/A | N/A | N/A | N/A | N/A | N/A | N/A | N/A | N/A | N/A | N/A | N/A | N/A | 14 |
| 520 | N/A | | | | N/A | | | N/A | c.1157delG | p.Arg386Profs*98 | NR | N/A | N/A | N/A | N/A | N/A | N/A | N/A | N/A | N/A | N/A | N/A | N/A | N/A | N/A | 13 |
| 521 | Female | | | | 19 | | | Caucasian | c.1157delG | p.Arg386Profs*98 |  | *De novo* | N/A | N/A | Y | Y | N/A | N/A | N/A | N/A | Y | N/A | N/A | N/A | N/A | 12 |
| 522 | N/A | | | | N/A | | | N/A | c.1169delC | p.Pro390Hisfs*94 | NR | N/A | N/A | N/A | N/A | N/A | N/A | N/A | N/A | N/A | N/A | N/A | N/A | N/A | N/A | 14 |
| 523 | N/A | | | | N/A | | | N/A | c.1171C>T | p.Arg391* | Pathogenic | *De novo* | N/A | Y | N/A | N/A | N/A | N/A | N/A | N/A | N/A | Y | N/A | N/A | N/A | 7 |
| 524 | N/A | | | | N/A | | | Japanese | c.1171C>T | p.Arg391* |  | N/A | N/A | N/A | N/A | N/A | N/A | N/A | N/A | N/A | N/A | N/A | N/A | N/A | N/A | 5 |
| 525 | Male | | | | 4 | | | Taiwanese | c.1171C>T | p.Arg391* |  | *De novo* | Y | Y | Y | N/A | N/A | Y | Y | N/A | N/A | Y | N/A | Y | N/A | 109 |
| 526 | 525 | | | | Male | | | Italian | c.1171C>T | p.Arg391* |  | *De novo* | Y | Y | N/A | N/A | N/A | N/A | N/A | N/A | N/A | Y | N/A | N/A | N/A | 4 |
| 527 | N/A | | | | N/A | | | Caucasian | c.1171C>T | p.Arg391* |  | N/A | N/A | Y | N/A | N/A | N/A | N/A | N/A | N/A | N/A | Y | N/A | N/A | N/A | 24 |
| 528 | N/A | | | | N/A | | | Caucasian | c.1171C>T | p.Arg391* |  | N/A | N/A | Y | N/A | N/A | N/A | N/A | N/A | N/A | N/A | Y | N/A | N/A | N/A | 24 |
| 529 | N/A | | | | N/A | | | Caucasian | c.1171C>T | p.Arg391* |  | N/A | N/A | Y | N/A | N/A | N/A | N/A | N/A | N/A | N/A | Y | N/A | N/A | N/A | 24 |
| 530 | N/A | | | | N/A | | | Caucasian | c.1171C>T | p.Arg391* |  | N/A | N/A | Y | N/A | N/A | N/A | N/A | N/A | N/A | N/A | Y | N/A | N/A | N/A | 24 |
| 531 | N/A | | | | N/A | | | Caucasian | c.1171C>T | p.Arg391* |  | N/A | N/A | Y | N/A | N/A | N/A | N/A | N/A | N/A | N/A | Y | N/A | N/A | N/A | 24 |
| 532 | N/A | | | | N/A | | | Korean | c.1171C>T | p.Arg391* |  | Familial | N/A | N/A | N/A | N/A | N/A | N/A | N/A | N/A | N | N/A | N/A | N/A | N/A | 3 |
| 533 | Female | | | | 27 | | | Chinese | c.1171C>T | p.Arg391* |  | N/A | N/A | Y | N/A | N/A | N/A | N/A | N/A | N/A | Y | Y | N/A | N/A | N/A | 38 |
| 534 | Male | | | | 15 | | | Taiwanese | c.1171 C>T | p.Arg391* |  | *De novo* | Y | N/A | Y | N/A | N/A | N/A | N/A | N/A | Y | Y | Y | N/A | N/A | 53 |
| 535 | Male | | | 4 | | | | N/A | c.1171C>T | p.Arg391* |  | *De novo* | N/A | N/A | N/A | N/A | N/A | N/A | N/A | N/A | Y | Y | N/A | N/A | N/A | 81 |
| 536 | Female | | | 8 | | | | Korean | c.1171C>T | p.Arg391* |  | N/A | N/A | N/A | N/A | N/A | N/A | N/A | N/A | N/A | N/A | N/A | N/A | N/A | N/A | 46 |
| 537 | Female | | | 2 | | | | N/A | c.1171C>T | p.Arg391* |  | *De novo* | N | Y | Y | Y | N | Y | N/A | N/A | Y | Y | N | N/A | N/A | 39 |
| 538 | Female | | | 8 | | | | N/A | c.1171C>T | p.Arg391* |  | *De novo* | Y | Y | Y | Y | N | Y | N/A | N/A | Y | Y | Y | Y | N/A | 39 |
| 539 | Female | | | 25 | | | | Turkish | c.1171C>T | p,Arg391* |  | Familial | Y | Y | Y | Y | Y | Y | Y | N/A | Y | Y | Y | Y | N/A | 88 |
| 540 | Male | | | 58 | | | | Turkish | c.1171C>T | p,Arg391* |  | Familial | Y | Y | Y | Y | Y | Y | N/A | N/A | Y | Y | Y | Y | N/A | 88 |
| 541 | Female | | | 70 | | | | Turkish | c.1171C>T | p,Arg391* |  | Familial | Y | Y | Y | Y | N | N | N/A | N/A | Y | Y | Y | Y | N/A | 88 |
| 542 | Male | | | 14 | | | | N/A | c.1182T>A | p.Tyr394* | Pathogenic | Maternal | Y | Y | Y | N/A | N/A | Y | Y | Y | Y | Y | Y | Y | N/A | 110 |
| 543 | Male | | | 26 | | | | N/A | c.1199-1200insA | p.Tyr400* | NR | *De novo* | N/A | Y | N/A | N/A | N/A | N/A | N/A | N/A | Y | Y | N/A | N/A | N/A | 64 |
| 544 | N/A | | | N/A | | | | N/A | c.1205delC | p.Pro402Argfs*82 | NR | N/A | N/A | N/A | N/A | N/A | N/A | N/A | N/A | N/A | N/A | N/A | N/A | N/A | N/A | 14 |
| 545 | N/A | | | N/A | | | | N/A | c.1205dupC | p.Pro403Alafs*87 | NR | N/A | N/A | N/A | N/A | N/A | N/A | N/A | N/A | N/A | N/A | Y | Y | N/A | N/A | 13 |
| 546 | Male | | | 3Y | | | | N/A | c.1215dupC | p.Ser406Leufs*84 | NR | N/A | N/A | N/A | Y | Y | N/A | N/A | N/A | Y | N/A | Y | Y | N/A | N/A | 97 |
| 547 | N/A | | | 20 week | | | | N/A | c.1228dupC | p.Leu410Profs*80 | Pathogenic | N/A | N/A | N/A | N/A | N/A | N/A | N/A | N/A | N/A | N/A | N/A | N/A | N/A | N/A | 111 |
| 548 | N/A | | | N/A | | | | N/A | c.1246_1247insACCC | p.Thr416Asnfs*75 | NR | *De novo* | N/A | N/A | N/A | N/A | N/A | N/A | N/A | N/A | N/A | Y | Y | N/A | N/A | 114 |
| 549 | N/A | | | N/A | | | | Caucasian | c.1250insA | p.His417Glnfs*73 | NR | N/A | N/A | Y | N/A | N/A | N/A | N/A | N/A | N/A | Y | Y | N/A | N/A | N/A | 24 |
| 550 | N/A | | | N/A | | | | N/A | c.1250_1251delCA | p.His417Leufs*72 | NR | N/A | N/A | N/A | N/A | N/A | N/A | N/A | N/A | N/A | N/A | N/A | N/A | N/A | N/A | 14 |
| 551 | N/A | | | N/A | | | | N/A | c.1254C>A | p.Tyr418* | NR | N/A | N/A | N/A | N/A | N/A | N/A | N/A | N/A | N/A | N/A | N/A | N/A | N/A | N/A | 14 |
| 552 | N/A | | | N/A | | | | N/A | c.1259C>A | p.Thr420Asn | NR | N/A | N/A | N/A | N/A | N/A | N/A | N/A | N/A | N/A | N/A | N/A | N/A | N/A | N/A | 14 |
| 553 | Female | | | 2Y 6M | | | | Chinese | c.1259C>T | p.Thr420Ile | NR | Maternal | N/A | Y | Y | N/A | N/A | N/A | Y | N/A | Y | Y | N | N | N/A | 61 |
| 554 | Male | | | 11M | | | | Chinese | c.1259C>T | p.Thr420Ile |  | Maternal | N/A | Y | N/A | N/A | N/A | Y | N/A | N/A | Y | Y | N/A | N/A | N/A | 61 |
| 555 | Female | | | 32 | | | | Chinese | c.1259C>T | p.Thr420Ile |  | *De novo* | N/A | Y | Y | N/A | N/A | N/A | N/A | N/A | Y | Y | N/A | N/A | N/A | 61 |
| 556 | Male | | | 29 | | | | N/A | c.1259C>T | p.Thr420Ile |  | *De novo* | Y | N/A | Y | Y | N/A | Y | N/A | N/A | Y | Y | N/A | N/A | N/A | 113 |
| 557 | Male | | | 5 | | | | N/A | C.1271_1272insTACCTACCTGTACACTCACT | p.Pro425Thrfs*66 | NR | *De novo* | Y | Y | Y | Y | N/A | Y | N/A | N/A | Y | Y | Y | N/A | N/A | 39 |
| 558 | Female | | | 12M | | | | Turkish | c.1281delC | p.Gly428Alafs*56 | Pathogenic | Familial | N/A | Y | Y | Y | N/A | Y | N/A | Y | N | Y | N | N | N/A | 2 |
| 559 | Female | | | 24 | | | | Turkish | c.1281delC | p.Gly428Alafs*56 |  | Familial | N/A | Y | Y | Y | N/A | Y | N/A | Y | Y | Y | N | Y | N/A | 2 |
| 560 | Male | | | 12 | | | | Turkish | c.1281delC | p.Gly428Alafs*56 |  | Familial | N/A | Y | Y | Y | N/A | N | N/A | N | Y | Y | N | N | N/A | 2 |
| 561 | Male | | | 14 | | | | Turkish | c.1281delC | p.Gly428Alafs*56 |  | Familial | N/A | Y | N | Y | N/A | Y | N/A | Y | Y | Y | Y | N | N/A | 2 |
| 562 | Female | | | 42 | | | | Turkish | c.1281delC | p.Gly428Alafs*56 |  | Familial | N/A | N/A | N | Y | N/A | Y | N/A | Y | Y | N | Y | N | N/A | 2 |
| 563 | N/A | | | N/A | | | | Chinese | c.1321dupT | p.Ser370Phefs*13 | NR | N/A | N/A | N/A | N/A | N/A | N/A | N/A | N/A | N/A | N/A | N/A | N/A | N/A | N/A | 17 |
| 564 | N/A | | | N/A | | | | N/A | c.1335_1336delTC | p.Tyr446Leufs*43 | NR | N/A | N/A | N/A | N/A | N/A | N/A | N/A | N/A | N/A | N/A | N/A | N/A | N/A | N/A | 14 |
| 565 | N/A | | | N/A | | | | Italian | c.1361A>G | p.Tyr454Cys | Uncertain significance | N/A | N/A | N/A | N/A | N/A | N/A | N/A | N/A | N/A | N/A | N/A | N/A | N/A | N/A | 22 |
| 566 | N/A | | | N/A | | | | N/A | c.1379dupC | p.Asp463Argfs*27 | Pathogenic/Likely pathogenic | N/A | N/A | N/A | N/A | N/A | N/A | N/A | N/A | N/A | N/A | N/A | N/A | N/A | N/A | 13 |
| 567 | Male | | | 16 | | | | Asian | c.1381G>C | p.Gly461Arg | NR | Familial | N | N/A | N | N | N/A | N/A | N/A | Y | Y | N/A | N/A | N/A | N/A | 28 |
| 568 | Male | | | 14 | | | | Korean | c.1384G>T | p.Gly462* | NR | N/A | N/A | Y | Y | N/A | N/A | N/A | N/A | N/A | Y | Y | N/A | N/A | N/A | 46 |
| 569 | N/A | | | N/A | | | | N/A | c.1385dupG | p.Asp463Argfs*27 | Pathogenic | N/A | N/A | N/A | N/A | N/A | N/A | N/A | N/A | N/A | N/A | N/A | N/A | N/A | N/A | 14 |
| 570 | N/A | | | N/A | | | | Italian | c.1531G>A | p.Gly511Ser | Benign/Likely benign | N/A | N/A | N/A | N/A | N/A | N/A | N/A | N/A | N/A | N/A | N/A | N/A | N/A | N/A | 22 |
| 571 | N/A | | | N/A | | | | Caucasian | c.1550_1553delTTTG | p.Val517Glyfs*61 | Pathogenic | N/A | N/A | Y | N/A | N/A | N/A | N/A | N/A | N/A | Y | Y | N/A | N/A | N/A | 24 |
| 572 | Female | | | 19 | | | | Caucasian | c.1550_1553delTTTG | p.Val517Glyfs*61 |  | *De novo* | N/A | N/A | Y | Y | N/A | N/A | N/A | N/A | Y | N/A | N/A | N/A | N/A | 12 |
| 573 | Male | | | 23 | | | | Turkish | c.1550_1553delTTTG | p.Val517Glyfs*61 |  | Paternal | N | Y | N/A | N/A | N/A | Y | Y | N/A | Y | N/A | Y | Y | N/A | 115 |
| 574 | Male | | | 18 | | | | Turkish | c.1550_1553delTTTG | p.Val517Glyf*61 |  | Paternal | Y | Y | Y | N/A | Y | Y | N/A | N/A | N/A | Y | N/A | N/A | N/A | 115 |
| 575 | Male | | 52 | | | | | Turkish | c.1550_1553delTTTG | p.Val517Glyfs*61 |  | N/A | Y | Y | Y | N/A | N/A | Y | Y | N/A | Y | N/A | N/A | N/A | N/A | 115 |
| 576 | Male | | 10 | | | | | N/A | c.1550_1553delTTTG | p.Val517Glyfs*61 |  | *De novo* | N | Y | Y | Y | N | Y | N/A | N/A | Y | N | N | N/A | N/A | 39 |
| 577 | Male | | 15 | | | | | N/A | c.1552delT | p.Trp518Glyfs*60 | NR | *De novo* | Y | N/A | N/A | Y | N/A | N/A | N/A | N/A | Y | Y | Y | N/A | N/A | 116 |
| 578 | Male | | 10 | | | | | Chinese | c.1554delG | p.Trp518Cysfs*61 | Pathogenic | Maternal | N | Y | Y | Y | N/A | N/A | N/A | N/A | Y | Y | Y | Y | N/A | 117 |
| 579 | Female | | N/A | | | | | Chinese | c.1554delG | p.Trp518Cysfs*61 |  | N/A | N/A | N/A | Y | N/A | N/A | N/A | N/A | N/A | Y | Y | Y | Y | N/A | 117 |
| 580 | Male | | 16 | | | | | Mexican | c.1565G>C | p.*522Serext*23 | Pathogenic | *De novo* | Y | Y | Y | Y | Y | Y | Y | Y | Y | Y | N/A | Y | N/A | 10 |
| 581 | Female | | 2 | | | | | Taiwanese | - | exon 0~7, 3’UTR deletion | NR | *De novo* | Y | N/A | Y | N/A | N/A | N/A | N/A | N/A | Y | Y | Y | N/A | N/A | 53 |
| 582 | Female | | 5 | | | | | Caucasian | - | exon 1 to exon 3 deletion | NR | Paternal | N | Y | Y | Y | N | Y | N/A | N/A | Y | Y | N/A | N/A | N/A | 118 |
| 583 | Male | | 35 | | | | | Caucasian | - | exon 1 to exon 3 deletion | NR | Maternal | Y | Y | N/A | Y | N/A | N/A | N/A | N/A | Y | Y | N/A | N/A | N/A | 118 |
| 584 | Female | | 80 | | | | | Caucasian | - | exon 1 to exon 3 deletion | NR | N/A | N | Y | N/A | Y | N/A | N/A | N/A | N/A | Y | Y | N/A | N/A | N/A | 118 |
| 585 | N/A | | N/A | | | | | Danish | - | exon 1 to exon 5 deletion | NR | Familial | N | N/A | N/A | N/A | N/A | N/A | N/A | N/A | N/A | N/A | N/A | N/A | N/A | 25 |
| 586 | N/A | | N/A | | | | | Danish | - | exon 1 to exon 5 deletion | NR | Familial | Y | N/A | Y | N/A | N/A | N/A | N/A | N/A | Y | N/A | N/A | N/A | N/A | 25 |
| 587 | N/A | | N/A | | | | | Danish | - | exon 1 to exon 5 deletion | NR | Familial | Y | N/A | N/A | N/A | N/A | N/A | N/A | N/A | Y | N/A | N/A | N/A | N/A | 25 |
| 588 | N/A | | N/A | | | | | Danish | - | exon 1 to exon 5 deletion | NR | Familial | Y | N/A | Y | N/A | N/A | N/A | N/A | N/A | Y | N/A | Y | N/A | N/A | 25 |
| 589 | N/A | | N/A | | | | | Danish | - | exon 1 to exon 5 deletion | NR | *De novo* | N/A | N/A | N/A | N/A | N/A | N/A | N/A | N/A | N/A | N/A | N/A | N/A | N/A | 25 |
| 590 | Male | | 13 | | | | | Caucasian | - | exon 2 deletion | NR | *De novo* | N/A | Y | Y | Y | N/A | Y | N/A | N/A | Y | Y | Y | N/A | N/A | 119 |
| 591 | Male | | N/A | | | | | N/A | - | exon 2 to exon 6 deletion | NR | Paternal | Y | Y | Y | N/A | N/A | N/A | N/A | N/A | N/A | Y | N/A | Y | N/A | 120 |
| 592 | Male | | N/A | | | | | N/A | - | exon 2 to exon 6 deletion | NR | Paternal | Y | Y | Y | N/A | N/A | N/A | N/A | N/A | N/A | Y | N/A | Y | N/A | 120 |
| 593 | Male | | N/A | | | | | N/A | - | exon 2 to exon 6 deletion | NR | Paternal | Y | Y | Y | N/A | N/A | N/A | N/A | N/A | N/A | Y | N/A | Y | N/A | 120 |
| 594 | Male | | N/A | | | | | N/A | - | exon 2 to exon 6 deletion | NR | Paternal | Y | Y | Y | N/A | N/A | N/A | N/A | N/A | N/A | Y | N/A | Y | N/A | 120 |
| 595 | Male | | N/A | | | | | N/A | - | exon 2 to exon 6 deletion | NR | Paternal | Y | Y | Y | N/A | N/A | N/A | N/A | N/A | N/A | Y | N/A | Y | N/A | 120 |
| 596 | Male | | N/A | | | | | N/A | - | exon 2 to exon 6 deletion | NR | Paternal | Y | Y | Y | N/A | N/A | N/A | N/A | N/A | N/A | Y | N/A | Y | N/A | 120 |
| 597 | Male | | N/A | | | | | N/A | - | exon 2 to exon 6 deletion | NR | Paternal | Y | Y | Y | N/A | N/A | N/A | N/A | N/A | N/A | Y | N/A | Y | N/A | 120 |
| 598 | Female | | N/A | | | | | N/A | - | exon 2 to exon 6 deletion | NR | Paternal | Y | Y | Y | N/A | N/A | N/A | N/A | N/A | N/A | Y | N/A | Y | N/A | 120 |
| 599 | Female | | 16 | | | | | Turkish | - | exon 2 to exon 9 deletion | NR | *De novo* | N/A | N | N | Y | N/A | Y | N/A | Y | N | Y | Y | Y | N/A | 2 |
| 600 | Female | | 4M | | | | | Turkish | - | exon 3 to exon 9 deletion | NR | *De novo* | N/A | Y | Y | Y | N/A | Y | N/A | N | N/A | Y | N | Y | N/A | 2 |
| 601 | Female | | 27 | | | | | Turkish | - | exon 6 to exon 9 deletion | NR | *De novo* | N/A | Y | Y | Y | N/A | N | N/A | N | N | Y | Y | N | N/A | 2 |
| 602 | Male | | 6 | | | | | Caucasian | - | exon 2 to exon 9 deletion | NR | *De novo* | Y | Y | Y | N/A | Y | Y | Y | Y | Y | Y | Y | Y | N/A | 121 |
| 603 | Male | | 18 | | | | | Chinese | - | exon 4 to intron 7 deletion | NR | *De novo* | N/A | Y | Y | Y | N/A | N/A | N/A | N/A | Y | Y | N/A | N/A | N/A | 122 |
| 604 | Male | | 21 | | | | | Chinese | - | 498 kb deletion | NR | *De novo* | N/A | N/A | N/A | Y | N/A | N/A | Y | N/A | Y | Y | N/A | N/A | N/A | 123 |
| 605 | Female | 11M | | | | | | Italian | - | 344 kb deletion | NR | N/A | N/A | Y | N/A | Y | N/A | N/A | N/A | N/A | N/A | Y | N/A | N/A | N/A | 124 |

Y indicates yes; N indicates no; N/A indicates not available

Head1 symptoms: fontanelle open or delayed closure of the anterior fontalle

Head2 symptoms: Bossing of Frontal bone, Bossing of occipital bone, Bossing of parietal bone, Wormian bones, Macrocephaly, Microcephaly, or/and Brachycephaly

Face symptoms: Face: Midface hypoplasia, Metopic groove, or/and Micrognathia

Ears symptom: Hearing loss, Eyes symptoms: Hypertelorism, Nose symptom: or/and Low nasal bridge

Mouse symptoms: Narrow and high-arched palate, Cleft palate or/and Malocclusion

Teeth symptoms: Delayed eruption of deciduous teeth, Delayed eruption of permanent teeth, Supernumerary teeth, Prolonged retention of deciduous teeth, Enamel hypoplasia, Tooth defect, Cyst, or/and Tooth missing

Chest symptoms: Narrow thorax, Abnormal facility in opposing the shoulders, Pectus Excavatum, Hypoplastic clavicles/Aplastic clavicles or/and Short ribs

Skeletal symptoms: Scoliosis, Lordosis, Pubic bone defected, Broad femoral head with short femoral neck, or/and Hypoplasia of femoral head

Finger symptoms: Brachydactyly, Long second metacarpal, Short middle phalanges of second and fifth fingers, Hypoplastic phalanges, Clinodactyly, or/and Defected nail

Neulogical symptoms: Epileptic seizure, or/and Syringomyelia
